# Pre-rRNAs control mitosis by maintaining chromosomal segregation through protecting SMC2 from AURKA-mediated phosphorylation

**DOI:** 10.1038/s41419-025-08169-9

**Published:** 2025-11-07

**Authors:** Shiqi Sun, Kunqi Su, Yang Jiang, Yuying Wang, Yang Hu, Chang Wang, Zhuochen Zhao, Chunfeng Zhang, Baocai Xing, Xiaojuan Du

**Affiliations:** 1 Department of Cell Biology, School of Basic Medical Sciences, Peking University, Beijing, China; 2 Hepatopancreatobiliary Surgery Department I, Key Laboratory of Carcinogenesis and Translational Research (Ministry of Education), Peking University School of Oncology, Beijing Cancer Hospital & Institute, Beijing, China; 3 Department of Medical Genetics, School of Basic Medical Sciences, Peking University, Beijing, China

**Keywords:** Mitosis, Chromosome segregation

## Abstract

In interphase, 47S pre-rRNA is transcribed by RNA polymerase I (Pol I) and processed to form intermediate pre-rRNAs and finally produce mature rRNAs in the nucleolus. During mitosis, nucleolus disassembles and pre-rRNAs including 45S, 30S and 32S pre-rRNAs relocate in the peri-chromosomal region (PR). Inhibition of pre-rRNA transcription impairs chromosome dispersion in prometaphase. However, how pre-rRNAs regulate mitosis remains elusive. Here, we unravel a novel mechanism for pre-rRNAs to control mitosis. Inhibition of Pol I prolongs the mitotic process and induces defective chromosomal segregation, resulting in mitotic catastrophe. We isolated chromosome and determined the chromosome-binding proteins by mass-spectrometry. Using quantitative proteomics analysis, immunoprecipitation and immunofluorescent staining, we found that AURKA approaches chromosome when Pol I is inhibited. The AURKA-binding proteins on the chromosome were determined by immunoprecipitation and mass-spectrometry after cells were treated with Act D, BMH-21 or CX5461, respectively, and the chromosomal segregation controlling proteins were selected. When Pol I was inhibited, the binding of AURKA with SMC2, the crucial component of Condensin, is significantly enhanced. Importantly, SMC2 is phosphorylated by AURKA only when Pol I was inhibited. Alignment of SMC2 amino acid sequence with substrates of AURKA shows that SMC2 possesses the consensus R/K/N-R-X-S/T-B, and T574 is the only potential AURKA-catalyzed phosphorylation site. Indeed, SMC2 T574 is phosphorylated by AURKA in cell and in vitro. Thereafter, we generated SMC2 T574-P specific antibody, and confirmed that endogenous SMC2 T574 is phosphorylated by AURKA in mitosis in the absence of pre-rRNAs. Consequently, phosphorylation of SMC2 T574 disrupts the SMC2/SMC4 binding and the binding of SMC2 and SMC4 to chromosomal DNA, leading to chromosomal segregation defect. The phosphorylation deficient Flag-SMC2 T574A reverses the mitotic catastrophe caused by Pol I inhibition. Collectively, we demonstrate that pre-rRNAs protect SMC2 from the AURKA-mediated phosphorylation to maintain normal mitosis.

## Introduction

Mitosis is the process by which eukaryotic cells organize and segregate their chromosomes into two daughter cells to maintain genomic stability. Chromosomal segregation disorders during cell divisions generate aneuploidies, which can cause miscarriages and developmental syndromes, and are strongly associated with tumourigenesis [1, 2]. In cancer cells, errors in chromosomal segregation often generate multinucleation and ultimately lead to mitotic catastrophe, which is an onco-suppressive mechanism for avoiding genomic instability [3, 4]. Therefore, induction of mitotic catastrophe might constitute a highly desirable therapeutic endpoint for cancers [3].

In eukaryotic cells, correct chromosomal segregation requires the resolution of sister chromosomes and their movement into opposite halves of the cell before cytokinesis. The dynamic changes of chromosomes during these events depend on the action of a multi-subunit SMC (structural maintenance of chromosomes) protein complex named Condensin [5]. Condensin regulates chromosomal structure in a wide range of processes, including chromosomal segregation, gene transcriptional regulation, DNA damage repair, and chromosomal recombination [6]. During interphase, Condensin forms chromosomal loops through their conserved SMC ring structure, which DNA is pushed through [7, 8], while Condensin controls chromosomal assembly during mitosis and meiosis in all eukaryotes [5, 9]. In the process of chromosomal segregation, Condensin facilitates the removal of sister chromatid Cohesin, sister chromatid decatenation, and the structural reconfiguration of mitotic chromosomes [5]. Functional inhibition of Condensin complex causes abnormal chromosomal condensation and segregation [10]. Condensin complex is composed of SMC2/SMC4 heterodimer and three non-SMC regulational subunits, including BRRN1/CAPH, CNAP1/CAPD2, and CAPG [11, 12]. The SMC2/SMC4 dimer is the fundamental functional unit, while the individual SMC2 or SMC4 does not possess complete biological function [13]. In the Condensin complex, the coiled-coil segments of SMC2 and SMC4 closely apposed to one another along their lengths [14]. Among the binding sites between SMC2 and SMC4, the binding of the SMC2 hinge region (aa553-663) with SMC4 hinge region (aa631-751) mediates the binding of Condensin to DNA to maintain chromosomal structure [15]. Therefore, the binding between SMC2 and SMC4, and the function of SMC2/SMC4 are tightly controlled during mitosis [16].

In interphase, 47S precursor ribosomal RNA (pre-rRNA) is transcribed by Pol I from rDNA, and processed to form intermediate pre-rRNAs, including 45S, 32S, and 30S pre-rRNAs, to finally produce mature 28S, 5.8S, and 28S rRNAs [17]. When cells enter mitosis, interphase chromatin condenses into mitotic chromosomes, nuclear envelope breaks down, nucleolus disassembles, and nucleolar components relocate into the peri-chromosomal region (PR). It has been found that pre-rRNAs, especially 45S, 32S, and 30S pre-rRNAs, localize in the PR and cover each mitotic chromosome [18]. Suppression of chromosome-bound pre-rRNAs by Pol I inhibition impairs mitotic chromosome dispersion during prometaphase, indicating that pre-rRNAs might play a role in preventing chromosome clustering and facilitating chromosome separation during mitosis [18]. However, how pre-rRNAs control mitosis remains largely unclear.

PR is the outer layer of condensed chromosomes, mainly composed of nuclear and nucleolar proteins, and various RNA species [19]. In contrast, the inner chromosomal scaffold contains proteins for maintaining the integrity of the condensed chromosomes, including Condensin, Cohesin, etc [20]. A recent analysis combining scanning electron microscopy with advanced proteomics indicates that PR comprises 30%–47% of the entire chromosome volume [21], thus PR might play important roles in mitosis. In the past, PR was supposed to be a binding site for chromosomal passenger proteins [19] and acting as a landing pad to carry and distribute client proteins and RNAs following cell division [22]. Recently, it has been found that PR promotes chromosomal individualization and clustering in mitosis [23–25]. We previously found that U3 snoRNA and DDX21 inter-regulate each other to maintain their liquidity in the PR to control mitosis, and the residence of U3 snoRNA and DDX21 in the PR is dependent on the presence of pre-rRNAs [26]. Besides, more nucleolar proteins, such as NPM, MYBBP1A, Fibrillarin, and NOL11, localize in the PR during mitosis depending on pre-rRNAs [27–29], indicating that pre-rRNAs play a critical role in the formation of the PR. However, as the leading factor in the PR constituents, if and how pre-rRNAs regulate the inner chromosomal scaffold remains unknown.

Cell cycle is driven by protein phosphorylation via a series of protein kinases. Aurora Kinase A (AURKA) is a pivotal kinase during mitosis. AURKA is a member of the Aurora/IPL1-related kinase family of serine/threonine kinases, which regulates centrosomic maturation, the mitotic entrance, bipolar spindle formation and function, and cytokinesis [30]. AURKA begins to accumulate at centrosomes in late S phase, and is activated at the boundary between the G2 and M phases [31]. During mitosis, active AURKA propagates along the mitotic spindle from centrosomes to the midzone to phosphorylate its substrates such as cdc25B [32], Eg5 [33], and CENP-A [34], to control mitosis. Thus, in all metazoans assessed to date, mutation or depletion of AURKA and incorrect spatialization of AURKA causes abnormal spindle assembly, including characteristic monopolar spindle, and weak, sparse, or short astral microtubules [30, 35]. However, how the incorrect spatialization of AURKA disrupts chromosomal segregation remains unknown.

In the present study, we found that inhibition of pre-rRNA transcription results in mitotic catastrophe. Depletion of pre-rRNAs causes abnormal chromosomal condensation and segregation, and prolongs the mitotic time. When pre-rRNA transcription is inhibited, AURKA approaches chromosome and selectively binds SMC2. Mechanistically, SMC2 is phosphorylated by AURKA at T574, which disrupts the SMC2/SMC4 binding and the binding of SMC2/SMC4 with chromosomal DNA, leading to mitotic catastrophe. Our study provides novel insights into the function of pre-rRNAs in mitotic control and evidence for the PR components in protecting the inner chromosomal scaffold from functional disruption. We found a new phosphorylation site on SMC2 by AURKA and a new mechanism by which Condensin complex is regulated through phosphorylation during mitosis.

## Results

### Depletion of pre-rRNAs results in chromosomal segregation disorder and mitotic catastrophe

**Fig. 1:**
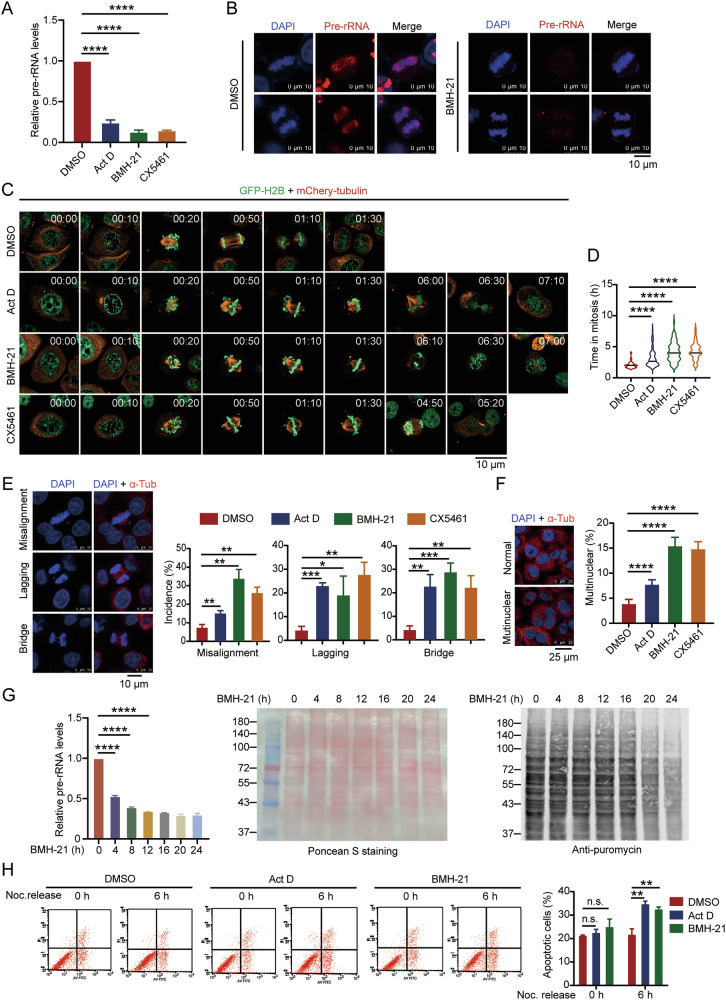
Depletion of pre-rRNAs results in chromosomal segregation disorder and mitotic catastrophe. **A** HeLa cells were treated with 5 nM Act D, 1 nM BMH-21 or 1 nM CX5461. Pre-rRNAs level was evaluated by RT-qPCR. **B** FISH was performed using a Cy3-labeled pre-rRNA probe in HeLa cells treated with 1 nM BMH-21. Chromosomes were stained by DAPI. Scale bar, 10 μm. **C** Mitotic progression was monitored and recorded under time-lapse fluorescence microscopy in HeLa-GFP-H2B+mChery-Tubulin cells treated with the indicated reagents as described in (**A**). Images were acquired every 10 min. Scale bar, 10 μm. **D** Time duration from nuclear envelope breakdown (NEBD) to mitotic exit was recorded in the cells described in (**C**). DMSO group: *n* = 72; Act D group: *n* = 72; BMH-21 group: *n* = 72; CX5461 group: *n* = 72. Data is presented as means ± S.D. **E** Indirect immunostaining was performed in HeLa cells as described in (**A**) to visualize the mitotic spindle (α-Tubulin, red). Chromosomes were stained with DAPI (left). Scale bar, 10 μm. The frequency of chromosomal misalignment, lagging, and bridge in the cells treated with DMSO, Act D, BMH-21, and CX5461 is shown (*n* > 100) (right). **F** Immunofluorescent staining was performed with anti-α-Tubulin antibody in HeLa cells treated with the indicated reagents. Scale bar, 25 μm. A quantitative comparison of multinucleated cells in HeLa cells treated with the indicated reagents is shown (*n* > 500). **G** HeLa cells were treated with 1 nM BMH-21 for 0 h, 4 h, 8 h, 12 h, 16 h, 20 h, and 24 h. Pre-rRNAs level was evaluated by RT-qPCR (left). Nascent proteins in these cells were labeled with puromycin and evaluated by Western blot using anti-puromycin antibody (left). Poncean S staining is shown as a loading control (middle). **H** HeLa cells treated with the indicated reagents were synchronized to M phase using Thymidine-Nocodazole treatment. Mitotic cells were collected by shaking off and released into fresh medium for 6 h to next G1/S phase. Apoptotic cells were determined by flow cytometry. Quantification of apoptotic cells is shown (right). **p < 0.01. ***p < 0.001. ****p < 0.0001. n.s. denotes no significance.

To determine if pre-rRNAs play in mitosis, we inhibited RNA Pol I by low dose actinomycin D (Act D), BMH-21 or CX5461 (Fig. 1A, B and S1A) and monitored the mitotic process using time-lapse image system in HeLa-GFP-H2B-mChe-Tub cells (Fig. 1C). Inhibition of pre-rRNA transcription by Act D, BMH-21 or CX5461 significantly delayed mitotic process compared with that in the control cells (187.36 ± 80.79 min in the Act D group, 252.50 ± 93.50 min in the BMH-21 group and 257.92 ± 79.50 min in the CX5461 group *versus* 123.19 ± 29.69 min in the control cells) (Fig. 1D and Videos 1–4). To determine the depletion of pre-rRNAs-caused mitotic disorders, we further examined chromosomal segregation in HeLa cells. Depletion of pre-rRNAs causes chromosomal misalignment, lagging and bridge during mitosis (Fig. 1E). As a consequence, the number of multinucleated cells increased in the cells treated with Act D, BMH-21 or CX5461, suggesting that the depletion of pre-rRNAs might cause mitotic catastrophe (Fig. 1F). To determine that the mitotic defects arise directly from the absence of pre-rRNAs rather than the downstream effects of inhibition of Pol I such as altered protein homeostasis, we performed puromycin incorporation assay to evaluate protein synthesis after cells were treated with BMH-21. When the transcription of pre-rRNA is inhibited, protein synthesis remains unaffected within 16 hours (Fig. 1G), during which time period the mitotic defects have already occurred, indicating that the absence of pre-rRNAs directly led to mitotic defects. To further confirm this phenomenon, we depleted pre-rRNAs using siRNAs specifically targeting 30S and 32S pre-rRNAs, respectively, and evaluated mitosis. We showed that depletion of 30S and 32S pre-rRNAs in the PR by siRNAs (Fig. S1B, C) directly caused mitotic defects (Fig. S1D, E). Collectively, we demonstrate that depletion of pre-rRNAs induces mitotic defects in cells.

To further determine the pre-rRNAs depletion-induced mitotic cell death, we analyzed apoptotic cells in M and next G1 phase (Fig. S2A). We found that depletion of pre-rRNAs induced an increase of apoptotic cells in the next G1 phase, while no significant difference was found in M phase (Fig. 1H), confirming that depletion of pre-rRNAs leads to mitotic catastrophe. Additionally, depletion of pre-rRNAs causes chromosomal condensation disorder leading to decreased chromosome density during mitosis (Fig. S2B) and increased the dispersion of chromosomes in metaphase (Fig. S2C). Thus, these results indicate that pre-rRNAs play a vital role in the process of chromosomal segregation and condensation during mitosis.

### Depletion of pre-rRNAs causes AURKA to approach chromosome, leading to mitotic disorder

**Fig. 2:**
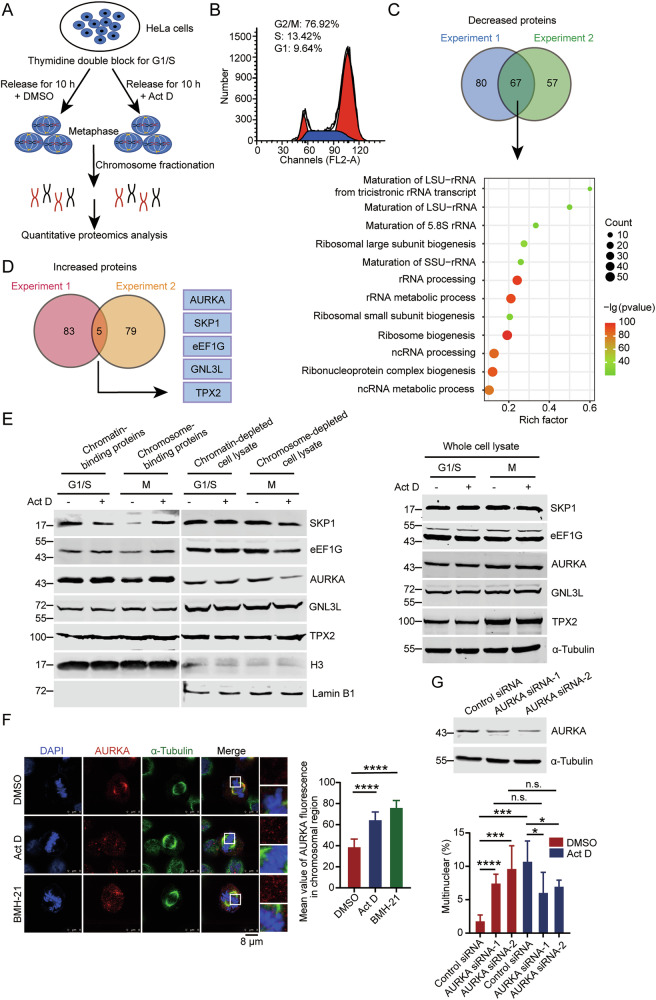
Depletion of pre-rRNAs causes AURKA to approach chromosome leading to mitotic disorder. **A** Schematic illustration of the determination of chromosome-binding proteins. HeLa cells were synchronized at G1/S phase by thymidine double blocking, and were released in the medium containing 5 nM Act D or DMSO for 10 h. Then, HeLa cells in metaphase were harvested by shaking off. Chromosome-binding proteins were isolated through chromosome fractionation, and analyzed by mass spectrometry followed by quantitative proteomics analysis. **B** Cell cycle of the synchronized cells, as described in (**A**) was determined by flow cytometry. **C** The Venn diagram (upper) shows the decreased proteins overlapped in the two independent experiments. Enriched gene sets of 67 decreased proteins are visualized by bubble plot (lower). The size of each bubble represents the count of genes enriched in the total gene set. The color represents the *P*-value for the relevant pathway. **D** The Venn diagram shows the increased proteins overlapped in the two independent experiments. **E** HeLa cells treated with 5 nM Act D or DMSO were synchronized at G1/S phase by thymidine double blocking or at M phase by release for 8 h. The chromatin-binding proteins and chromatin-depleted cell lysate at G1/S phase, and the chromosome-binding proteins and chromosome-depleted cell lysate at M phase were collected by chromosome fractionation, and subjected to Western blot using indicated antibodies. H3 and lamin B1 were used as marker of chromatin (chromosome) and chromatin (chromosome)-depleted cell lysate, respectively (left). Cells were harvested, and the whole proteins extracted from cell lysates were subjected to Western blot and probed with indicated antibodies. Alpha-Tubulin was used as a loading control (right). **F** HeLa cells treated with the indicated reagents were fixed, and indirect immunofluorescent staining was performed using anti-α-tubulin and anti-AURKA antibodies. Chromosomes were stained by DAPI. Scale bar, 8 μm (left). Quantification of mean value of AURKA fluorescence in chromosomal region in cells, as described above is shown (*n* = 15) (right). **G** HeLa cells transfected with AURKA siRNA-1, AURKA siRNA-2, or control siRNA were harvested, and Western blot was performed to evaluate AURKA protein levels. Alpha-tubulin was used as a loading control (upper). A quantitative comparison of multinucleated cells in HeLa cells treated with Act D or DMSO is shown (*n* > 500) (lower). **p < 0.01. ***p < 0.001. ****p < 0.0001. n.s. denotes no significance.

To explore the mechanism by which pre-rRNAs regulate mitosis, we used quantitative proteomics to analyze the chromosome-binding proteins when the transcription of pre-rRNA was inhibited (Fig. 2A, B). To avoid experimental variation, we have performed the experiments twice and analyzed the common changes in the repeated experiments. Venn analysis of the two independent experiments showed that 67 chromosome-binding proteins were credibly decreased (Table S1). Gene Ontology (GO) analysis of these 67 proteins indicates that most of them are significantly enriched in the maturation of rRNA processing (with a high enrichment score) and ribosome biogenesis (with the lowest *P* value) (Fig. 2C), suggesting that these proteins might localize in the PR depending on pre-rRNAs during mitosis. It is of interest that in parallel with some proteins detach from chromosomes, another group of proteins approach chromosomes when Pol I was inhibited. The Venn analysis of the chromosome-binding proteins in two independent experiments shows that the chromosome-binding of five proteins, namely SKP1, eEF1G, AURKA, GNL3L, and TPX2 significantly increased when pre-rRNAs were absent (Fig. 2D and Table S2). To further verify the binding of these five proteins with chromosome in M phase, we synchronized cells and performed chromatin (chromosome) fractionation [36]. Western blot showed that in M phase, the binding of SKP1, eEF1G, and AURKA with chromosome increased upon depleted of pre-rRNAs, while these protein levels decreased in the chromosome-depleted cell lysate. Additionally, the total amount of these proteins remained unchanged in whole cell lysate (Figs. 2E and S3A). These results suggested that AURKA, eEF1G, and SKP1, but not GNL3L and TPX2 might approach chromosome upon the depletion of pre-rRNAs. To further verify this phenomenon, we determined the cellular localization of these proteins by immunofluorescent staining (Figs. 2F and S3B). It showed that the mean value of AURKA fluorescence in chromosomal region significantly increased under the treatment of Act D or BMH-21, while that of eEF1G and SKP1 remained unchanged. It is verified by the immunofluorescent staining on the mitotic chromosome spreads (Fig. S3C).

It is known that the localization of AURKA at the spindle poles and its autophosphorylation depend on its binding to TPX2 during mitosis [37]. To determine if the change in the spatial localization of AURKA is related to TPX2, we performed immunofluorescent staining of AURKA and TPX2. We showed that TPX2 remained in the centrosome and on the spindle when AURKA was recruited to chromosomes upon depletion of pre-rRNAs, indicating that mis-localization of AURKA did not induce alterations in the localization of TPX2 (Fig. S4A). To verify the interaction between AURKA and TPX2 during mitosis with or without pre-rRNAs, we synchronized HeLa cells in M phase and performed co-immunoprecipitation. The binding of AURKA with TPX2 in mitotic cells slightly decreased when pre-rRNAs were depleted by siRNAs (Fig. S4B), indicating that a portion of AURKA dissociates from TPX2 and approaches chromosome in the absence of pre-rRNAs (Fig. S4B). In conclusion, these results confirmed that upon the depletion of pre-rRNAs, AURKA approaches chromosome in mitosis.

To determine if the binding of AURKA with chromosome is the main cause of the mitotic catastrophe caused by depletion of pre-rRNAs, we knocked down AURKA and conducted a statistical analysis on the multinucleation after inhibiting pre-rRNA transcription in HeLa cells (Fig. 2G). We found that the multinucleation caused by depletion of pre-rRNAs was significantly reversed when AURKA was depleted, suggesting that the binding of AURKA with chromosome is the main cause of mitotic disorder caused by pre-rRNAs depletion.

### AURKA selectively binds SMC2 on the chromosome upon the depletion of pre-rRNAs

**Fig. 3:**
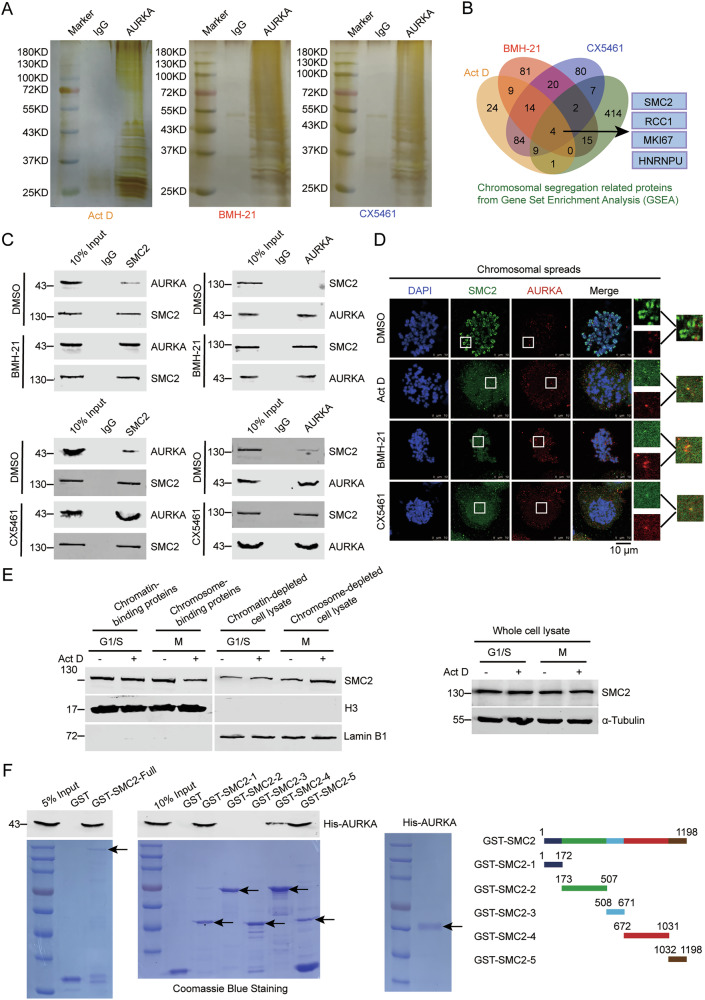
AURKA selectively binds SMC2 on the chromosome upon the depletion of pre-rRNAs. **A** HeLa cells treated with the indicated reagents were synchronized at M phase by thymidine double blocking and harvested by shaking off. Chromosome-binding proteins were immunoprecipitated with anti-AURKA antibody. The AURKA-binding proteins were resolved by SDS-PAGE and silver staining. **B** Venn diagram of 4 datasets (“Act D”, “BMH-21”, “CX5461”, and “Chromosomal segregation related proteins from GSEA”) is shown. Four proteins are shared (right). **C** HeLa cells treated with the indicated reagents were synchronized and harvested as described in (**A**). Immunoprecipitation was performed on the chromosome-binding proteins using indicated antibodies. The immunoprecipitates were immunoblotted with the indicated antibodies. **D** Immunofluorescent staining on the mitotic chromosome spreads was performed in HeLa cells treated with the indicated reagents using anti-SMC2 and anti-AURKA antibodies. Chromosomes were stained with DAPI. Scale bar, 10 μm. **E** HeLa cells treated with Act D or DMSO were synchronized at G1/S phase by thymidine double blocking or at M phase by release for 8 h. The chromatin-binding proteins and chromatin-depleted cell lysate at G1/S phase, and the chromosome-binding proteins and chromosome-depleted cell lysate at M phase were collected by chromosome fractionation, and subjected to Western blot using indicated antibodies. H3 and lamin B1 were used as marker of chromatin (chromosome) and chromatin (chromosome)-depleted cell lysate, respectively (left). Cells were harvested, and the proteins extracted from whole cell lysates were subjected to Western blot and probed with indicated antibodies. Alpha-Tubulin was used as a loading control (right). **F** The schematic diagram represents the constructs of GST-SMC2 deletion mutants (right). The purified GST protein, His-AURKA, and GST-SMC2 and its deletion mutants were resolved by SDS-PAGE and stained by coomassie blue staining. GST pull-down assay was performed using purified GST-SMC2 and its deletion mutants and His-AURKA (left).

To unravel how AURKA acts when it approaches chromosome, we synchronized cells in the metaphase, isolated chromosome and performed immunoprecipitation experiments with anti-AURKA antibody when pre-rRNA transcription was inhibited by Act D, BMH-21 or CX5461, and the AURKA-binding proteins on chromosome were determined by mass spectrometry (Fig. 3A and Table S3). When the transcription of pre-rRNA was inhibited with various reagents, 18 proteins were commonly found to be bound by AURKA (Fig. 3B). To find out the key proteins controlling chromosomal segregation and condensation in these AURKA-binding proteins, we applied Venn analysis to compare the AURKA-binding proteins with the proteins involved in chromosomal segregation from Gene Set Enrichment Analysis (GSEA) (GOBP_CHROMOSOME_SEGREGATION). Four proteins were selectively found in these protein databases (Fig. 3B), among which SMC2 is the most crucial protein for chromosomal segregation and condensation. To verify the interaction between AURKA and SMC2 during mitosis with or without pre-rRNAs, we synchronized HeLa cells by thymidine double blocking, shook off cells in M phase, and performed co-immunoprecipitation. The binding of AURKA with SMC2 in mitotic cells was dramatically enhanced when the transcription of pre-rRNA was inhibited (Fig. 3C). To confirm this result, we performed immunofluorescent staining of SMC2 and AURKA. We showed that AURKA invaded chromosomal region, SMC2 is no longer located on chromosome but diffused in the cytoplasm and co-localized with AURKA upon the depletion of pre-rRNAs (Fig. 3D). To further verify this phenomenon, we treated cells with low dose Act D and isolated chromosomes to evaluate chromosomal binding of SMC2 and AURKA by Western blot. The binding of SMC2 with chromosomes dramatically decreased in M phase when pre-rRNAs were absent, while the level of SMC2 increased in the chromosome-depleted cell lysates. Additionally, the total amount of SMC2 remained unchanged in the whole cell lysates (Fig. 3E). Further, to determine if AURKA directly binds SMC2, we performed in vitro GST pull-down experiments using purified His-AURKA and GST-SMC2 (Fig. 3F). The result shows that His-AURKA directly binds to GST-SMC2. To map the AURKA-binding domain in SMC2, GST pull-down experiments were performed with purified GST-SMC2 deletion mutants and His-AURKA protein. We divided SMC2 into 5 deletion mutants according to its functional domain [15], including SMC2-1 (N-terminus ATPase head), SMC2-2 (coiled-coil arm), SMC2-3 (SMC Hinge), SMC2-4 (coiled-coil arm) and SMC2-5 (C-terminus ATPase head) (Fig. 3F). The result shows that AURKA directly binds to SMC2-1, SMC2-4 and SMC2-5, which mainly contains the ATPase head of SMC2 (Fig. 3F). These results demonstrate that depletion of pre-rRNAs enhances the binding of AURKA with SMC2 on the chromosome during mitosis.

### AURKA-mediated phosphorylation of SMC2 T574 leads to mitotic catastrophe

**Fig. 4:**
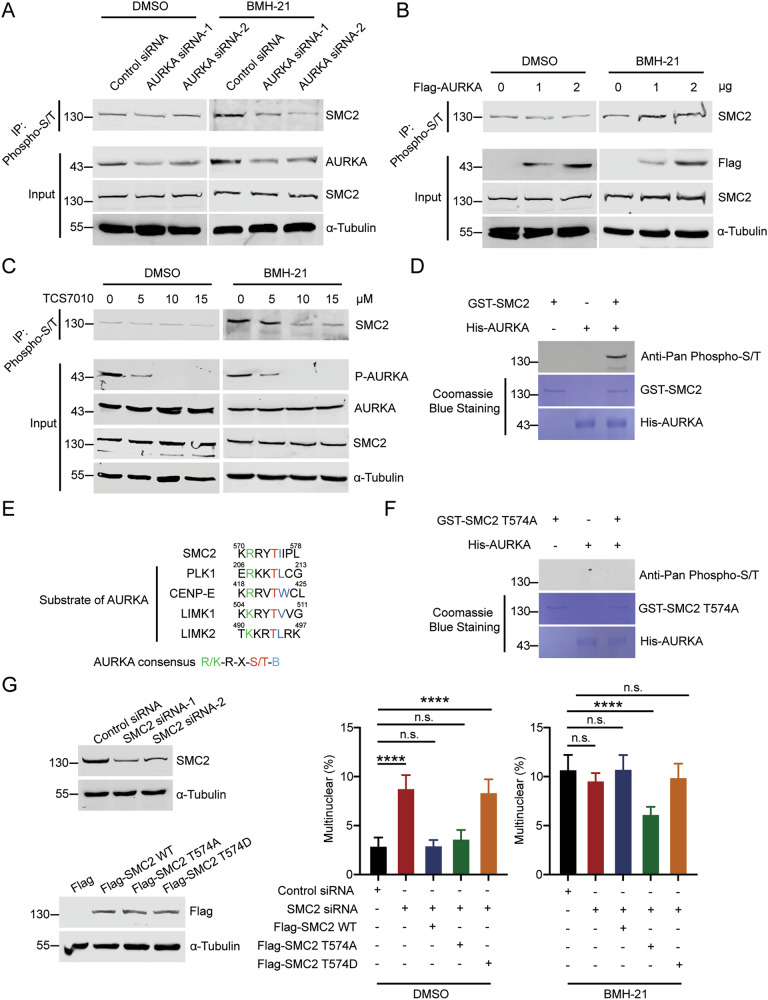
AURKA-mediated phosphorylation of SMC2 T574 leads to mitotic catastrophe. **A** HeLa cells were transfected with AURKA siRNA-1, AURKA siRNA-2, or control siRNA. These cells were treated with BMH-21 or DMSO, and were synchronized at M phase by nocodazole and harvested by shaking off. Immunoprecipitation was performed with anti-pan-phospho-serine/threonine antibody. The immunoprecipitates were immunoblotted with the indicated antibodies. **B** HeLa cells were transfected with the indicated doses of Flag-AURKA plasmid or Flag-vector. These cells were treated with BMH-21 or DMSO, and were synchronized at M phase and harvested as described in (**A**). Immunoprecipitation was performed with anti-pan-phospho-serine/threonine antibody. The immunoprecipitates were immunoblotted with the indicated antibodies. **C** HeLa cells were treated with 0 μM, 5 μM, 10 μM, and 15 μM TCS7010. These cells were treated with BMH-21 or DMSO, and were synchronized at M phase and harvested as described in (**A**). Immunoprecipitation was performed with anti-pan-phospho-serine/threonine antibody. The immunoprecipitates were immunoblotted with the indicated antibodies. **D** In vitro phosphorylation experiment was performed using purified His-AURKA and GST-SMC2. Proteins were resolved by SDS-PAGE and probed with anti-pan-phospho-lysine antibody. Coomassie blue staining showed the purified proteins. **E** Sequence alignment of the AURKA phosphorylation consensus within SMC2 and substrates of AURKA. Phosphorylated threonine residues are highlighted in red, arginine at the *n* − 3 position is highlighted in green, and hydrophobic residues at the *n* + 1 position are highlighted in blue. **F** In vitro phosphorylation experiment was performed using purified His-AURKA and GST-SMC2 T574A. Proteins were resolved by SDS-PAGE and probed with the indicated antibodies. Coomassie blue staining showed the purified proteins. **G** HeLa cells were transfected with the indicated siRNAs and the indicated plasmids (left). A quantitative comparison of multinucleated cells in HeLa cells in the indicated circumstances is shown (*n* > 500) (right). **p < 0.01. ***p < 0.001. ****p < 0.0001. n.s. denotes no significance.

As AURKA is a serine/threonine kinase, we next sought to determine if AURKA phosphorylates SMC2 during mitosis. We evaluated phosphorylation of SMC2 by immunoprecipitation with anti-pan-phospho-serine/threonine antibody in mitotic cells. When the transcription of pre-rRNA is inhibited, the phosphorylation level of SMC2 increased (Fig. 4A), indicating that inhibition of pre-rRNA transcription causes elevation of SMC2 phosphorylation level. To determine if the SMC2-phosphorylation is mediated by AURKA, we silenced AURKA and showed that the phosphorylation level of SMC2 decreased in AURKA-depleted cells (Fig. 4A). Additionally, phosphorylation level of SMC2 increased upon ectopic expression of Flag-AURKA, while the protein level of SMC2 was not affected (Fig. 4B). However, under normal conditions, the phosphorylation level of SMC2 stayed unchanged in AURKA-depleted cells (Fig. 4A) or upon ectopic expression of Flag-AURKA (Fig. 4B). These results reveal that AURKA phosphorylates SMC2 only upon the depletion of pre-rRNAs during mitosis. We further treated cells with TCS7010, an exceptionally selective AURKA inhibitor. Upon the depletion of pre-rRNAs, the phosphorylation level of SMC2 decreased notably when the enzyme activity of AURKA was inhibited, while the protein level of SMC2 was not affected (Fig. 4C). These results illustrate that AURKA phosphorylates SMC2 in cells without affecting the expression level of SMC2. To confirm the direct phosphorylation of SMC2 by AURKA, we performed in vitro phosphorylation experiments using purified GST-SMC2 and His-AURKA. AURKA phosphorylates SMC2 in vitro (Fig. 4D).

Thereafter, the potential phosphorylated sites of SMC by AURKA were determined. It is known that AURKA phosphorylates the substrates through recognizing consensus R/K/N-R-X-S/T-B, where B denotes any hydrophobic residue with the exception of Pro [38]. A hydrophobic residue at position *n* + 1 is a specificity determinant almost as powerful as the Arg-Arg doublet at positions n-2/n-3 [39]. Therefore, we aligned the amino acid sequence of SMC2 with several known substrates of AURKA. We found that there was only one potential residue, T574 of SMC2, that reaches the standard of AURKA substrate among the 95 possible phosphorylation sites analyzed using the Netphos3.1a server (Figs. 4E and S5A). Additionally, the motif containing T574 in SMC2 was highly evolutionarily conserved in various species (Fig. S5B). Further, we performed in vitro phosphorylation experiments using purified GST-SMC2 T574A (non-phosphorylatable mutant) and His-AURKA. The result showed that AURKA cannot phosphorylate SMC2 T574A in vitro (Fig. 4F), indicating that the specific phosphorylation site of SMC2 by AURKA is T574. To determine if the phosphorylation of SMC2 T574 affects mitosis, we generated Flag-SMC2 T574A and Flag-SMC2 T574D (phosphorylation-mimicking mutant) plasmids. Then we ectopically expressed Flag-SMC2 WT, Flag-SMC2 T574A, and Flag-SMC2 T574D in SMC2-depleted cells, respectively. Under normal circumstances, the proportion of multinucleated cells increased when SMC2 was knocked down by siRNA (Figs. 4G and S5C), indicating that SMC2 is required for normal mitosis as previously described [40]. Flag-SMC2 WT or Flag-SMC2 T574A restored the depletion of SMC2-caused multinucleated cells, but Flag-SMC2 T574D failed to do so (Fig. 4G), indicating that the phosphorylation of T574 disrupts normal mitosis. Afterwards, we determined if the phosphorylation of T574 affects mitosis when the transcription of pre-rRNA is inhibited. Upon the depletion of pre-rRNAs, only the phosphorylation deficient mutant Flag-SMC2 T574A, rather than Flag-SMC2 WT and Flag-SMC2 T574D, rescues the multinuclear cells in SMC2-depleted cells (Fig. 4G). These results confirm that phosphorylation of SMC2 T574 disrupts its normal function in controlling mitosis. In conclusion, AURKA phosphorylates SMC2 at T574 when pre-rRNA transcription is inhibited, which leads to mitotic catastrophe.

### Endogenous SMC2 T574 is phosphorylated by AURKA in mitosis in pre-rRNAs-depleted cells

**Fig. 5:**
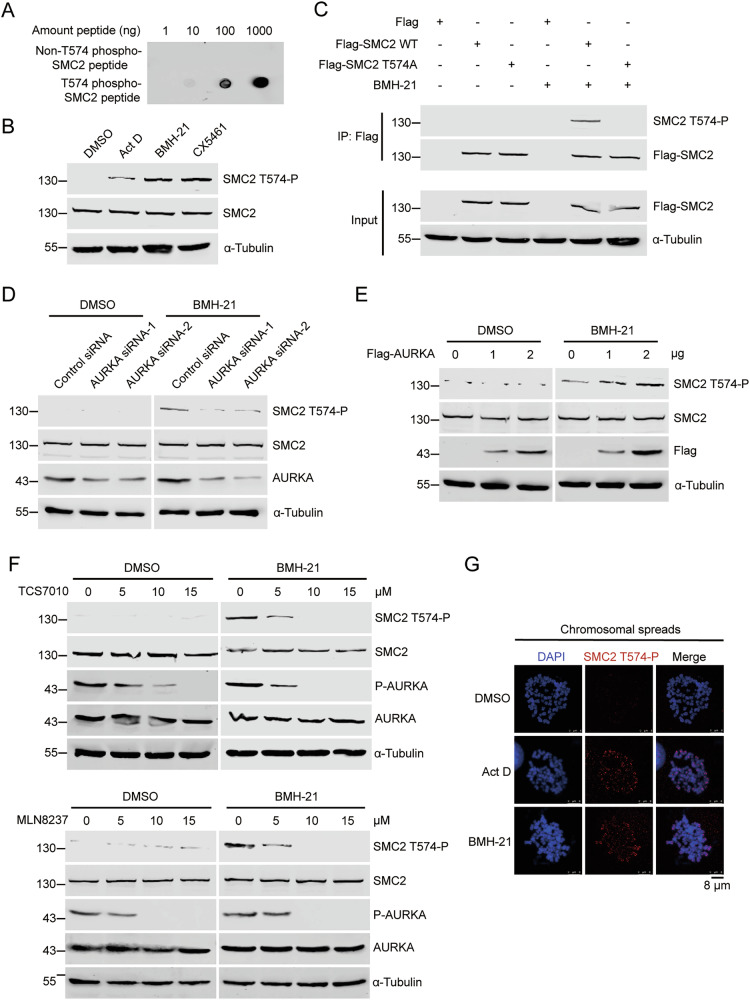
Endogenous SMC2 T574 is phosphorylated by AURKA in mitosis in pre-rRNAs-depleted cells. **A** Dot blotting was performed with non-T574 phospho-SMC2 peptide or T574 phospho-SMC2 peptide to detect the specificity of anti-SMC2 T574-P antibody. **B** HeLa cells were treated with the indicated reagents. These cells were synchronized at M phase by thymidine double blocking and harvested by shaking off. Proteins were resolved by SDS-PAGE and probed with the indicated antibodies. Alpha-tubulin was used as a loading control. **C** HeLa cells were transfected with the indicated plasmids and treated with BMH-21 or not. These cells were synchronized at M phase by nocodazole and harvested by shaking off. Co-immunoprecipitation was performed with anti-Flag antibody, and the phosphorylation levels of Flag-SMC2 were evaluated by western blot using anti-SMC2 T574-P antibody. Alpha-tubulin was used as a loading control. **D** HeLa cells were transfected with the indicated siRNAs and treated with BMH-21 or not. These cells were synchronized and harvested as described in (**C**). Proteins were resolved by SDS-PAGE and probed with the indicated antibodies. Alpha-tubulin was used as a loading control. **E** HeLa cells were transfected with the indicated doses of Flag-AURKA or Flag vector and treated with BMH-21 or not. These cells were synchronized and harvested as described in (**C**). Proteins were resolved by SDS-PAGE and probed with the indicated antibodies. Alpha-tubulin was used as a loading control. **F** HeLa cells were treated with the indicated doses of TCS7010 or MLN8237, and treated with BMH-21 or not. These cells were synchronized and harvested as described in (**C**). Proteins were resolved by SDS-PAGE and probed with the indicated antibodies. Alpha-tubulin was used as a loading control. **G** HeLa cells were treated with Act D, BMH-21, or DMSO. These cells were fixed, and indirect immunofluorescent staining was performed using anti-SMC2 T574-P antibody. Chromosomes were stained by DAPI. Scale bar, 8 μm.

To validate the existence of the endogenous phosphorylation of SMC2 at T574 in cells, we generated SMC2 T574 phosphorylation specific antibody (designated as “SMC2 T574-P”) to recognize the SMC2 T574 phosphorylation. The dot blotting showed that SMC2 T574-P antibody specifically recognizes T574 phosphorylated-SMC2 peptide (Fig. 5A). When the transcription of pre-rRNA was inhibited, the phosphorylation level of SMC2 T574 increased in mitotic cells (Fig. 5B), confirming that the endogenous SMC2 T574 is phosphorylated in the absence of pre-rRNAs. The SMC2 T574-P antibody detected the phosphorylated SMC2 band in the immunoprecipitated Flag-SMC2 WT but not flag-SMC2 T574A, and the level of SMC2 T574-P increased upon BMH-21 treatment (Fig. 5C). Further, when the transcription of pre-rRNA is inhibited, the level of SMC2 T574-P decreased in AURKA-depleted cells (Fig. 5D) and increased upon ectopic expression of Flag-AURKA (Fig. 5E), while the protein level of SMC2 was not affected. However, under normal conditions, the level of SMC2 T574-P was hardly detectable and stayed unchanged in AURKA-depleted cells (Fig. 5D) or upon ectopic expression of Flag-AURKA (Fig. 5E), demonstrating that endogenous SMC2 T574 is phosphorylated by AURKA specifically when pre-rRNA transcription is inhibited. We then treated cells with selective inhibitors of AURKA, TCS7010, or MLN8237 and determined the level of SMC2 T574-P. We found that upon the depletion of pre-rRNAs, the level of SMC2 T574-P diminished when the catalytic activity of AURKA was inhibited, while the protein level of SMC2 was not affected (Fig. 5F), further confirming that SMC2 T574 is phosphorylated by AURKA upon the depletion of pre-rRNAs. Immunofluorescent staining showed that SMC2 T574-P located on chromosome upon depletion of pre-rRNAs in mitosis (Fig. 5G). Together, these results demonstrate that AURKA phosphorylates SMC2 at T574 endogenously in pre-rRNAs-depleted cells during mitosis.

### SMC2 T574-P disrupts SMC2/SMC4 binding and the binding of SMC2/SMC4 with chromosomal DNA

**Fig. 6:**
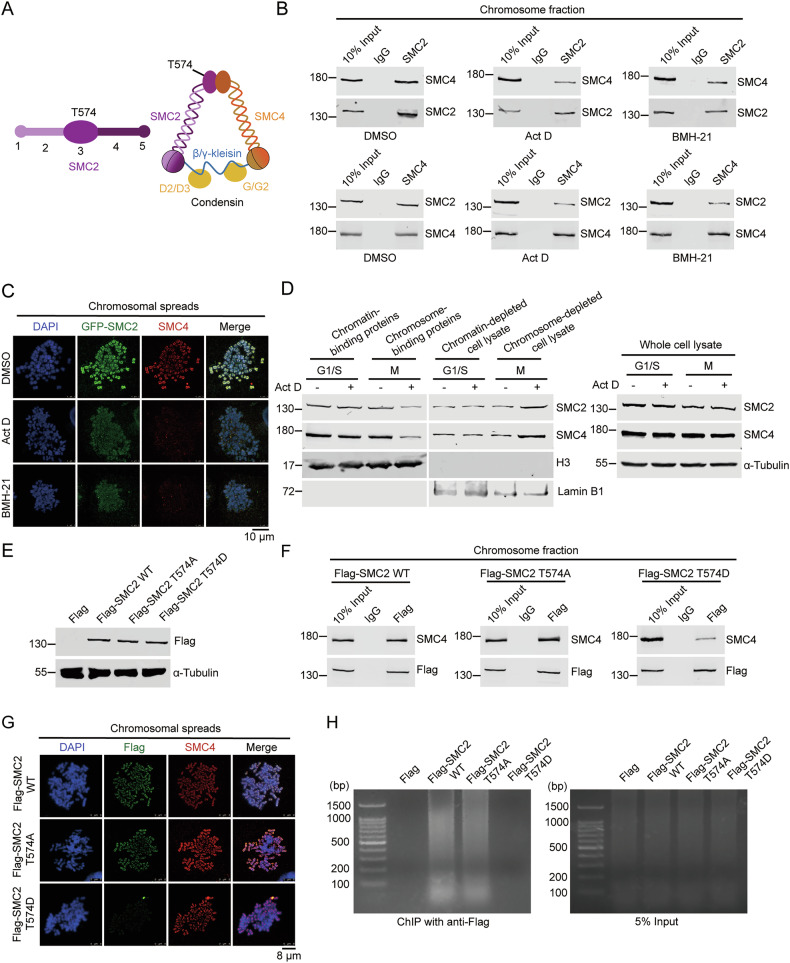
SMC2 T574-P disrupts SMC2/SMC4 binding and the binding of Condensin with chromosomal DNA. **A** A schematic of SMC2 and Condensin. The location of T574 is marked in the diagram. **B** HeLa cells treated with the indicated reagents were synchronized at M phase by thymidine double blocking and harvested by shaking off. Immunoprecipitation was performed with the chromosome-binding proteins using anti-SMC2 and anti-SMC4 antibodies. The immunoprecipitates were immunoblotted with the indicated antibodies. **C** HeLa cells were transfected with GFP-SMC2 plasmid and treated with the indicated reagents. Immunofluorescent staining on the mitotic chromosome spreads was performed in these cells using anti-SMC4 antibody. Chromosomes were stained with DAPI. Scale bar, 10 μm. **D** HeLa cells treated with 5 nM Act D or DMSO were synchronized in G1/S phase by thymidine double blocking and released to M phase. Chromosome fractionation was performed to obtain chromatin (or chromosome)-binding proteins and chromatin (or chromosome)-depleted cell lysate at G1/S phase and M phase, respectively. The fractions were subjected to Western blot using indicated antibodies. H3 and lamin B1 were used as the markers for chromatin (or chromosome) and chromatin (or chromosome)-depleted cell lysate, respectively. Cells were harvested, and the proteins extracted from whole cell lysates were subjected to Western blot and probed with indicated antibodies. Alpha-Tubulin was used as a loading control (right). **E** HeLa cells were transfected with the indicated plasmids for 24 h. Cell lysates were extracted, and proteins were subjected to Western blot probed with the indicated antibodies. Alpha-Tubulin was used as a loading control. **F** HeLa cells transfected with the indicated plasmids were synchronized at M phase by nocodazole and harvested by shaking off. Immunoprecipitation was performed on the chromosome-binding proteins using anti-Flag antibody. The immunoprecipitates were immunoblotted with the indicated antibodies. **G** HeLa cells were transfected with the indicated plasmids. Immunofluorescent staining on the mitotic chromosome spreads was performed in these cells using anti-Flag and anti-SMC4 antibodies. Chromosomes were stained with DAPI. Scale bar, 8 μm. **H** HeLa cells transfected with the indicated plasmids were synchronized and harvested as described in (**F**). ChIP was performed with anti-Flag antibody and the immunoprecipitates were resolved by Agarose gel. **G** A working model explaining the mechanism by which pre-rRNAs control mitosis by maintaining chromosomal segregation and condensation through protecting SMC2 from AURKA-mediated phosphorylation.

It is known that the complete function of Condensin requires the correct binding of SMC2 with SMC4 [13]. SMC2 binds to SMC4 mainly via its hinge domain, where T574 locates (Figs. 3F and 6A). To this end, we speculated that phosphorylation of SMC2 T574 might disrupt SMC2/SMC4 binding to disrupt the function of Condensin. To address this issue, we performed co-immunoprecipitation in chromosome fraction to evaluate SMC2/SMC4 binding in HeLa cells. The binding of SMC2 with SMC4 was dramatically decreased on the chromosomes when the transcription of pre-rRNA was inhibited (Fig. 6B). To verify this result, we expressed GFP-SMC2 in HeLa cells and performed immunofluorescent staining to determine the localization of SMC4. We showed that both GFP-SMC2 and SMC4 locate on the chromosome under normal conditions, while they no longer locate on the chromosome and lose co-localization with each other upon the depletion of pre-rRNAs (Fig. 6C), suggesting that the presence of pre-rRNAs is required for the chromosomal localization of SMC2 and SMC4. To further verify this phenomenon, we treated cells with low dose Act D and isolated chromosome to evaluate chromosomal binding of SMC2 and SMC4 by Western blot. The binding of SMC2 and SMC4 with chromosome dramatically decreased in M phase when pre-rRNAs were absent, while the level of SMC2 and SMC4 increased in the chromosome-depleted cell lysate. The total amount of SMC2 and SMC4 remained unchanged in the whole cell lysate (Fig. 6D). Together, these results demonstrate that pre-rRNAs are required for the binding of SMC2 and SMC4 with chromosome. To determine if the phosphorylation of T574 affects the binding of SMC2 with SMC4, we ectopically expressed Flag-SMC2 WT, Flag-SMC2 T574A, or Flag-SMC2 T574D in HeLa cells (Fig. 6E) and performed immunoprecipitation with chromosome fraction. The results showed that on the chromosomes, the binding of Flag-SMC2 T574D with SMC4 significantly weakened, while the binding of Flag-SMC2 T574A with SMC4 slightly increased when compared with Flag-SMC2 WT (Fig. 6F). Correspondently, this result was confirmed by immunofluorescent staining on the metaphase chromosomal spread (Fig. 6G). Previous studies have shown that point mutations in the hinge domain that interfere with SMC2/SMC4 dimerization significantly reduce its binding with chromosomal DNA [15, 41]. To determine if pre-rRNAs affect the binding of SMC2/SMC4 to chromosomal DNA, we performed chromatin immunoprecipitation (ChIP) as described previously [42] in cells treated with Act D, BMH-21, or DMSO. In alignment with our conjecture, when the transcription of pre-rRNA is inhibited, the binding of SMC2 to chromosomal DNA is weakened (Fig. S6A). To confirm if AURKA affects the binding of SMC2/SMC4 to DNA through phosphorylating SMC2, we performed ChIP in cells treated with TCS7010. Upon depletion of pre-rRNAs, the binding of SMC2 to chromosomal DNA was weakened (Fig. S6B). However, as the kinase activity of AURKA was inhibited by TCS7010, the binding of SMC2 to DNA gradually recovered (Fig. S6B), confirming that AURKA-mediated phosphorylation of SMC2 disrupts the binding of SMC2 with chromosomal DNA. Further, to confirm if the phosphorylation of SMC2 T574 interferes with the binding of SMC2/SMC4 to chromosomal DNA, we performed ChIP in cells ectopically expressed Flag-SMC2 WT, Flag-SMC2 T574A, or Flag-SMC2 T574D. The result showed that the binding of Flag-SMC2 T574D to chromosomal DNA was significantly weakened compared with Flag-SMC2 WT and Flag-SMC2 T574A (Fig. 6H). These results demonstrate that SMC2 T574-P by AURKA disrupts SMC2/SMC4 binding and the binding of SMC2/SMC4 with chromosomal DNA, leading to mitotic catastrophe.

## Discussion

Although nucleolar rRNA synthesis ceases and nucleolar structure disappears when cells enter mitosis, the amount of pre-rRNAs keeps unchanged as in interphase [43]. Additionally, pre-rRNAs have been found to localize in the PR, associate with condensed chromosomes by interacting with Ki-67, and promoted chromosomal dispersion during mitosis [18, 27, 44]. However, the specific regulatory function and mechanism of pre-rRNAs in mitosis remain largely elusive. In the present study, we observed the abnormal phenomena of mitosis when the transcription of pre-rRNA was inhibited. The completion time of mitosis is prolonged, and chromosomal misalignment, lagging, and bridge significantly increase upon the depletion of pre-rRNAs. Accompanying with these phenomena, is chromosomal condensation defect. These findings suggest that pre-rRNAs are essential for maintaining normal mitosis.

As PR composes of 30-47% of chromosomal volume [21], we asked if pre-rRNAs, as the main PR components, could act as a guardian to protect the inner chromosomal scaffold from invasions by non-chromosomal proteins. To verify this anticipation, we firstly utilized quantitative proteomics to analyze the changes in chromosome-binding proteins upon deletion of pre-rRNAs. Interestingly, we found that when pre-rRNA transcription is inhibited, AURKA approaches the chromosomes during mitosis. AURKA belongs to the serine/threonine kinase families and is essential for the cell cycle control in eukaryotes [45]. Under normal circumstances, AURKA is mainly located in the centrosomes and also spreads on the spindle microtubules in metaphase, while its mis-localization might induce mitotic disorders [46, 47]. In present study, upon the depletion of pre-rRNAs, AURKA approaches chromosome, while the localization of AURKA on the spindle microtubules is reduced, indicating that the pre-rRNA depletion-induced mitotic catastrophe might due to the mis-localization of AURKA, and pre-rRNAs act as a barrier for protecting chromosomes from invasion by AURKA.

To explore the mechanism for AURKA to interfere with mitosis under pre-rRNA depletion, we determined the proteins that AURKA binds with on the chromosomes by immunoprecipitation and proteomics analysis. The results show that when pre-rRNA transcription is inhibited, AURKA binds to SMC2, which is an essential component of Condensin complex. Condensin is a major player in chromosome dynamics in mitotic and meiotic cells through regulating chromosome condensation and segregation [5, 48]. Inhibition of Condensin complex function causes abnormal chromosomal condensation and segregation [10]. Therefore, we speculated that when pre-rRNA transcription is inhibited, the binding of AURKA with SMC2 might interfere with the Condensin function, ultimately resulting in mitotic catastrophe. Through in-cell and in vitro phosphorylation experiments, we found that AURKA phosphorylates SMC2. Proteomics analyses combined with Condensin-specific identification of phosphorylation sites indicate that Condensin is a heavily phosphorylated enzyme in both yeast and humans [49]. Previous studies have shown that four of five Condensin subunits (SMC4, hCAP-D2, hCAP-G, and hCAP-H) are phosphorylated in both interphase and M phase, and there was little difference in the overall phosphorylated levels of these four proteins in interphase and M phase [50]. However, SMC2 appears to be the “phospho-orphan” of the complex with no phospho-site reported so far in the human subunit and only one phosphorylation site in the yeast subunit [50, 51]. In the present study, we found for the first time that SMC2 is phosphorylated by AURKA during mitosis when pre-rRNAs are absent.

Next, we further clarified the phosphorylation site of SMC2 by AURKA. SMC2 T574 is phosphorylated by AURKA in cell only when pre-rRNA transcription is inhibited. T574 is precisely located in the hinge region of SMC2, which is a crucial interaction domain between SMC2 and SMC4 [14]. Through co-immunoprecipitation and immunofluorescence, we showed that the binding of SMC2/SMC4 is significantly weakened by phosphorylation of SMC2 T574. As the dimerization of SMC2/SMC4 is required for the DNA binding of Condensin [52], the phosphorylation of SMC2 T574 might affect the binding of SMC2/SMC4 to chromosomal DNA. Ultimately, we validated this hypothesis using ChIP. Here, we have identified T574 as a new phosphorylation site on SMC2. The phosphorylation of SMC2 T574 disrupts the function of the Condensin complex, leading to mitotic catastrophe.

It is known that AURKA locates in the centrosome and on the spindle during mitosis. In the present study, we found that AURKA approaches chromosomes upon depletion of pre-rRNAs. To determine if the mis-localization of AURKA is due to the defects in centrosome or spindle assembly, we evaluated centrosome and spindle assembly by immunofluorescent staining of γ-tubulin and α-tubulin, respectively, after transient depletion of pre-rRNAs using siRNAs targeting 30S and 32S pre-rRNAs. The results showed that γ-tubulin and the colocalization of AURKA with *γ*-tubulin remained almost unchanged, indicating that the mislocalization of AURKA in mitosis is not caused by defects in centrosome assembly. In the meantime, we found that the signal of AURKA on the spindle significantly decreased while that on the chromosomes increased (Fig. S7A). Additionally, we showed that the AURKA departed from α-tubulin and approached chromosomes when pre-rRNA was depleted, while *α*-tubulin signal also slightly decreased (Fig. S7B). These results suggest that depletion of pre-rRNAs causes the recruitment of AURKA to chromosomes and induces defects in spindle assembly simultaneously. If the mis-localization of AURKA is caused by the defects in spindle assembly requires further exploration.

Since AURKA and AURKB share the similarity in sequence, structure, and phosphorylation motif of their substrates [53], we wanted to know if SMC2 T574 could be phosphorylated by AURKB during mitosis when pre-rRNAs are absent. Immunoprecipitation showed that AURKB didn’t bind to SMC2 during mitosis regardless of whether pre-rRNAs were absent or not (Fig. S8A). To explore if AURKB phosphorylates SMC2, we treated cells with barasertib (a selective inhibitor of AURKB) and evaluated SMC2 T574-P. Upon the depletion of pre-rRNAs, the level of SMC2 T574-P stayed unchanged when the kinase activity of AURKB was inhibited during G1/S phase or M phase (Fig. S8B), demonstrating that the phosphorylation of SMC2 T574 is not related to AURKB. The localization of AURKA at the spindle poles and its autophosphorylation depend on its binding to TPX2 during mitosis under normal conditions [37], while we found that a portion of AURKA dissociated from TPX2 and approached chromosomes in the absence of pre-rRNAs (Fig. S4A). As SMC2 possesses the consensus sequence of AURKA’s substrates, we thus speculate that when pre-rRNAs were depleted from the PR, AURKA might approach chromosomes due to the exist of the consensus sequence of its substrates in SMC2 and other potential substrates such as Ki67. However, this speculation needs further validation.

Of note, SMC2 has been found to be over-expressed in a significant number of patients with colorectal cancer, gastric cancer, lymphoma, and some types of neuroblastoma [54]. In addition, when SMC2 expression is knocked down, tumor growth is significantly reduced in a mouse model of colorectal cancer [55]. Delivering specific antibody against the SMC2 intracellularly is an effective strategy, which aims to reduce cancer malignancy by targeting cancer stem cells (CSC), the tumoral subpopulation responsible of tumor recurrence and metastasis [56]. As the phosphorylation of SMC2 T574 abolished the function of Condensin in mitosis by interfering the SMC2/SMC4 binding, the molecules mimicking the T574 phosphorylation might provide a new potential strategy for tumor therapy.

Act D has been used in combination with chemotherapy or multimodality treatment for some cancers, such as Wilms’ tumor, childhood rhabdomyosarcoma, and Ewing’s sarcoma [57]. Similarly, CX5461 has been used for early-stage clinical studies of BRCA1-, BRCA2-, and PALB2-mutated cancers [58]. Additionally, BMH-21 also exerts potent anti-tumorigenic effects in mammalian cell lines, ex vivo tissues, and mouse models [59]. These drugs have been anticipated to inhibit tumor growth by suppressing the transcription of pre-rRNA to decrease ribosomal biogenesis. Here, we found that the absence of pre-rRNAs causes the phosphorylation of SMC2 in mitosis, leading to mitotic catastrophe. Therefore, the phosphorylation of SMC2 might be an unknown mechanism for the Pol I inhibitors to treat tumors, indicating that the mutations of SMC2 T574 might reduce the sensitivity of Pol I inhibitors in cancer treatment. However, if there is gene mutation coding SMC2 T574 needs further validation.

In the present study, we also found that when pre-rRNAs are absent, more than 67 proteins detach from the chromosomes in mitosis. These proteins are mainly responsible for the rRNA processing and ribosomal biogenesis, and might play in mitosis through binding to pre-rRNAs. Among these proteins, TopBP1 was found to be recruited to DNA lesions by pre-rRNA, to form TopBP1 foci via liquid–liquid phase separation (LLPS) in response to DNA damage [60]. Therefore, the interaction between pre-rRNA and TopBP1 might play a role in mitosis. Our previous research also revealed that U3 snoRNA controls the PR distribution of DDX21 by LLPS in mitosis. When pre-rRNA transcription was inhibited, DDX21 dispersed in the cytoplasm [26]. DDX21 is also found in these 67 proteins. We thus presume that these 67 or even more proteins might control mitosis through regulating the LLPS in the PR or other mechanisms.

It has been known that on mitotic chromosomes, pre-rRNAs are localized to the PR mainly by interacting with the chromosomal binding protein Ki-67 [18] and are involved in the regulation of chromosomal dispersion with Ki-67 through electrostatic interactions [61]. Depletion of pre-rRNAs leads to abnormal chromosomal clustering [61], which was also confirmed in our result (Fig. 3D). In the present study, when pre-rRNA transcription is inhibited, AURKA approaches chromosome to phosphorylate SMC2, which is a crucial regulator of inner chromosomal scaffold. Besides SMC2, Ki-67 was also found in the AURKA-binding proteins in the absence of pre-rRNAs (Fig. 3B). Phosphorylation plays a pivotal role in Ki-67 phase separation by charge blockiness on mitotic chromosome [62]. Then we aligned the amino acid sequence of Ki-67 with the AURKA phosphorylation consensus and found that Ki-67 possesses a consensus of AURKA substrate (Fig. S9). Therefore, if Ki-67 is phosphorylated by AURKA during mitosis in the absence of pre-rRNAs needs further exploration. Collectively, we have provided evidence for the protection of inner chromosomal scaffold by the PR component pre-rRNAs.

It is known that the decrease of pre-rRNA level is associated with cellular senescence in mouse and human due to the higher methylation level of rDNA [63]. Additionally, some developmental diseases such as acrofacial dysostosis (Cincinnati type) and hypomyelinating leukodystrophy (HL) have been found to be related with lower Pol I activity caused by gene mutations coding Pol I subunit [64]. In return, the decrease of pre-rRNA levels might accelerate cell senescence [65]. In the present study, we found that the inhibition of pre-rRNA transcription caused mitotic catastrophe leading to apoptosis, which might function as a self-protection mechanism of cell population through eliminating aging cells and Pol I gene mutated cells. Thereafter, we wanted to know if endogenous SMC2 T574-P exists under normal conditions or stress conditions. We performed immunofluorescence aiming at elucidating the significance of the AURKA-mediated SMC2 T574-P in interphase. SMC2 T574-P was detected in interphase, and co-localized with AURKA (Fig. S10A), indicating that SMC2 T574 is phosphorylated by AURKA under normal conditions. Then we treated cells with 100 μM H_2_O_2_ for cellular oxidative stress and 500 J/m^2^ UV for DNA damage in G1/S phase, G2 phase, or M phase, respectively. The level of SMC2 T574-P increased when cells were under oxidative stress or DNA damage (Fig. S10A, B). These results indicate that, in addition to pre-rRNA depletion, intracellular stress responses can also slightly trigger the phosphorylation of SMC2 T574, suggesting that the phosphorylation of SMC2 T574 might be related to stress responses. However, this requires further research.

**Fig. 7:**
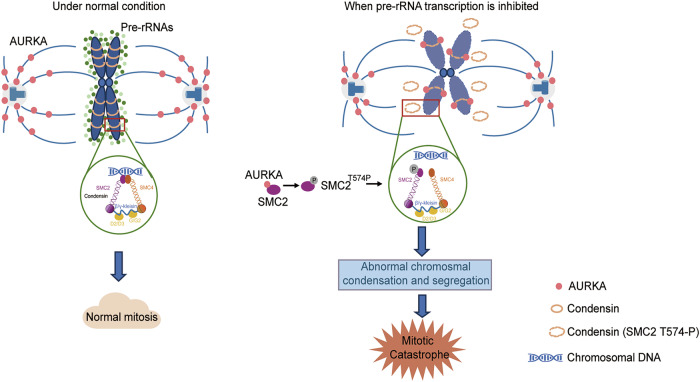
A working model explaining the mechanism by which pre-rRNAs control mitosis by maintaining chromosomal segregation and condensation through protecting SMC2 from AURKA-mediated phosphorylation.

In conclusion, we demonstrate that the depletion of pre-rRNAs allows AURKA to approach chromosomes during mitosis. Under this circumstance, AURKA binds to SMC2 and subsequently phosphorylates SMC2 at T574 to disrupt the binding between SMC2 with SMC4 and the binding of SMC2/SMC4 to chromosomal DNA, resulting in mitotic catastrophe (Fig. 7). Our study provides new insights into the function of pre-rRNAs during mitosis. We discovered a new phosphorylation site on SMC2 and provided a new idea for tumor treatment strategy targeting SMC2.

## Materials and methods

### Cell culture and transfection

HeLa cells were maintained in DMEM supplemented with 10% fetal bovine serum. GFP-H2B+RFP-*α*-tubulin HeLa cells were a gift from D. W. Gerlich, institute of Biochemistry, Swiss Federal Institute of Techonology Zurich, Switzerland. All cell lines were purchased from the cell bank of the Chinese Academy of Medical Sciences and were routinely tested for Mycoplasma contamination. Cells were transfected with plasmids or siRNAs using Lipofectamine 2000 (Invitrogen) according to the manufacturer’s protocol. In the transient transfection experiments, the concentration of plasmids was kept at constant level using empty vectors.

Sequence of AURKA siRNAs: siRNA-1 5′-GUAUUUCAGGACCUGUUAA-3′, siRNA-2 5′-GGUCUUGUGUCCUUCAAAU-3′ (designed in http://biodev.extra.cea.fr/DSIR/DSIR.html (DSIR)). Sequence of SMC2 siRNAs: siRNA-1 5′-GACCCAAGACAGUAUCGAAA-3′, siRNA-2 5′-CACUGUCAAUAUUAAAGUAUA-3′ (designed in DSIR).

### Plasmid construction

All plasmids cloned with PCR inserts were confirmed by DNA sequencing. Flag-AURKA, Flag-SMC2 WT, Flag-SMC2 T574A, and Flag-SMC2 T574D were cloned into the pCI-neo vector in our laboratory. GST-SMC2, GST-SMC2-1, GST-SMC2-2, GST-SMC2-3, GST-SMC2-4, GST-SMC2-5 and GST-SMC2 T574A were cloned into the pGEX-4T1 vector in our laboratory. GFP-SMC2 was cloned into pEGFP-C3 vector in our laboratory. Site-mutated mutant plasmids, including Flag-SMC2 T574A and Flag-SMC2 T574D, were obtained by mutagenesis using the Quick-Change Site-Directed Mutagenesis Kit (Stratagene) according to the manufacturer’s protocol. The presence of mutations in the constructed plasmids was confirmed by DNA sequencing.

### Antibodies and reagents

Commercial antibodies used were anti-AURKA (ABclonal, A2121; Proteintech, 66757-1-lg), anti-SKP1 (ABclonal, A9021), anti-eEF1G (ABclonal, A7891), anti-TPX2 (ABclonal, A18327), anti-GNL3L (HUABIO, ER63552), anti-α-tubulin (ABclonal, AC012), anti-H3 (ABclonal, A2348), anti-Lamin B1 (ABclonal, A1910), anti-SMC2 (ABclonal, A17867; NOVUS, NB100-373), anti-SMC4 (ABclonal, A3559), anti-P-AURKA (Cell Signaling Technology, 3079T), anti-Flag (Cell Signaling Technology, 8146S) and anti- Pan-Phospho-Serine/Threonine (ABclonal, AP0893; Cell Signaling Technology, 9631). Anti-rabbit or anti-mouse secondary antibodies were from LI-COR (926-68071 and 926-32210). Actinomycin D (Act D, SBR00013) was purchased from Sigma. BMH-21 (HY-12484) and CX5461 (HY-13323) were purchased from MedChemExpress. TCS7010 (S1451) was purchased from Selleck. Thymidine (T1895) and nocodazole (N219) were purchased from Sigma-Aldrich.

### Cellular extraction and Western blotting

Cells were harvested and lysed in a lysis buffer containing 50 mM Tris-Cl, pH 7.4, 250 mM NaCl, 0.5% NP-40, 1 mM PMSF, 1 mM Na_3_VO_4_, 1 mM EDTA, 1 mM NaF, and cocktail of protease inhibitors [66]. After centrifugation at 12,000 rpm for 30 min at 4 °C to clear the lysate, protein quantification was performed using Coomassie Brilliant Blue G250 (Beyotime). Proteins in the cell fractions were separated by SDS-PAGE and transferred to nitrocellulose transfer membrane (Amersham). After blocking the membrane with 5% milk in PBS, the corresponding primary antibody was used for detection. After thoroughly washing with PBS/T (PBS buffer containing 0.5% Tween-20), the membrane was incubated with IRDye^®^ 800CW or 680RD secondary antibody (LI-COR). The fluorescent signals were detected using the Odyssey^®^ CLx imaging system. Images were acquired using Image Studio 5.0 (LI-COR).

### Live cell imaging

The mitotic process of GFP-H2B+RFP-α-tubulin HeLa cells was monitored in live cells grown in 35 mm glass bottom culture dishes (430165, Corning) using UltraVIEW VoX (PerkinElmer, USA). Images were captured every 10 min for a duration of 16–20 h using a 63×objective lens in a humidified incubator at 37°C with 5% CO_2_. The videos were acquired using Volocity software.

### Cell synchronization

Cell synchronization was performed as previously described [26]. Cells were synchronized at the G1/S transition after being treated with 2.5 mM thymidine for 24 h. To synchronize the cells at the M phase, following the 24-h treatment with 2.5 mM thymidine, the cells were washed three times with PBS and then incubated with 50 ng/mL nocodazole for 12 h. Mitotic cells were collected by shaking off.

### Immunofluorescence and RNA fluorescence in situ hybridization (FISH)

Immunofluorescence and RNA fluorescence in situ hybridization were performed as previously described [26]. For immunofluorescence, HeLa cells grown on glass slides were fixed in ice-cold 4% paraformaldehyde (PFA) in PBS and permeabilized in 0.5% Triton X-100. Subsequently, the cells were blocked in PBS containing 5% goat serum and 0.1% Triton X-100, followed by overnight incubation at 4 °C with the appropriate primary antibodies. After washing with PBS, the cells were incubated with secondary antibodies labeled with DyLight 488 or DyLight 594 (1:100) (Earthox). The nuclei and chromosomes were stained with DAPI (Beyotime), and the coverslips were mounted. Images were acquired using confocal microscopy (TCS-SP8 DIVE, Leica). For data analysis, cellular counting and intensity evaluation were conducted using ImageJ software.

For FISH analysis to detect the localization of pre-rRNAs, was performed as previously described [27]. Briefly, cells were fixed with 4% paraformaldehyde (PFA) and subsequently permeabilized using 0.5% Triton X-100. The cells were then incubated with 200 ng/mL of Cy3-labeled pre-rRNA probe (pre-rRNA probe-1: GGACGAGAATCACGAGCGACG, pre-rRNA probe-2: GCGAAGACGGAGAGGGAAAGA) in hybridization buffer (comprising 2×SSC, 50% deionized formamide, 10% dextran sulfate, 1×Denhardt’s solution, and 12.5 μg/mL ssDNA) at 37 °C overnight. Following hybridization, the cells were rinsed twice with 50% formamide in 2×SSC at 37 °C and then washed twice with 2×SSC at 37 °C for 10 min each. The cells were subsequently washed with PBS and stained with DAPI. The slides were mounted, and images were captured using confocal microscopy (TCS-SP8 DIVE, Leica).

### Depletion of pre-rRNAs by siRNAs in mitotic cells

HeLa cells were synchronized at the S phase by double thymidine blocking, and transfected with 30S or/and 32S pre-rRNA siRNAs, and simultaneously released in the medium containing 10 μM Ro-3306 (a selective CDK1 inhibitor, Sigma, SML0569) for 8 h to keep the cells at G2 phase. Then cells entered into M phase by placed in the fresh medium, which was prepared for immunofluorescent staining, FISH, and RT-qPCR.

Sequence of pre-rRNA siRNAs: siRNA-1 5′-GGACGAGAAUCACGAGCGACG-3′, siRNA-2 5′-GCGAAGACGGAGAGGGAAAGA-3′ (designed in DSIR).

### Puromycin incorporation assay

Puromycin incorporation assay was performed as previously described [67]. Briefly, HeLa cells were treated with BMH-21 for 0 h, 4 h, 8 h, 12 h, 16 h, 20 h, and 24 h. Before harvesting, these cells were treated with 10 μg/mL puromycin for 15 min. Then cell lysates were subjected to Western blot and probed with anti-puromycin antibody to evaluate nascent proteins.

### Chromosome fractionation

Chromosome fractionation was performed as previously described [36]. Briefly, cells were washed with cold PBS and subsequently suspended in buffer A, which contained 10 mM HEPES (pH 7.0), 100 mM NaCl, 300 mM sucrose, 3 mM MgCl_2_, an EDTA-free protease inhibitor cocktail, and 0.7% Triton X-100. After incubating on ice for 20 min, the lysate was centrifuged at 1500 × *g* for 4 min at 4 °C. The resulting supernatant was designated as S2. The nuclei were then washed once with buffer A and lysed in 200 μl of buffer B, which comprised 3 mM EDTA, 0.2 mM EGTA, 1 mM dithiothreitol, and a protease inhibitor mixture. Following a 10 min incubation on ice, soluble nuclear proteins (S3) were separated from chromatin by centrifugation at 2000 × *g* for 5 min. The isolated chromatin (P4) was washed once with buffer B and centrifuged at high speed (13,000 × *g*) for 10 min.

### Metaphase chromosome spread

Metaphase chromosome spread was performed as previously described [68]. HeLa cells were collected, washed with PBS, and subsequently treated with 75 mM KCl for 20 min at 37 °C. The samples were then fixed in ice-cold Carnoy’s fixative (a 3:1 mixture of methanol and glacial acetic acid) for 10 min and washed three times with Carnoy’s fixative. Then drop the fixed cells onto a glass slide from a height of 30–40 cm.

### Quantitative real-time PCR

Total RNA was extracted from the cells utilizing the TRIzol reagent (Invitrogen). Subsequently, cDNA was synthesized from the extracted total RNA using the Hifair^®^ III 1st Strand cDNA Synthesis SuperMix (YEASEN). The quantitative polymerase chain reaction (qPCR) analysis was conducted using the Hieff UNICON^®^ Universal Blue qPCR SYBR Green Master Mix from YEASEN, in conjunction with the ABI 7500/7500 Fast Real-time PCR System manufactured by Applied Biosystems. The human β-actin mRNA was amplified and served as an internal control. All real-time PCR data were meticulously analyzed using the comparative C_t_ method and normalized against β-actin.

### Protein purification

Protein purification was performed as previously described [69]. The expression plasmid was transformed into *Escherichia coli* strain BL21 (DE3, TransGen). Protein expression was subsequently induced with 0.1 mM of isopropy-β-D-thiogalactoside (IPTG) for a duration of 4 h at 37 °C. The *E. coli* cells were resuspended in a lysis buffer comprising 20 mM Tris-Cl (pH 7.5), 500 mM KCl, 10% glycerol, 0.1% Triton X-100, 5 mM imidazole, and a freshly prepared protease inhibitor cocktail. Following centrifugation and sonication, the proteins were purified using Ni-NTA Agarose (QIAGEN) and eluted with 250 mM imidazole. To eliminate any contaminating RNA, the purified proteins were further treated with 0.1 mg/ml RNase A (TIANGEN). Subsequently, the proteins were concentrated using Amicon Ultra centrifugal filters (Millipore). Finally, the proteins were separated by SDS-PAGE and visualized using Coomassie Blue staining.

### In vitro phosphorylation experiment

In vitro phosphorylation experiment was performed as previously described [39]. The AURKA kinase (pka-350-b) was purchased from Prospec. The kinase reactions were performed in 40 µL of 1 × kinase buffer (25 mM HEPES, pH 7.2, 50 mM NaCl, 2 mM EGTA, 5 mM MgSO_4_, 1 mM DTT, 0.01% Brij35) containing 200 ng AURKA kinase, 5 µg purified GST-SMC2 protein, and 50 µM ATP. The mixtures were incubated at 30 °C for 30 min, and the reactions were stopped with 5×SDS-PAGE sample buffer. Proteins were separated by SDS-PAGE and detected by Western blotting.

### Co-immunoprecipitation experiment

Cells were harvested and resuspended in Buffer A, which comprised 25 mM Tris-Cl (pH 7.5), 150 mM KCl, 1 mM DTT, 2 mM EDTA, 0.5 mM PMSF, 0.5% NP-40, and proteinase inhibitors. Subsequently, the cells were subjected to sonication. Following centrifugation, the supernatant was collected and utilized for immunoprecipitation. Antibodies were conjugated to protein A-Sepharose beads (GE Healthcare) in IPP500 buffer, consisting of 500 mM NaCl, 10 mM Tris-Cl (pH 8.0), and 0.5% NP-40. The conjugated beads were then incubated with the cell supernatant. After washing, the precipitated proteins were analyzed by Western blot using the indicated antibodies.

### GST pull-down experiment

GST pull-down experiment was performed as previously described [70]. GST fusion proteins were prepared according to the established protocol. For conducting in vitro binding assays, the GST fusion proteins, which were bound to Glutathione Sepharose 4B (GE Healthcare), were incubated with purified His-tagged proteins in a GST binding buffer that contained 100 mM NaCl, 2 mM EDTA, 20 mM Tris-HCl (pH 7.5), 2 mM DTT, 5% glycerol, and 1% NP-40. Following the washing step, the bound proteins were separated using SDS-PAGE and subsequently immunoblotted with the indicated antibodies.

### Chromatin immunoprecipitation

Chromatin immunoprecipitation was performed as previously described [42]. 1% formaldehyde was added to the cells to cross-link nuclear proteins with genomic DNA, and this reaction was terminated by the addition of 0.125 M glycine. The cells were then harvested by centrifugation and resuspended in ChIP buffer, which contained 50 mM HEPES-KOH (pH 7.5), 140 mM NaCl, 1% Triton X-100, 0.1% Sodium Deoxycholate, 0.1% SDS, 1 mM EDTA, and protease inhibitors. The genomic DNA was sonicated to achieve fragments of approximately 300–1000 bp in length. The cell supernatants were collected by centrifugation and diluted 1:10 with RIPA buffer (comprising 50 mM Tris-HCl pH 8.0, 150 mM NaCl, 2 mM EDTA, pH 8.0, 1% NP-40, 0.5% Sodium Deoxycholate, 0.1% SDS, and protease inhibitors). These supernatants were then incubated with the indicated antibodies for 1 h at 4 °C with rotation. Protein A-Sepharose beads were added to each reaction mixture and incubated overnight at 4 °C with rotation. The beads were subsequently collected by centrifugation and washed once in low salt wash buffer (0.1% SDS, 1% Triton X-100, 2 mM EDTA, 150 mM NaCl, and 20 mM Tris-HCl pH 8.0), once in high salt wash buffer (0.1% SDS, 1% Triton X-100, 2 mM EDTA, 500 mM NaCl, and 20 mM Tris-HCl pH 8.0), and once in LiCl wash buffer (0.25 M LiCl, 1% NP-40, 1% Sodium Deoxycholate, 1 mM EDTA, and 10 mM Tris-HCl pH 8.0). To elute the DNA, elution buffer was added to the protein A beads, and the mixture was vortexed slowly for 15 min at 30 °C. The protein-DNA cross-links were reversed by incubating the mixture with 200 mM NaCl, RNase A, and proteinase K at 65 °C. The DNA was then purified using phenol: chloroform extraction and resolved by Agarose gel.

### Statistical analysis

Statistical analysis was performed using GraphPad Prism version 9.0.0 for Windows, developed by GraphPad Software (San Diego, California, USA; www.graphpad.com). For comparisons among more than two groups, one-way ANOVA was employed, followed by either Tukey’s or Newman-Keuls multiple comparisons test. For comparisons between two groups, *t*-test was used. The group size was determined to be the minimum necessary to define a functional parameter, and the variance was found to be similar across the groups. All data met the assumptions required for the tests and are presented in the figures with error bars representing the mean ± SD. *P* < 0.05, *P* < 0.01, *P* < 0.001, and *P* < 0.0001 were considered statistically significant.

## Supplementary information

Supplemental material

Video 1

Video 2

Video 3

Video 4

Table S1

Table S2

Table S3

Original Western blots

## Data Availability

The datasets used and/or analyzed during the current study are available from the corresponding author on reasonable request.

## References

[1] Nagaoka SI, Hassold TJ, Hunt PA. Human aneuploidy: mechanisms and new insights into an age-old problem. Nat Rev Genet. 2012;13:493–504. 22705668 10.1038/nrg3245 PMC3551553

[2] Vasudevan A, Schukken KM, Sausville EL, Girish V, Adebambo OA, Sheltzer JM. Aneuploidy as a promoter and suppressor of malignant growth. Nat Rev Cancer. 2021;21:89–103. 33432169 10.1038/s41568-020-00321-1

[3] Vitale I, Galluzzi L, Castedo M, Kroemer G. Mitotic catastrophe: a mechanism for avoiding genomic instability. Nat Rev Mol Cell Biol. 2011;12:385–92. 21527953 10.1038/nrm3115

[4] Castedo M, Perfettini JL, Roumier T, Andreau K, Medema R, Kroemer G. Cell death by mitotic catastrophe: a molecular definition. Oncogene. 2004;23:2825–37. 15077146 10.1038/sj.onc.1207528

[5] Cuylen S, Haering CH. Deciphering condensin action during chromosome segregation. Trends Cell Biol. 2011;21:552–9. 21763138 10.1016/j.tcb.2011.06.003

[6] Paul MR, Hochwagen A, Ercan S. Condensin action and compaction. Curr Genet. 2019;65:407–15. 30361853 10.1007/s00294-018-0899-4 PMC6421088

[7] Cuylen S, Metz J, Haering CH. Condensin structures chromosomal DNA through topological links. Nat Struct Mol Biol. 2011;18:894–901. 21765419 10.1038/nsmb.2087

[8] Goloborodko A, Imakaev MV, Marko JF, Mirny L. Compaction and segregation of sister chromatids via active loop extrusion. Elife. 2016;5:e14864. 10.7554/eLife.14864 PMC4914367 27192037

[9] Kinoshita K, Hirano T. Dynamic organization of mitotic chromosomes. Curr Opin Cell Biol. 2017;46:46–53. 28214612 10.1016/j.ceb.2017.01.006

[10] Schneider MWG, Gibson BA, Otsuka S, Spicer MFD, Petrovic M, Blaukopf C, et al. A mitotic chromatin phase transition prevents perforation by microtubules. Nature. 2022;609:183–90. 35922507 10.1038/s41586-022-05027-y PMC9433320

[11] Yuen KC, Gerton JL. Taking cohesin and condensin in context. PLoS Genet. 2018;14:e1007118. 29370184 10.1371/journal.pgen.1007118 PMC5784890

[12] Ball AR Jr, Schmiesing JA, Zhou C, Gregson HC, Okada Y, Doi T, et al. Identification of a chromosome-targeting domain in the human condensin subunit CNAP1/hCAP-D2/Eg7. Mol Cell Biol. 2002;22:5769–81. 12138188 10.1128/MCB.22.16.5769-5781.2002 PMC133980

[13] Stray JE, Lindsley JE. Biochemical analysis of the yeast condensin Smc2/4 complex: an ATPase that promotes knotting of circular DNA. J Biol Chem. 2003;278:26238–48. 12719426 10.1074/jbc.M302699200

[14] Barysz H, Kim JH, Chen ZA, Hudson DF, Rappsilber J, Gerloff DL, et al. Three-dimensional topology of the SMC2/SMC4 subcomplex from chicken condensin I revealed by cross-linking and molecular modelling. Open Biol. 2015;5:150005. 25716199 10.1098/rsob.150005 PMC4345284

[15] Pandey R, Abel S, Boucher M, Wall RJ, Zeeshan M, Rea E, et al. Plasmodium condensin core subunits SMC2/SMC4 mediate atypical mitosis and are essential for parasite proliferation and transmission. Cell Rep. 2020;30:1883–97.e6. 32049018 10.1016/j.celrep.2020.01.033 PMC7016506

[16] Eeftens JM, Katan AJ, Kschonsak M, Hassler M, de Wilde L, Dief EM, et al. Condensin Smc2-Smc4 dimers are flexible and dynamic. Cell Rep. 2016;14:1813–8. 26904946 10.1016/j.celrep.2016.01.063 PMC4785793

[17] Henras AK, Plisson-Chastang C, O’Donohue MF, Chakraborty A, Gleizes PE. An overview of pre-ribosomal RNA processing in eukaryotes. Wiley Interdiscip Rev RNA. 2015;6:225–42. 25346433 10.1002/wrna.1269 PMC4361047

[18] Ma K, Luo M, Xie G, Wang X, Li Q, Gao L, et al. Ribosomal RNA regulates chromosome clustering during mitosis. Cell Discov. 2022;8:51. 35637200 10.1038/s41421-022-00400-7 PMC9151767

[19] Hernandez-Verdun D, Gautier T. The chromosome periphery during mitosis. Bioessays. 1994;16:179–85. 8166671 10.1002/bies.950160308

[20] Samejima K, Samejima I, Vagnarelli P, Ogawa H, Vargiu G, Kelly DA, et al. Mitotic chromosomes are compacted laterally by KIF4 and condensin and axially by topoisomerase IIalpha. J Cell Biol. 2012;199:755–70. 23166350 10.1083/jcb.201202155 PMC3514791

[21] Booth DG, Beckett AJ, Molina O, Samejima I, Masumoto H, Kouprina N, et al. 3D-CLEM reveals that a major portion of mitotic chromosomes is not chromatin. Mol Cell. 2016;64:790–802. 27840028 10.1016/j.molcel.2016.10.009 PMC5128728

[22] Booth DG, Earnshaw WC. Ki-67 and the chromosome periphery compartment in mitosis. Trends Cell Biol. 2017;27:906–16. 28838621 10.1016/j.tcb.2017.08.001

[23] Boisvert FM, van Koningsbruggen S, Navascues J, Lamond AI. The multifunctional nucleolus. Nat Rev Mol Cell Biol. 2007;8:574–85. 17519961 10.1038/nrm2184

[24] Cuylen S, Blaukopf C, Politi AZ, Müller-Reichert T, Neumann B, Poser I, et al. Ki-67 acts as a biological surfactant to disperse mitotic chromosomes. Nature. 2016;535:308–12. 27362226 10.1038/nature18610 PMC4947524

[25] Cuylen-Haering S, Petrovic M, Hernandez-Armendariz A, Schneider MWG, Samwer M, Blaukopf C, et al. Chromosome clustering by Ki-67 excludes cytoplasm during nuclear assembly. Nature. 2020;587:285–90. 32879492 10.1038/s41586-020-2672-3 PMC7666080

[26] Jiang Y, Sun S, Liu X, Su K, Zhang C, Zhang P, et al. U3 snoRNA inter-regulates with DDX21 in the perichromosomal region to control mitosis. Cell Death Dis. 2024;15:342. 38760378 10.1038/s41419-024-06725-3 PMC11101645

[27] Sirri V, Jourdan N, Hernandez-Verdun D, Roussel P. Sharing of mitotic pre-ribosomal particles between daughter cells. J Cell Sci. 2016;129:1592–604. 26929073 10.1242/jcs.180521

[28] Hayashi Y, Kato K, Kimura K. The hierarchical structure of the perichromosomal layer comprises Ki67, ribosomal RNAs, and nucleolar proteins. Biochem Biophys Res Commun. 2017;493:1043–9. 28935370 10.1016/j.bbrc.2017.09.092

[29] Fujimura A, Hayashi Y, Kato K, Kogure Y, Kameyama M, Shimamoto H, et al. Identification of a novel nucleolar protein complex required for mitotic chromosome segregation through centromeric accumulation of Aurora B. Nucleic Acids Res. 2020;48:6583–96. 32479628 10.1093/nar/gkaa449 PMC7337965

[30] Nikonova AS, Astsaturov I, Serebriiskii IG, Dunbrack RL Jr, Golemis EA. Aurora A kinase (AURKA) in normal and pathological cell division. Cell Mol Life Sci. 2013;70:661–87. 22864622 10.1007/s00018-012-1073-7 PMC3607959

[31] Hannak E, Kirkham M, Hyman AA, Oegema K. Aurora-A kinase is required for centrosome maturation in Caenorhabditis elegans. J Cell Biol. 2001;155:1109–16. 11748251 10.1083/jcb.200108051 PMC2199344

[32] Dutertre S, Cazales M, Quaranta M, Froment C, Trabut V, Dozier C, et al. Phosphorylation of CDC25B by Aurora-A at the centrosome contributes to the G2-M transition. J Cell Sci. 2004;117:2523–31. 15128871 10.1242/jcs.01108

[33] Toso A, Winter JR, Garrod AJ, Amaro AC, Meraldi P, McAinsh AD. Kinetochore-generated pushing forces separate centrosomes during bipolar spindle assembly. J Cell Biol. 2009;184:365–72. 19204145 10.1083/jcb.200809055 PMC2646558

[34] Hegarat N, Smith E, Nayak G, Takeda S, Eyers PA, Hochegger H. Aurora A and Aurora B jointly coordinate chromosome segregation and anaphase microtubule dynamics. J Cell Biol. 2011;195:1103–13. 22184196 10.1083/jcb.201105058 PMC3246887

[35] Li S, Deng Z, Fu J, Xu C, Xin G, Wu Z, et al. Spatial compartmentalization specializes the function of Aurora A and Aurora B. J Biol Chem. 2015;290:17546–58. 25987563 10.1074/jbc.M115.652453 PMC4498088

[36] Hao S, Wang Y, Zhao Y, Gao W, Cui W, Li Y, et al. Dynamic switching of crotonylation to ubiquitination of H2A at lysine 119 attenuates transcription-replication conflicts caused by replication stress. Nucleic Acids Res. 2022;50:9873–92. 36062559 10.1093/nar/gkac734 PMC9508856

[37] Kufer TA, Silljé HH, Körner R, Gruss OJ, Meraldi P, Nigg EA. Human TPX2 is required for targeting Aurora-A kinase to the spindle. J Cell Biol. 2002;158:617–23. 12177045 10.1083/jcb.200204155 PMC2174010

[38] Ferrari S, Marin O, Pagano MA, Meggio F, Hess D, El-Shemerly M, et al. Aurora-A site specificity: a study with synthetic peptide substrates. Biochem J. 2005;390:293–302. 16083426 10.1042/BJ20050343 PMC1188270

[39] Du R, Huang C, Chen H, Liu K, Xiang P, Yao N, et al. SDCBP/MDA-9/syntenin phosphorylation by AURKA promotes esophageal squamous cell carcinoma progression through the EGFR-PI3K-Akt signaling pathway. Oncogene. 2020;39:5405–19. 32572158 10.1038/s41388-020-1369-2

[40] Strunnikov AV, Hogan E, Koshland D. SMC2, a Saccharomyces cerevisiae gene essential for chromosome segregation and condensation, defines a subgroup within the SMC family. Genes Dev. 1995;9:587–99. 7698648 10.1101/gad.9.5.587

[41] Hirano M, Hirano T. Hinge-mediated dimerization of SMC protein is essential for its dynamic interaction with DNA. Embo J. 2002;21:5733–44. 12411491 10.1093/emboj/cdf575 PMC131072

[42] Wang Y, Su K, Wang C, Deng T, Liu X, Sun S, et al. Chemotherapy-induced acetylation of ACLY by NAT10 promotes its nuclear accumulation and acetyl-CoA production to drive chemoresistance in hepatocellular carcinoma. Cell Death Dis. 2024;15:545. 39085201 10.1038/s41419-024-06951-9 PMC11291975

[43] Fan H, Penman S. Regulation of synthesis and processing of nucleolar components in metaphase-arrested cells. J Mol Biol. 1971;59:27–42. 5283755 10.1016/0022-2836(71)90411-6

[44] Carron C, Balor S, Delavoie F, Plisson-Chastang C, Faubladier M, Gleizes PE, et al. Post-mitotic dynamics of pre-nucleolar bodies is driven by pre-rRNA processing. J Cell Sci. 2012;125:4532–42. 22767511 10.1242/jcs.106419

[45] Fu J, Bian M, Jiang Q, Zhang C. Roles of Aurora kinases in mitosis and tumorigenesis. Mol Cancer Res. 2007;5:1–10. 17259342 10.1158/1541-7786.MCR-06-0208

[46] Reboutier D, Troadec MB, Cremet JY, Chauvin L, Guen V, Salaun P, et al. Aurora A is involved in central spindle assembly through phosphorylation of Ser 19 in P150Glued. J Cell Biol. 2013;201:65–79. 23547029 10.1083/jcb.201210060 PMC3613693

[47] Stenoien DL, Sen S, Mancini MA, Brinkley BR. Dynamic association of a tumor amplified kinase, Aurora-A, with the centrosome and mitotic spindle. Cell Motil Cytoskeleton. 2003;55:134–46. 12740874 10.1002/cm.10120

[48] Jessberger R, Frei C, Gasser SM. Chromosome dynamics: the SMC protein family. Curr Opin Genet Dev. 1998;8:254–9. 9610418 10.1016/s0959-437x(98)80149-4

[49] Bazile F, St-Pierre J, D’Amours D. Three-step model for condensin activation during mitotic chromosome condensation. Cell Cycle. 2010;9:3243–55. 20703077 10.4161/cc.9.16.12620

[50] Takemoto A, Kimura K, Yokoyama S, Hanaoka F. Cell cycle-dependent phosphorylation, nuclear localization, and activation of human condensin. J Biol Chem. 2004;279:4551–9. 14607834 10.1074/jbc.M310925200

[51] Takemoto A, Kimura K, Yanagisawa J, Yokoyama S, Hanaoka F. Negative regulation of condensin I by CK2-mediated phosphorylation. Embo J. 2006;25:5339–48. 17066080 10.1038/sj.emboj.7601394 PMC1636611

[52] Chiu A, Revenkova E, Jessberger R. DNA interaction and dimerization of eukaryotic SMC hinge domains. J Biol Chem. 2004;279:26233–42. 15087462 10.1074/jbc.M402439200

[53] Alexander J, Lim D, Joughin BA, Hegemann B, Hutchins JR, Ehrenberger T, et al. Spatial exclusivity combined with positive and negative selection of phosphorylation motifs is the basis for context-dependent mitotic signaling. Sci Signal. 2011;4:ra42. 21712545 10.1126/scisignal.2001796 PMC3939359

[54] Ham MF, Takakuwa T, Rahadiani N, Tresnasari K, Nakajima H, Aozasa K. Condensin mutations and abnormal chromosomal structures in pyothorax-associated lymphoma. Cancer Sci. 2007;98:1041–7. 17488335 10.1111/j.1349-7006.2007.00500.x PMC11158810

[55] Dávalos V, Súarez-López L, Castaño J, Messent A, Abasolo I, Fernandez Y, et al. Human SMC2 protein, a core subunit of human condensin complex, is a novel transcriptional target of the WNT signaling pathway and a new therapeutic target. J Biol Chem. 2012;287:43472–81. 23095742 10.1074/jbc.M112.428466 PMC3527934

[56] Montero S, Seras-Franzoso J, Andrade F, Martinez-Trucharte F, Vilar-Hernández M, Quesada M, et al. Intracellular delivery of anti-SMC2 antibodies against cancer stem cells. Pharmaceutics. 2020;12:185. 10.3390/pharmaceutics12020185 PMC7076674 32098204

[57] Yang H, Li S, Li W, Yang Y, Zhang Y, Zhang S, et al. Actinomycin D synergizes with Doxorubicin in triple-negative breast cancer by inducing P53-dependent cell apoptosis. Carcinogenesis. 2024;45:262–73. 37997385 10.1093/carcin/bgad086

[58] Koh GCC, Boushaki S, Zhao SJ, Pregnall AM, Sadiyah F, Badja C, et al. The chemotherapeutic drug CX-5461 is a potent mutagen in cultured human cells. Nat Genet. 2024;56:23–6. 38036782 10.1038/s41588-023-01602-9 PMC10786719

[59] Peltonen K, Colis L, Liu H, Trivedi R, Moubarek MS, Moore HM, et al. A targeting modality for destruction of RNA polymerase I that possesses anticancer activity. Cancer Cell. 2014;25:77–90. 24434211 10.1016/j.ccr.2013.12.009 PMC3930145

[60] Xin D, Gai X, Ma Y, Li Z, Li Q, Yu X. Pre-rRNA facilitates TopBP1-mediated DNA double-strand break response. Adv Sci. 2023;10:e2206931. 10.1002/advs.202206931 PMC10558638 37582658

[61] Hernandez-Armendariz A, Sorichetti V, Hayashi Y, Koskova Z, Brunner A, Ellenberg J, et al. A liquid-like coat mediates chromosome clustering during mitotic exit. Mol Cell. 2024;84:3254–70.e9. 39153474 10.1016/j.molcel.2024.07.022

[62] Yamazaki H, Takagi M, Kosako H, Hirano T, Yoshimura SH. Cell cycle-specific phase separation regulated by protein charge blockiness. Nat Cell Biol. 2022;24:625–32. 35513709 10.1038/s41556-022-00903-1 PMC9106583

[63] Watada E, Li S, Hori Y, Fujiki K, Shirahige K, Inada T, et al. Age-dependent ribosomal DNA variations in mice. Mol Cell Biol. 2020;40:e00368–20. 10.1128/MCB.00368-20 PMC7588874 32900821

[64] Weaver KN, Watt KE, Hufnagel RB, Navajas Acedo J, Linscott LL, Sund KL, et al. Acrofacial dysostosis, Cincinnati type, a mandibulofacial dysostosis syndrome with limb anomalies, is caused by POLR1A dysfunction. Am J Hum Genet. 2015;96:765–74. 25913037 10.1016/j.ajhg.2015.03.011 PMC4570288

[65] Hori Y, Engel C, Kobayashi T. Regulation of ribosomal RNA gene copy number, transcription and nucleolus organization in eukaryotes. Nat Rev Mol Cell Biol. 2023;24:414–29. 36732602 10.1038/s41580-022-00573-9

[66] Liu X, Cai S, Zhang C, Liu Z, Luo J, Xing B, et al. Deacetylation of NAT10 by Sirt1 promotes the transition from rRNA biogenesis to autophagy upon energy stress. Nucleic Acids Res. 2018;46:9601–16. 30165671 10.1093/nar/gky777 PMC6182161

[67] Schmidt EK, Clavarino G, Ceppi M, Pierre P. SUnSET, a nonradioactive method to monitor protein synthesis. Nat Methods. 2009;6:275–7. 19305406 10.1038/nmeth.1314

[68] Pradella D, Zhang M, Gao R, Yao MA, Gluchowska KM, Cendon-Florez Y, et al. Engineered extrachromosomal oncogene amplifications promote tumorigenesis. Nature. 2024;637:955–964. 10.1038/s41586-024-08318-8 PMC11754114 39695225

[69] Su K, Zhao Z, Wang Y, Sun S, Liu X, Zhang C, et al. NAT10 resolves harmful nucleolar R-loops depending on its helicase domain and acetylation of DDX21. Cell Commun Signal. 2024;22:490. 39394182 10.1186/s12964-024-01869-3 PMC11468200

[70] Zheng J, Tan Y, Liu X, Zhang C, Su K, Jiang Y, et al. NAT10 regulates mitotic cell fate by acetylating Eg5 to control bipolar spindle assembly and chromosome segregation. Cell Death Differ. 2022;29:846–60. 35210604 10.1038/s41418-021-00899-5 PMC8989979

