# Supplementary material for: Pre-rRNAs control mitosis by maintaining chromosomal segregation through protecting SMC2 from AURKA-mediated phosphorylation
Source: Cell Death Dis. 2025 Nov 7;16(1):812. doi: 10.1038/s41419-025-08169-9 (PMC12594857; doi:10.1038/s41419-025-08169-9)
Supplement: Supplementary file 1 — Supplemental material [file 41419_2025_8169_MOESM1_ESM.docx]

**Supplementary data**


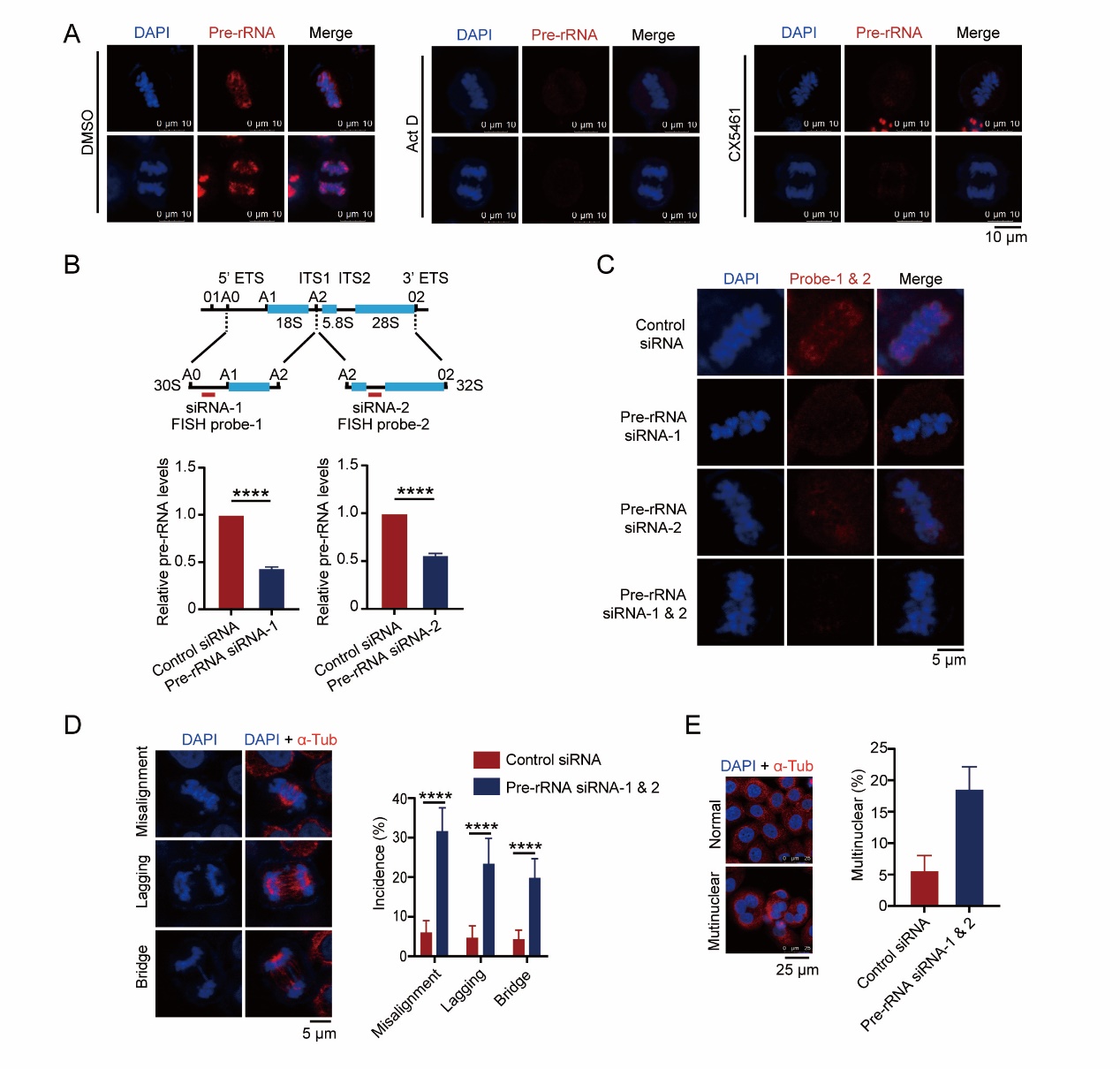


**Supplementary Figure 1. Pre-rRNAs depletion causes mitotic defects. A.** FISH was performed using a Cy3-labeled pre-rRNA probe in HeLa cells treated with the indicated reagents. Chromosomes were stained by DAPI. Scale bar, 10 μm. **B.** HeLa cells were transfected with either pre-rRNA siRNA-1 or pre-rRNA siRNA-2 as illustrated in the upper panel. HeLa cells were harvested, and RT-qPCR was performed to evaluate pre-rRNA levels (lower). **C.** FISH was performed using Cy3-labeled pre-rRNA probes described in (B) in HeLa cells transfected with the indicated pre-rRNA siRNAs. Chromosomes were stained by DAPI. Scale bar, 5 μm. **D.** Indirect immunostaining was performed in HeLa cells transfected with pre-rRNA siRNA-1 and 2 or control siRNA to visualize the mitotic spindle (α-Tubulin, red). Chromosomes were stained with DAPI (left). Scale bar, 5 μm. The frequency of chromosomal misalignment, lagging and bridge in these cells are shown (n > 100) (right). **E.** Immunofluorescent staining was performed with anti-α-Tubulin antibody in HeLa cells transfected with pre-rRNA siRNA-1 and 2 or control siRNA. Scale bar, 25 μm. A quantitative comparison of multinucleated cells in these cells is shown (n > 500). ***p* < 0.01. ****p* < 0.001. *****p* < 0.0001. n.s. denotes no significance.


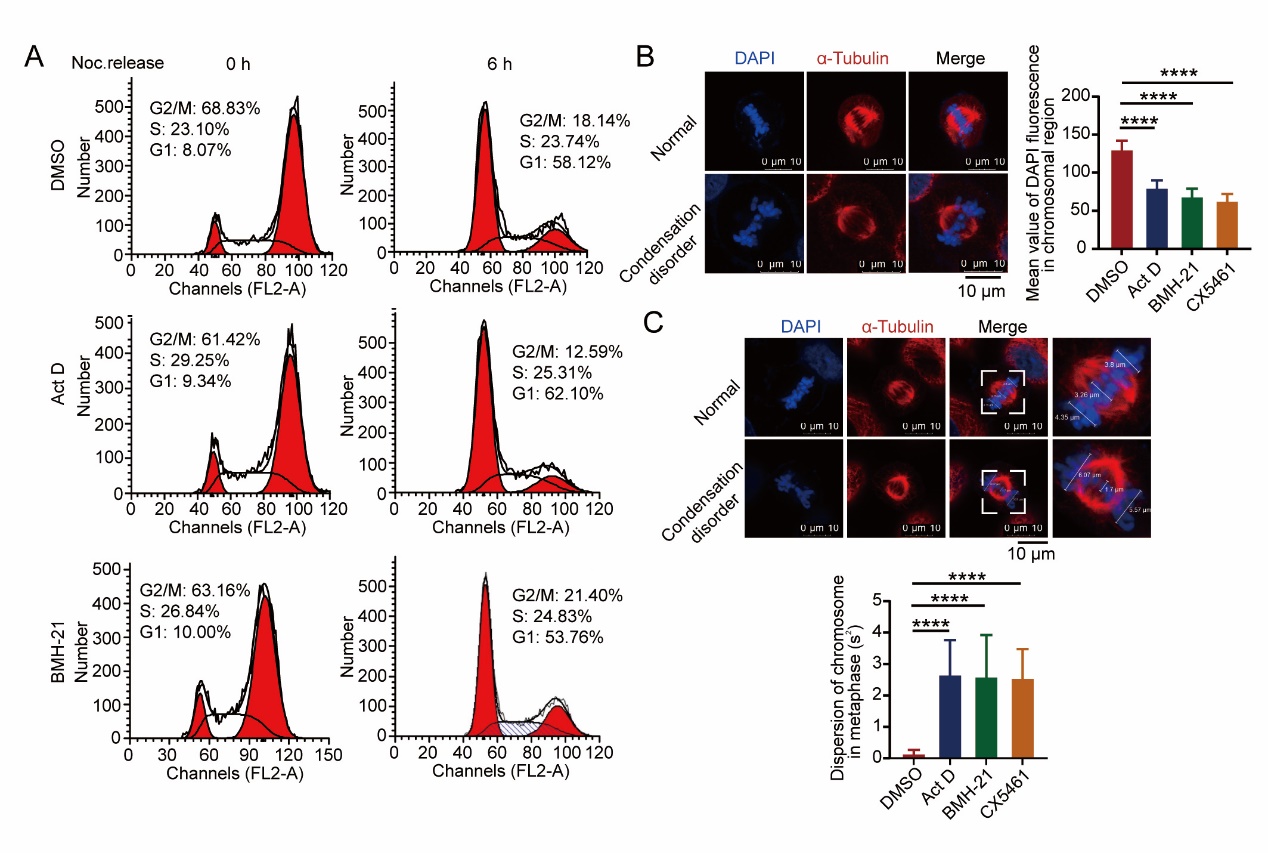


**Supplementary Figure 2. Pre-rRNAs depletion causes abnormal chromosomal condensation. A.** HeLa cells treated with the indicated reagents were synchronized to M phase using Thymidine-Nocodazole treatment. Mitotic cells were collected by shaking off and released into fresh medium for 6 h to next G1/S phase. Cell cycle of these synchronized cells was determined by flow cytometry. **B.** HeLa cells were treated with the indicated reagents. Immunofluorescent staining was performed with anti-α-Tubulin antibody in these cells. Chromosome was stained with DAPI (upper). Scale bar, 10 μm. Quantification of mean value of DAPI fluorescence in chromosomal region in cells as described above is shown (n = 15) (lower). **C.** HeLa cells were treated with the indicated reagents. Immunofluorescent staining was performed with anti-α-Tubulin antibody in these cells. Chromosomes were stained with DAPI. Scale bar, 10 μm. The ruler shows the width values of the chromosome at both ends and in the middle during the metaphase of mitosis. The variance (S^2^) of width values is calculated, compared, and shown in the graph (right). ***p* < 0.01. ****p* < 0.001. *****p* < 0.0001. n.s. denotes no significance.


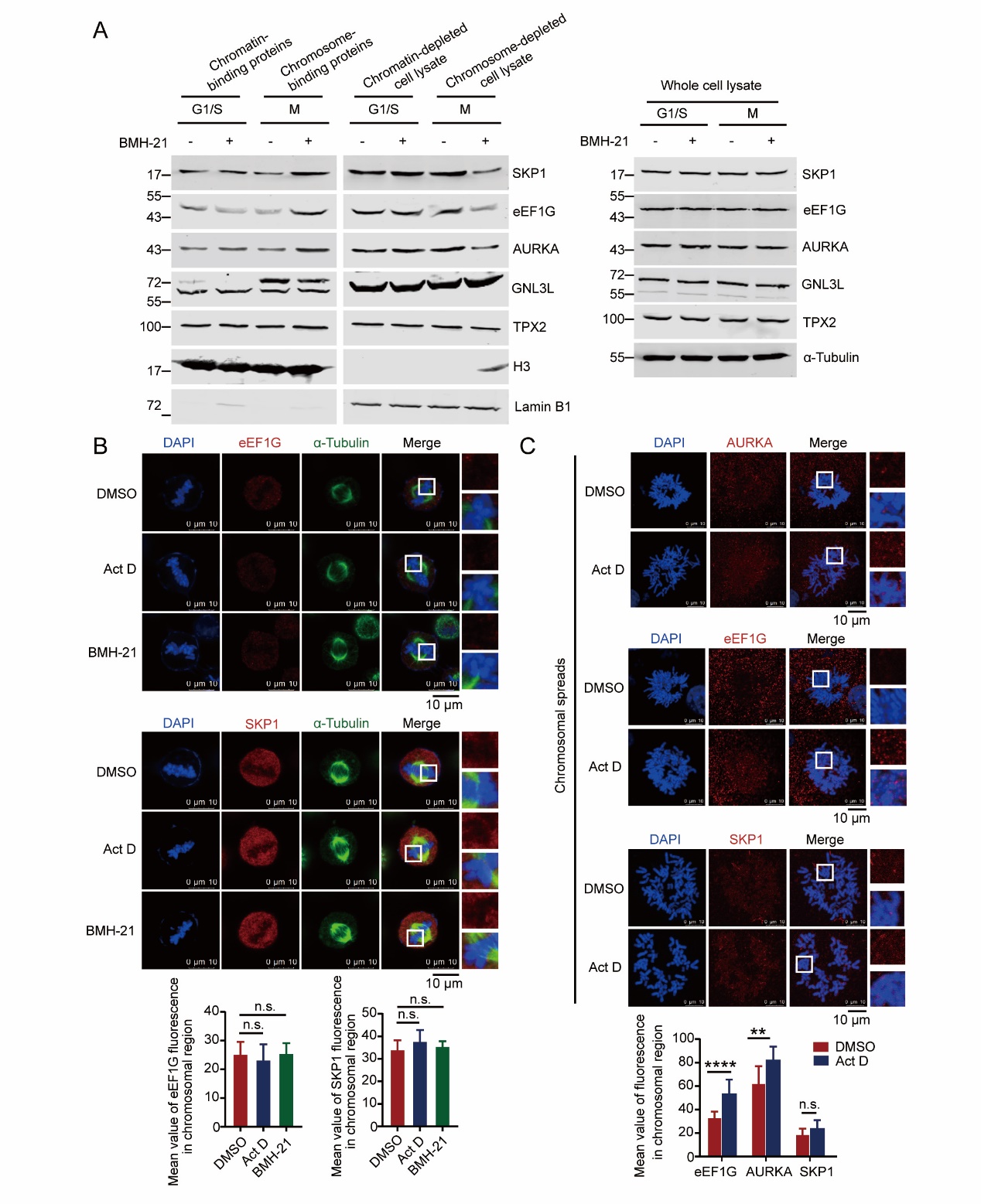


**Supplementary Figure 3. AURKA, not eEF1G or SKP1 approaches chromosomes when pre-rRNAs transcription is inhibited. A.** HeLa cells treated with 1 nM BMH-21 or DMSO were synchronized at G1/S phase by thymidine double blocking or at M phase by release for 8 h. The chromatin-binding proteins and chromatin-depleted cell lysate at G1/S phase, and the chromosome-binding proteins and chromosome-depleted cell lysate at M phase were collected by chromosome fractionation, and subjected to Western blot using indicated antibodies. H3 and lamin B1 were used as marker of chromatin (chromosome) and chromatin (chromosome)-depleted cell lysate, respectively (left). Cells were harvested and the proteins extracted from whole cell lysates were subjected to Western blot and probed with indicated antibodies. Alpha-Tubulin was used as a loading control (right). **B.** HeLa cells treated with the indicated reagents were fixed, and indirect immunofluorescent staining was performed using anti-α-tubulin, anti-eEF1G and anti-SKP1 antibodies. Chromosomes were stained by DAPI. Scale bar, 10 μm (upper). Quantifications of mean value of eEF1G and SKP1 fluorescent intensity in chromosomal region in the cells as described above are shown (n = 15) (lower). **C.** HeLa cells were treated with Act D or DMSO. Immunofluorescent staining on the chromosomal spreads was performed in the cells using anti-AURKA, anti-eEF1G or anti-SKP1 antibody. Chromosomes were stained with DAPI. Scale bar, 10 μm (upper). Quantification of mean value of AURKA, eEF1G and SKP1 fluorescent intensity in chromosomal region in the cells as described above is shown (n = 15) (lower).


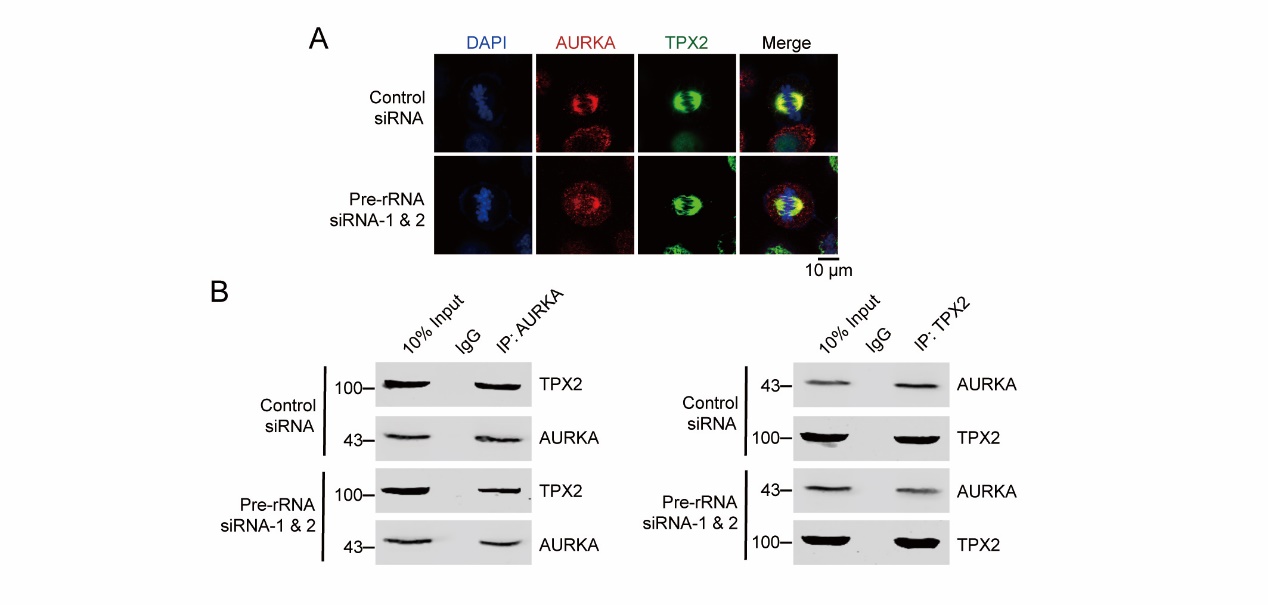


**Supplementary Figure 4. The mis-localization of AURKA did not induce alterations in the localization of TPX2 in the absence of pre-rRNAs during mitosis. A.** HeLa cells transfected with pre-rRNA siRNA-1 and 2 or control siRNA were fixed and indirect immunofluorescent staining was performed using anti-AURKA and anti-TPX2 antibodies. Chromosomes were stained by DAPI. Scale bar, 10 μm. **B.** HeLa cells transfected with t pre-rRNA siRNA-1 and 2 or control siRNA were synchronized at M phase and harvested. Immunoprecipitation was performed on the chromosome-binding proteins using indicated antibodies. The immunoprecipitates were immunoblotted with the indicated antibodies.


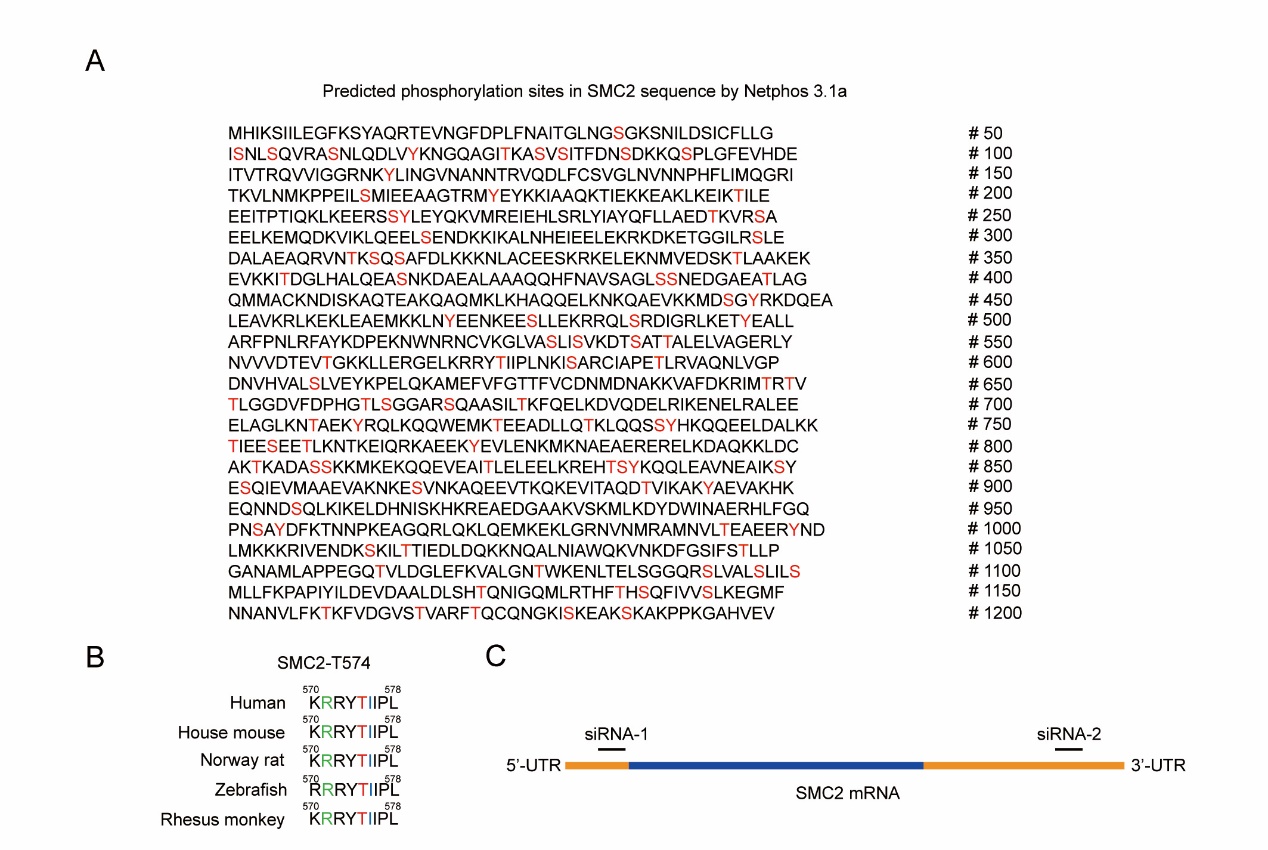


**Supplementary Figure 5. SMC2 T574 is conserved in higher species. A.** The amino acid sequence of SMC2 is displayed. The predicted phosphorylation sites by Netphos3.1a are highlighted in red. **B.** Sequence alignment of the AURKA phosphorylation consensus within SMC2 orthologs from different species. Phosphorylated threonine residues are highlighted in red, arginine at the n−3 position are highlighted in green, and hydrophobic residues at the n+1 position are highlighted in blue. **C.** The target sites of SMC2 siRNAs are shown in the schematic diagram.


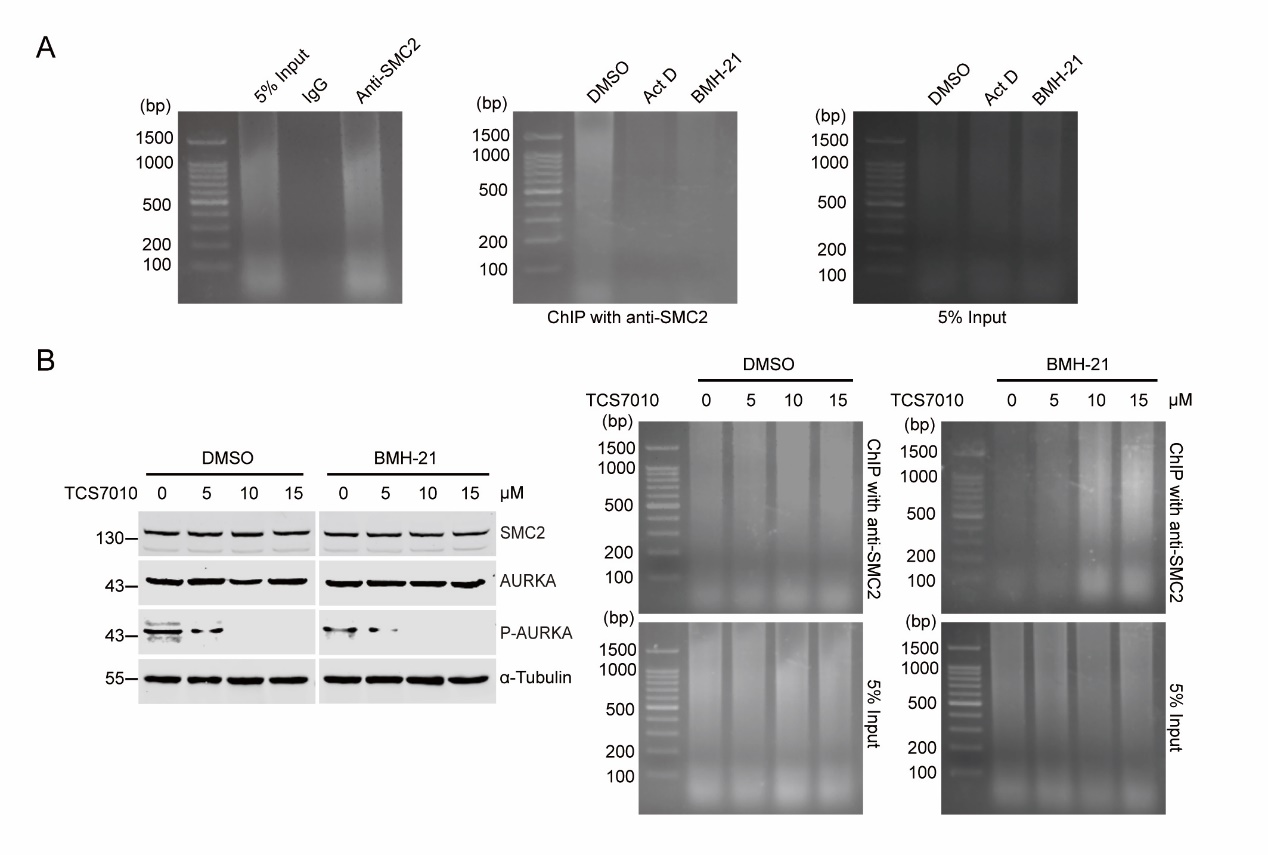


**Supplementary Figure 6. The phosphorylation of SMC2 by AURKA interferes with the binding of SMC2 with SMC4 and SMC2/SMC4 to DNA.** **A.** HeLa cells treated with the indicated reagents were synchronized in M phase by thymidine double blocking and harvested by shaking off. ChIP was performed with anti-SMC2 antibody and the DNA in the immunoprecipitates were resolved by Agarose gel. **B.** HeLa cells were treated with the indicated doses of TCS7010 and treated with BMH-21 or DMSO. The cells were synchronized in M phase and harvested as described in (C). Proteins were resolved by SDS-PAGE and probed with the indicated antibodies. Alpha-tubulin was used as a loading control (left). ChIP was performed with anti-SMC2 antibody and the DNA in the immunoprecipitates were resolved by Agarose gel (right).


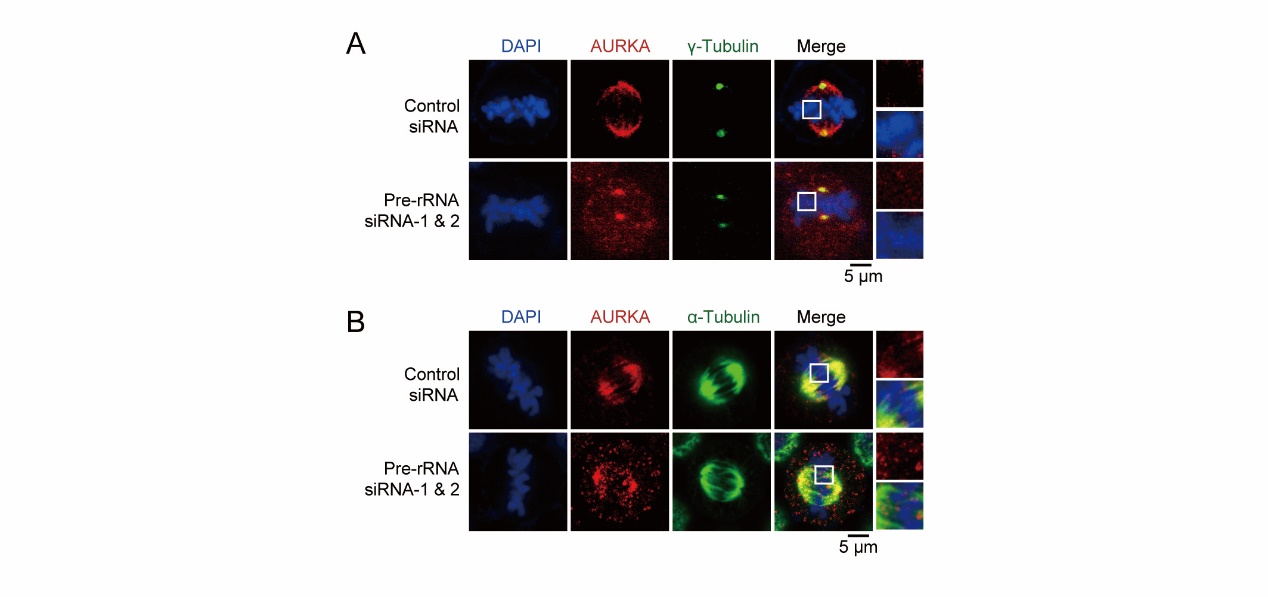


**Supplementary Figure 7. The mis-localization of AURKA caused by pre-rRNAs depletion did not affect mitotic centrosome and spindle assembly.** **A.** HeLa cells transfected with pre-rRNA siRNA-1 and 2 or control siRNA were fixed and indirect immunofluorescent staining was performed using anti-AURKA and anti-γ-tubulin antibodies. Chromosomes were stained by DAPI. Scale bar, 5 μm. **B.** HeLa cells transfected with pre-rRNA siRNA-1 and 2 or control siRNA were fixed and indirect immunofluorescent staining was performed using anti-AURKA and anti-α-tubulin antibodies. Chromosomes were stained by DAPI. Scale bar, 5 μm.


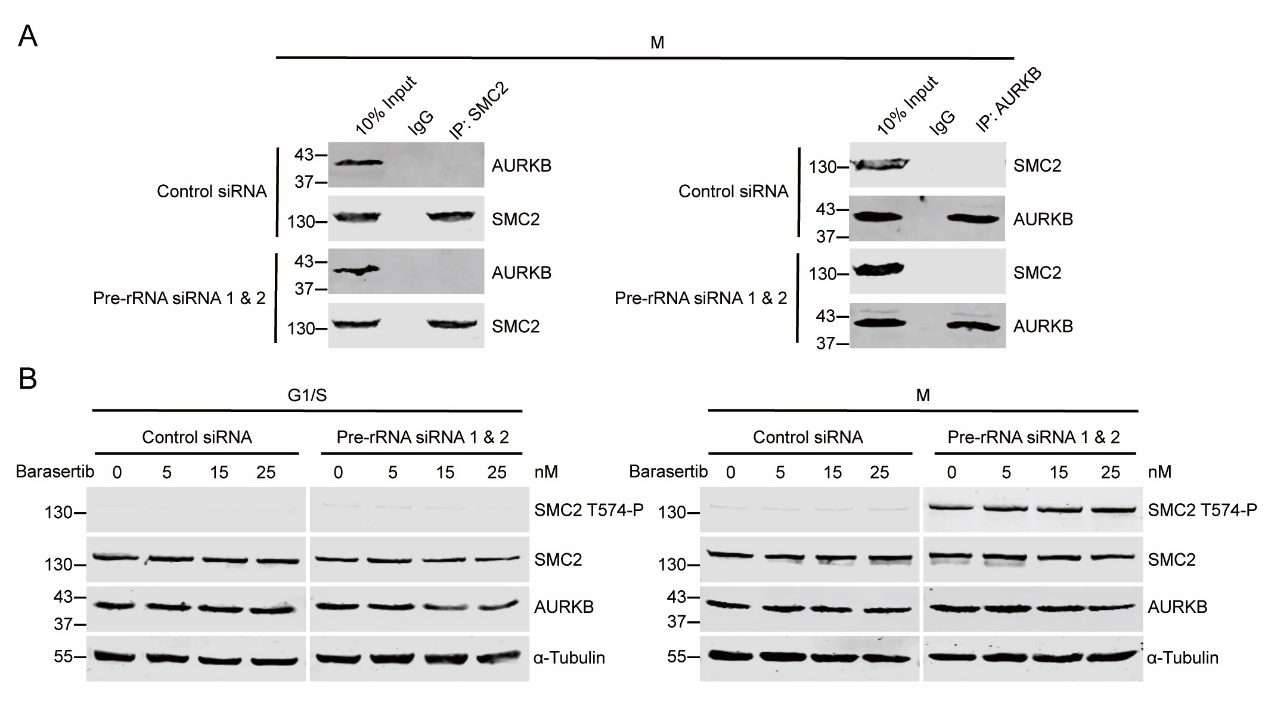


**Supplementary Figure 8. SMC2 T574 is not phosphorylated by AURKB.** **A.** HeLa cells transfected with pre-rRNA siRNA-1 and -2 or control siRNA were synchronized at M phase by thymidine double blocking and harvested by shaking off. Immunoprecipitation was performed on the chromosome-binding proteins using indicated antibodies. The immunoprecipitates were immunoblotted with the indicated antibodies. **B.** HeLa cells were treated with the indicated doses of barasertib and transfected with pre-rRNA siRNA-1 and 2 or control siRNA. These cells were synchronized and harvested as described in (A). Proteins were resolved by SDS-PAGE and probed with the indicated antibodies. Alpha-tubulin was used as a loading control.


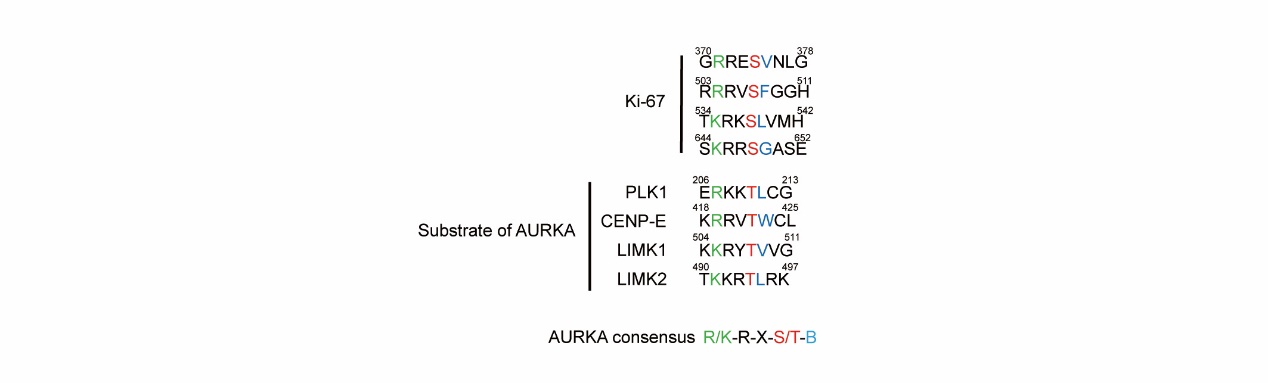


**Supplementary Figure 9. Ki-67 possesses a consensus of AURKA substrates.** Sequence alignment of the AURKA phosphorylation consensus within Ki-67 and substrates of AURKA. Phosphorylated threonine residues are highlighted in red, arginine at the n−3 position are highlighted in green, and hydrophobic residues at the n+1 position are highlighted in blue.


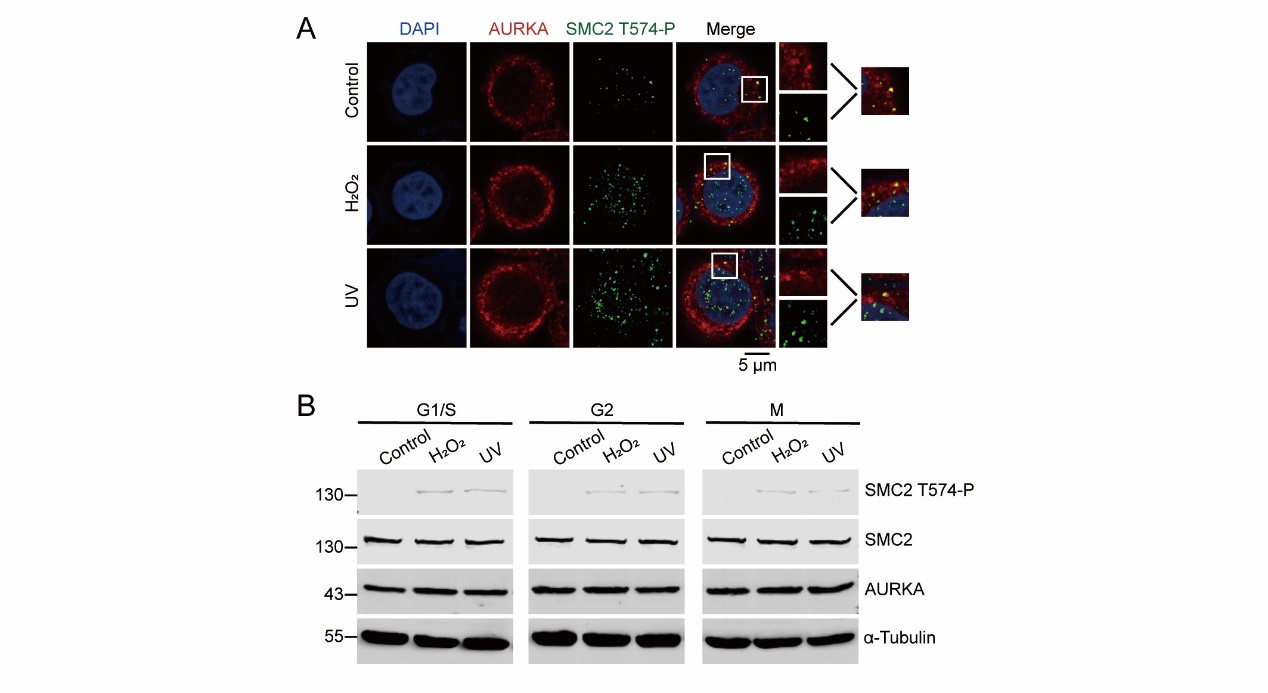


**Supplementary Figure 10. The phosphorylation of SMC2 T574 is induced by intracellular stress.** **A.** HeLa cells were treated with 100 μM H_2_O_2_ for 1 h or 500 J/m2 UV for 15 mins. These cells were fixed and indirect immunofluorescent staining was performed using anti-AURKA and anti-SMC2 T574-P antibody. Chromosomes were stained by DAPI. Scale bar, 5 μm. **B.** HeLa cells were treated with 100 μM H_2_O_2_ for 1 h or 500 J/m2 UV for 15 mins. These cells were synchronized at G1/S phase, G2 phase or M phase and harvested. Proteins were resolved by SDS-PAGE and probed with the indicated antibodies. Alpha-tubulin was used as a loading control.

**Supp Tables Legends**

Table S1. The results of quantitative proteomics analysis of decreased Chromosome-binding proteins in the absence of pre-rRNAs during mitosis in HeLa cells.

Table S2. The results of quantitative proteomics analysis of increased Chromosome-binding proteins in the absence of pre-rRNAs during mitosis in HeLa cells.

Table S3. The results of mass spectrum analysis of AURKA-binding proteins in chromosomal region in cells treated with Pol I inhibitor.

**Video Legends**

Video 1. The mitotic process of GFP-H2B+RFP-α-tubulin HeLa cells treated with DMSO.

Video 2. The mitotic process of GFP-H2B+RFP-α-tubulin HeLa cells treated with Act D.

Video 3. The mitotic process of GFP-H2B+RFP-α-tubulin HeLa cells treated with BMH-21.

Video 4. The mitotic process of GFP-H2B+RFP-α-tubulin HeLa cells treated with CX5461.
