# Supplementary material for: Pre-rRNAs control mitosis by maintaining chromosomal segregation through protecting SMC2 from AURKA-mediated phosphorylation
Source: Cell Death Dis. 2025 Nov 7;16(1):812. doi: 10.1038/s41419-025-08169-9 (PMC12594857; doi:10.1038/s41419-025-08169-9)
Supplement: Supplementary file 6 — Table S1 [file 41419_2025_8169_MOESM6_ESM.docx]

| **Decreased proteins from experiment 1** | | | | | | | | |
| --- | --- | --- | --- | --- | --- | --- | --- | --- |
| **Mitotic cells treated with DMSO** | | | **Mitotic cells treated with Act D** | | |  |  |  |
| LFQ intensity | LFQ intensity | LFQ intensity | LFQ intensity | LFQ intensity | LFQ intensity | Protein IDs | Protein names | Gene names |
| 28093000 | 29880000 | 35068000 | 0 | 0 | 0 | P63096 | Guanine nucleotide-binding protein G(i) subunit alpha-1 | GNAI1 |
| 27701000 | 30747000 | 31705000 | 0 | 0 | 0 | Q00577 | Transcriptional activator protein Pur-alpha | PURA |
| 18332000 | 11589000 | 10115000 | 0 | 0 | 0 | Q06210 | Glutamine--fructose-6-phosphate aminotransferase [isomerizing] 1 | GFPT1 |
| 109050000 | 51135000 | 97862000 | 0 | 0 | 0 | Q9BVK6 | Transmembrane emp24 domain-containing protein 9 | TMED9 |
| 34517000 | 38881000 | 41476000 | 0 | 0 | 0 | Q9H5I1 | Histone-lysine N-methyltransferase SUV39H2 | SUV39H2 |
| 118380000 | 88072000 | 89288000 | 0 | 0 | 0 | Q9Y6V7 | Probable ATP-dependent RNA helicase DDX49 | DDX49 |
| 540780000 | 1009500000 | 561980000 | 67317000 | 75771000 | 271470000 | P05026 | Sodium/potassium-transporting ATPase subunit beta-1 | ATP1B1 |
| 485700000 | 931930000 | 653300000 | 0 | 407440000 | 0 | Q9UJX3 | Anaphase-promoting complex subunit 7 | ANAPC7 |
| 175110000 | 200130000 | 216740000 | 55707000 | 50660000 | 49512000 | Q9ULW3 | Activator of basal transcription 1 | ABT1 |
| 577540000 | 529370000 | 544850000 | 159440000 | 151890000 | 189740000 | Q9Y2P8 | RNA 3-terminal phosphate cyclase-like protein | RCL1 |
| 2198300000 | 1932400000 | 1848300000 | 605360000 | 628090000 | 654030000 | Q9NY93 | Probable ATP-dependent RNA helicase DDX56 | DDX56 |
| 600530000 | 716280000 | 619190000 | 202050000 | 211170000 | 245010000 | Q9NZM5 | Glioma tumor suppressor candidate region gene 2 protein | GLTSCR2 |
| 273890000 | 355710000 | 351960000 | 107850000 | 121220000 | 109280000 | Q9H9L3 | Interferon-stimulated 20 kDa exonuclease-like 2 | ISG20L2 |
| 296940000 | 303550000 | 316660000 | 0 | 160300000 | 157480000 | Q9NVX2 | Notchless protein homolog 1 | NLE1 |
| 1653400000 | 1571800000 | 1673400000 | 614120000 | 564540000 | 548990000 | Q9BVJ6;Q5TAP6 | U3 small nucleolar RNA-associated protein 14 homolog A | UTP14A |
| 69059000 | 115750000 | 128010000 | 37546000 | 40023000 | 40940000 | Q9BRU9 | rRNA-processing protein UTP23 homolog | UTP23 |
| 1.0045E+10 | 9299500000 | 8995500000 | 3765800000 | 3425400000 | 3560400000 | O76021 | Ribosomal L1 domain-containing protein 1 | RSL1D1 |
| 1747600000 | 1619400000 | 1671600000 | 689060000 | 656720000 | 688130000 | Q14692 | Ribosome biogenesis protein BMS1 homolog | BMS1 |
| 1973300000 | 1700400000 | 1788400000 | 802040000 | 744040000 | 674700000 | Q9H6R4 | Nucleolar protein 6 | NOL6 |
| 306210000 | 326550000 | 321420000 | 123990000 | 124360000 | 140420000 | Q9NRX1 | RNA-binding protein PNO1 | PNO1 |
| 385700000 | 287090000 | 499930000 | 152510000 | 187720000 | 147100000 | Q9Y3A4;Q9NSQ0 | Ribosomal RNA-processing protein 7 homolog A | RRP7A |
| 325010000 | 404460000 | 404600000 | 133040000 | 195260000 | 145050000 | O95453 | Poly(A)-specific ribonuclease PARN | PARN |
| 1036500000 | 1018500000 | 906350000 | 394220000 | 439680000 | 433240000 | Q13895 | Bystin | BYSL |
| 222580000 | 252900000 | 213330000 | 85560000 | 83791000 | 129150000 | Q8IY37 | Probable ATP-dependent RNA helicase DHX37 | DHX37 |
| 893390000 | 728410000 | 759260000 | 331790000 | 327520000 | 388340000 | P42696 | RNA-binding protein 34 | RBM34 |
| 2074200000 | 1774600000 | 2095500000 | 900000000 | 936700000 | 795560000 | Q6DKI1 | 60S ribosomal protein L7-like 1 | RPL7L1 |
| 246080000 | 436960000 | 321180000 | 129800000 | 138770000 | 182020000 | O75683 | Surfeit locus protein 6 | SURF6 |
| 1767300000 | 1635100000 | 1583400000 | 748820000 | 716280000 | 821940000 | O75691 | Small subunit processome component 20 homolog | UTP20 |
| 221830000 | 321470000 | 224360000 | 162310000 | 103520000 | 99043000 | Q9Y324 | rRNA-processing protein FCF1 homolog | FCF1 |
| 1138800000 | 1144200000 | 1114800000 | 505970000 | 413280000 | 728980000 | Q9BVI4 | Nucleolar complex protein 4 homolog | NOC4L |
| 80651000 | 84394000 | 61671000 | 22248000 | 42634000 | 47978000 | O75348 | V-type proton ATPase subunit G 1 | ATP6V1G1 |
| 58426000 | 88999000 | 61817000 | 26305000 | 47419000 | 31568000 | Q9ULL5 | Proline-rich protein 12 | PRR12 |
| 5321900000 | 5834300000 | 5319400000 | 2710600000 | 2692600000 | 2903900000 | Q14690 | Protein RRP5 homolog | PDCD11 |
| 321810000 | 375100000 | 265790000 | 146350000 | 161500000 | 177990000 | Q9Y3A2 | Probable U3 small nucleolar RNA-associated protein 11 | UTP11L |
| 1043900000 | 916970000 | 890290000 | 499320000 | 475860000 | 494880000 | P78316 | Nucleolar protein 14 | NOP14 |
| 1663400000 | 1444600000 | 1588800000 | 670460000 | 858270000 | 909170000 | Q9NV06 | DDB1- and CUL4-associated factor 13 | DCAF13 |
| 3076200000 | 2422300000 | 2710000000 | 1626500000 | 1422200000 | 1226700000 | Q9BYG3 | MKI67 FHA domain-interacting nucleolar phosphoprotein | NIFK |
| 1165400000 | 1008600000 | 1174600000 | 555950000 | 458380000 | 759530000 | O95478 | Ribosome biogenesis protein NSA2 homolog | NSA2 |
| 651230000 | 686480000 | 682800000 | 327040000 | 375580000 | 370310000 | Q9Y2R4 | Probable ATP-dependent RNA helicase DDX52 | DDX52 |
| 390210000 | 427260000 | 388090000 | 202340000 | 219130000 | 220330000 | Q9BQ75 | Protein CMSS1 | CMSS1 |
| 2469600000 | 2337900000 | 2158800000 | 1336400000 | 910450000 | 1462600000 | Q9BVP2 | Guanine nucleotide-binding protein-like 3 | GNL3 |
| 1254600000 | 1297200000 | 1070100000 | 596110000 | 677790000 | 658060000 | O15213 | WD repeat-containing protein 46 | WDR46 |
| 1035400000 | 1126200000 | 1035000000 | 635210000 | 535860000 | 537480000 | Q9GZR2 | RNA exonuclease 4 | REXO4 |
| 1096400000 | 940340000 | 825120000 | 591470000 | 465330000 | 477880000 | Q9NYH9 | U3 small nucleolar RNA-associated protein 6 homolog | UTP6 |
| 572780000 | 500550000 | 560720000 | 247750000 | 321520000 | 310790000 | Q9Y4C8 | Probable RNA-binding protein 19 | RBM19 |
| 355090000 | 315070000 | 401420000 | 215180000 | 201770000 | 160200000 | O95347 | Structural maintenance of chromosomes protein 2 | SMC2 |
| 4080600000 | 4347000000 | 4080700000 | 2558700000 | 2297300000 | 2054200000 | Q15397 | Pumilio domain-containing protein KIAA0020 | KIAA0020 |
| 4651600000 | 5658800000 | 4833300000 | 2816400000 | 2507900000 | 3211800000 | Q5JTH9 | RRP12-like protein | RRP12 |
| 3384700000 | 2722300000 | 2783300000 | 1829200000 | 1603800000 | 1598500000 | O00541 | Pescadillo homolog | PES1 |
| 1747800000 | 1684500000 | 1564000000 | 967300000 | 979990000 | 917140000 | Q9NW13 | RNA-binding protein 28 | RBM28 |
| 486530000 | 403620000 | 404910000 | 228660000 | 244160000 | 272370000 | Q9H9Y2 | Ribosome production factor 1 | RPF1 |
| 917520000 | 885700000 | 780950000 | 453150000 | 482340000 | 562890000 | P53582 | Methionine aminopeptidase 1 | METAP1 |
| 730750000 | 620680000 | 695610000 | 466980000 | 348940000 | 378280000 | Q13610 | Periodic tryptophan protein 1 homolog | PWP1 |
| 5404400000 | 5089600000 | 5828400000 | 3298600000 | 3146100000 | 3079700000 | Q9BQG0 | Myb-binding protein 1A | MYBBP1A |
| 2842500000 | 3313800000 | 2873900000 | 1836000000 | 1703400000 | 1784500000 | Q9GZR7 | ATP-dependent RNA helicase DDX24 | DDX24 |
| 1395500000 | 1337100000 | 1281800000 | 766050000 | 901230000 | 718320000 | Q13823 | Nucleolar GTP-binding protein 2 | GNL2 |
| 985250000 | 899650000 | 833100000 | 582330000 | 535810000 | 523510000 | A0A0B4J1V8;Q9NQ55 | Suppressor of SWI4 1 homolog | PPAN-P2RY11;PPAN |
| 2502600000 | 2411200000 | 2655900000 | 1552500000 | 1525800000 | 1499600000 | Q99848 | Probable rRNA-processing protein EBP2 | EBNA1BP2 |
| 1490800000 | 1722100000 | 1667800000 | 1066700000 | 900290000 | 986360000 | O43818 | U3 small nucleolar RNA-interacting protein 2 | RRP9 |
| 618970000 | 595640000 | 520340000 | 440730000 | 321600000 | 289300000 | Q6RFH5 | WD repeat-containing protein 74 | WDR74 |
| 3913600000 | 3074400000 | 3294200000 | 2013400000 | 1742100000 | 2483000000 | Q96GQ7 | Probable ATP-dependent RNA helicase DDX27 | DDX27 |
| 341740000 | 500640000 | 414370000 | 229900000 | 244330000 | 293620000 | Q06265 | Exosome complex component RRP45 | EXOSC9 |
| 3451200000 | 3690800000 | 3778800000 | 2297000000 | 2206700000 | 2227000000 | Q9GZL7 | Ribosome biogenesis protein WDR12 | WDR12 |
| 1003500000 | 948730000 | 885250000 | 431680000 | 762530000 | 572590000 | Q86U38 | Nucleolar protein 9 | NOP9 |
| 4384900000 | 4051200000 | 3609000000 | 2437500000 | 2487800000 | 2631600000 | Q14137 | Ribosome biogenesis protein BOP1 | BOP1 |
| 2788900000 | 2839900000 | 3038900000 | 1697400000 | 1786900000 | 2030100000 | Q8TDD1 | ATP-dependent RNA helicase DDX54 | DDX54 |
| 1604100000 | 1376000000 | 1581300000 | 1058400000 | 963920000 | 910270000 | O00566 | U3 small nucleolar ribonucleoprotein protein MPP10 | MPHOSPH10 |
| 531970000 | 496350000 | 588550000 | 377510000 | 372750000 | 293390000 | Q8N9T8 | Protein KRI1 homolog | KRI1 |
| 644440000 | 659930000 | 613750000 | 410180000 | 446120000 | 383100000 | Q13601 | Structural maintenance of chromosomes protein 4 | SMC4 |
| 8187100000 | 9793400000 | 8557200000 | 5493100000 | 5783900000 | 5935800000 | Q8TDN6 | Ribosome biogenesis protein BRX1 homolog | BRIX1 |
| 60824000 | 46527000 | 62320000 | 33390000 | 37044000 | 40430000 | Q9Y4K0 | Lysyl oxidase homolog 2 | LOXL2 |
| 2589000000 | 2544900000 | 2654100000 | 2064100000 | 1589200000 | 1447500000 | Q9H0S4 | Probable ATP-dependent RNA helicase DDX47 | DDX47 |
| 568260000 | 535770000 | 492330000 | 387250000 | 323530000 | 343700000 | Q9BXY0 | Protein MAK16 homolog | MAK16 |
| 891450000 | 726110000 | 853830000 | 559600000 | 528980000 | 544010000 | Q5T280 | Putative methyltransferase C9orf114 | C9orf114 |
| 2068200000 | 2606700000 | 2341600000 | 1603500000 | 1584400000 | 1521900000 | Q9H7B2 | Ribosome production factor 2 homolog | RPF2 |
| 141890000 | 182220000 | 190920000 | 132420000 | 109150000 | 104690000 | O75319 | RNA/RNP complex-1-interacting phosphatase | DUSP11 |
| 1641000000 | 1405000000 | 1602600000 | 1086700000 | 899760000 | 1145700000 | O43159 | Ribosomal RNA-processing protein 8 | RRP8 |
| 3859800000 | 4094800000 | 3437200000 | 2743100000 | 2651200000 | 2356000000 | Q9UKD2 | mRNA turnover protein 4 homolog | MRTO4 |
| 358380000 | 357140000 | 331220000 | 303340000 | 218220000 | 192720000 | Q5RKV6 | Exosome complex component MTR3 | EXOSC6 |
| 1896100000 | 1905500000 | 1675700000 | 1268300000 | 1162000000 | 1318700000 | Q9Y5J1 | U3 small nucleolar RNA-associated protein 18 homolog | UTP18 |
| 366480000 | 508580000 | 411590000 | 274720000 | 281390000 | 327990000 | Q9Y3B9 | RRP15-like protein | RRP15 |
| 237650000 | 244800000 | 199250000 | 160400000 | 148360000 | 160330000 | Q9Y3B2 | Exosome complex component CSL4 | EXOSC1 |
| 552960000 | 520130000 | 525370000 | 389840000 | 430970000 | 290030000 | Q9NQT5 | Exosome complex component RRP40 | EXOSC3 |
| 169960000 | 197430000 | 154960000 | 127880000 | 121780000 | 113650000 | P78362 | SRSF protein kinase 2 | SRPK2 |
| 1986800000 | 2065900000 | 1867300000 | 1488000000 | 1319300000 | 1332900000 | Q9BSC4 | Nucleolar protein 10 | NOL10 |
| 6383700000 | 5901500000 | 5800400000 | 4726500000 | 3835900000 | 4108300000 | Q9NVP1 | ATP-dependent RNA helicase DDX18 | DDX18 |
| 647690000 | 571870000 | 535380000 | 387880000 | 379420000 | 462510000 | Q96G21 | U3 small nucleolar ribonucleoprotein protein IMP4 | IMP4 |
| 2102500000 | 2071900000 | 1971600000 | 1620000000 | 1152800000 | 1586200000 | Q9Y221 | 60S ribosome subunit biogenesis protein NIP7 homolog | NIP7 |
| 603140000 | 731970000 | 657560000 | 489540000 | 489820000 | 441090000 | Q15024 | Exosome complex component RRP42 | EXOSC7 |
| 860190000 | 963380000 | 900430000 | 705310000 | 689880000 | 561000000 | Q8NEJ9 | Neuroguidin | NGDN |
| 1478600000 | 1270400000 | 1299000000 | 1121200000 | 958880000 | 837910000 | Q9NWT1 | p21-activated protein kinase-interacting protein 1 | PAK1IP1 |
| 887990000 | 752160000 | 727260000 | 610770000 | 489610000 | 612370000 | Q92979 | Ribosomal RNA small subunit methyltransferase NEP1 | EMG1 |
| 492040000 | 547800000 | 494830000 | 431690000 | 365830000 | 312810000 | Q5T3I0 | G patch domain-containing protein 4 | GPATCH4 |
| 281090000 | 242470000 | 241330000 | 176950000 | 199610000 | 178370000 | Q9BRT6 | Protein LLP homolog | LLPH |
| 7581200000 | 7273300000 | 7227100000 | 5965800000 | 4766200000 | 5336600000 | O60832 | H/ACA ribonucleoprotein complex subunit 4 | DKC1 |
| 5445300000 | 5104700000 | 4943700000 | 3930900000 | 3440700000 | 3952500000 | P46087 | Probable 28S rRNA (cytosine(4447)-C(5))-methyltransferase | NOP2 |
| 94368000 | 102740000 | 80271000 | 65070000 | 63609000 | 74395000 | O94973 | AP-2 complex subunit alpha-2 | AP2A2 |
| 99869000 | 92532000 | 110520000 | 87510000 | 67851000 | 66715000 | Q92547 | DNA topoisomerase 2-binding protein 1 | TOPBP1 |
| 4584100000 | 4583200000 | 4460000000 | 3580000000 | 3366800000 | 3145100000 | Q12788 | Transducin beta-like protein 3 | TBL3 |
| 1.3611E+10 | 1.5503E+10 | 1.5144E+10 | 1.1587E+10 | 1.1319E+10 | 9927000000 | P19338 | Nucleolin | NCL |
| 2166000000 | 2006800000 | 2215300000 | 1681000000 | 1507800000 | 1560000000 | Q5QJE6 | Deoxynucleotidyltransferase terminal-interacting protein 2 | DNTTIP2 |
| 1290900000 | 1397800000 | 1512200000 | 997310000 | 976670000 | 1149900000 | Q969X6 | Cirhin | CIRH1A |
| 1927800000 | 1856600000 | 1851800000 | 1479600000 | 1382500000 | 1331900000 | Q9NY61 | Protein AATF | AATF |
| 73357000 | 74928000 | 64920000 | 60408000 | 55276000 | 43328000 | Q9Y618 | Nuclear receptor corepressor 2 | NCOR2 |
| 1595900000 | 1439600000 | 1436800000 | 1119400000 | 1039300000 | 1204100000 | Q01780 | Exosome component 10 | EXOSC10 |
| 41148000 | 49447000 | 40495000 | 34040000 | 32142000 | 32406000 | Q8IVS2 | Malonyl-CoA-acyl carrier protein transacylase, mitochondrial | MCAT |
| 1572500000 | 1600800000 | 1650000000 | 1210400000 | 1073900000 | 1348000000 | Q8NF91 | Nesprin-1 | SYNE1 |
| 3042100000 | 3202700000 | 3171400000 | 2276300000 | 2359300000 | 2482000000 | Q15269;A0A0B4J2E5 | Periodic tryptophan protein 2 homolog | PWP2 |
| 5828300000 | 5926600000 | 5168400000 | 4485000000 | 4140500000 | 4195500000 | Q9H0A0 | N-acetyltransferase 10 | NAT10 |
| 283060000 | 259250000 | 325120000 | 202190000 | 244890000 | 210190000 | E9PAV3;Q9BZK3 | Nascent polypeptide-associated complex subunit alpha, muscle-specific form | NACA |
| 163440000 | 194710000 | 204320000 | 140860000 | 140320000 | 145200000 | P46063 | ATP-dependent DNA helicase Q1 | RECQL |
| 3630800000 | 3709000000 | 3297000000 | 2699800000 | 2541700000 | 2822000000 | Q8WTT2 | Nucleolar complex protein 3 homolog | NOC3L |
| 8591300000 | 7394700000 | 7493600000 | 6148700000 | 5895200000 | 5927100000 | Q9BZE4 | Nucleolar GTP-binding protein 1 | GTPBP4 |
| 1248200000 | 1384800000 | 1225600000 | 1009900000 | 994930000 | 954070000 | Q13206 | Probable ATP-dependent RNA helicase DDX10 | DDX10 |
| 5228100000 | 4895500000 | 4574500000 | 3883000000 | 3617200000 | 3808600000 | Q9H583 | HEAT repeat-containing protein 1 | HEATR1 |
| 3119400000 | 3360600000 | 3132400000 | 2626200000 | 2389100000 | 2386000000 | Q14684 | Ribosomal RNA processing protein 1 homolog B | RRP1B |
| 5439300000 | 5363200000 | 6304900000 | 4460200000 | 4525600000 | 4300700000 | Q8IY81 | pre-rRNA processing protein FTSJ3 | FTSJ3 |
| 935380000 | 915310000 | 996100000 | 843910000 | 716070000 | 653370000 | Q92879;O95319 | CUGBP Elav-like family member 1 | CELF1 |
| 8369300000 | 9606100000 | 9089900000 | 6773800000 | 7054500000 | 7272600000 | Q9Y2X3 | Nucleolar protein 58 | NOP58 |
| 2284700000 | 1971400000 | 2309600000 | 1667200000 | 1553600000 | 1915900000 | Q15050 | Ribosome biogenesis regulatory protein homolog | RRS1 |
| 141990000 | 170800000 | 143130000 | 129540000 | 109720000 | 117600000 | Q9NUP9;O14910 | Protein lin-7 homolog C | LIN7C |
| 547020000 | 517810000 | 509320000 | 401170000 | 374130000 | 462170000 | Q9H6W3 | Bifunctional lysine-specific demethylase and histidyl-hydroxylase NO66 | NO66 |
| 63562000 | 64313000 | 71693000 | 46435000 | 57310000 | 53747000 | P63272 | Transcription elongation factor SPT4 | SUPT4H1 |
| 1121600000 | 1184100000 | 1146100000 | 886380000 | 871530000 | 980840000 | P56182 | Ribosomal RNA processing protein 1 homolog A | RRP1 |
| 1471200000 | 1292800000 | 1372000000 | 1246100000 | 988810000 | 1068100000 | Q9P275 | Ubiquitin carboxyl-terminal hydrolase 36 | USP36 |
| 1550200000 | 1264200000 | 1483000000 | 1224100000 | 1035200000 | 1173100000 | Q9Y3C1 | Nucleolar protein 16 | NOP16 |
| 3710000000 | 4322700000 | 4282100000 | 3526700000 | 3360600000 | 2967600000 | P46777 | 60S ribosomal protein L5 | RPL5 |
| 55397000 | 60677000 | 57118000 | 39674000 | 51446000 | 48525000 | Q63ZY3 | KN motif and ankyrin repeat domain-containing protein 2 | KANK2 |
| 219100000 | 225610000 | 258890000 | 194070000 | 179580000 | 195440000 | Q96B26 | Exosome complex component RRP43 | EXOSC8 |
| 2150300000 | 2210500000 | 2137100000 | 1821700000 | 1769300000 | 1666900000 | Q15061 | WD repeat-containing protein 43 | WDR43 |
| 1.236E+10 | 1.2375E+10 | 1.1833E+10 | 1.0293E+10 | 9663600000 | 9892900000 | O00567 | Nucleolar protein 56 | NOP56 |
| 887520000 | 1022200000 | 932590000 | 811740000 | 724310000 | 789060000 | Q9H6R0 | Putative ATP-dependent RNA helicase DHX33 | DHX33 |
| 471550000 | 508490000 | 519270000 | 407610000 | 407760000 | 425190000 | Q13868 | Exosome complex component RRP4 | EXOSC2 |
| 827300000 | 877830000 | 830040000 | 675890000 | 718160000 | 720070000 | Q99575 | Ribonucleases P/MRP protein subunit POP1 | POP1 |
| 52767000 | 60413000 | 54243000 | 45291000 | 50322000 | 44492000 | Q5T6S3 | PHD finger protein 19 | PHF19 |
| 4218400000 | 4387100000 | 4159000000 | 3862200000 | 3649700000 | 3249500000 | P61353 | 60S ribosomal protein L27 | RPL27 |
| 203360000 | 223150000 | 215470000 | 195720000 | 169820000 | 178130000 | P61513 | 60S ribosomal protein L37a | RPL37A |
| 2608600000 | 2667800000 | 2419600000 | 2125600000 | 2212100000 | 2229400000 | Q9Y3T9 | Nucleolar complex protein 2 homolog | NOC2L |
| 306150000 | 294300000 | 309640000 | 285690000 | 242100000 | 261260000 | O43248 | Homeobox protein Hox-C11 | HOXC11 |
| 197540000 | 217800000 | 217550000 | 182900000 | 183010000 | 182990000 | Q96QE3 | ATPase family AAA domain-containing protein 5 | ATAD5 |
| 741630000 | 734790000 | 790800000 | 704200000 | 657810000 | 620970000 | O15294 | UDP-N-acetylglucosamine--peptide N-acetylglucosaminyltransferase 110 kDa subunit | OGT |
| 2144100000 | 2260300000 | 2074500000 | 1840700000 | 1978200000 | 1864400000 | Q5SSJ5 | Heterochromatin protein 1-binding protein 3 | HP1BP3 |
| 262590000 | 283360000 | 302940000 | 241810000 | 253210000 | 251640000 | O60504 | Vinexin | SORBS3 |
| 1011700000 | 1083600000 | 1158700000 | 967870000 | 952450000 | 965070000 | P98175 | RNA-binding protein 10 | RBM10 |
| 9584400000 | 1.0115E+10 | 1.0025E+10 | 9374100000 | 8924800000 | 8445400000 | P36578 | 60S ribosomal protein L4 | RPL4 |
| 490950000 | 490920000 | 475680000 | 425820000 | 445710000 | 458200000 | P56270 | Myc-associated zinc finger protein | MAZ |
| 2184000000 | 2279100000 | 2209800000 | 2121900000 | 2125300000 | 2078900000 | Q12888 | Tumor suppressor p53-binding protein 1 | TP53BP1 |

| **Decreased proteins from experiment 2** | | | | | | | | |
| --- | --- | --- | --- | --- | --- | --- | --- | --- |
| **Mitotic cells treated with DMSO** | | | **Mitotic cells treated with Act D** | | |  |  |  |
| LFQ intensity | LFQ intensity | LFQ intensity | LFQ intensity | LFQ intensity | LFQ intensity | Protein IDs | Protein names | Gene names |
| 269000000 | 175830000 | 179430000 | 0 | 0 | 0 | A6NKT7 | RanBP2-like and GRIP domain-containing protein 3 | RGPD3 |
| 32731000 | 28486000 | 42640000 | 0 | 0 | 0 | O00411 | DNA-directed RNA polymerase, mitochondrial | POLRMT |
| 73658000 | 98449000 | 69130000 | 0 | 0 | 0 | O14519 | Cyclin-dependent kinase 2-associated protein 1 | CDK2AP1 |
| 14573000 | 17261000 | 26962000 | 0 | 0 | 0 | Q5PSV4 | Breast cancer metastasis-suppressor 1-like protein | BRMS1L |
| 55789000 | 47017000 | 40046000 | 0 | 0 | 0 | Q99547 | M-phase phosphoprotein 6 | MPHOSPH6 |
| 61242000 | 97837000 | 78306000 | 0 | 0 | 0 | Q9ULG6 | Cell cycle progression protein 1 | CCPG1 |
| 224660000 | 129050000 | 88655000 | 34897000 | 8381400 | 0 | Q15773 | Myeloid leukemia factor 2 | MLF2 |
| 77715000 | 38039000 | 37464000 | 18182000 | 0 | 0 | Q9H7D7 | WD repeat-containing protein 26 | WDR26 |
| 102590000 | 41113000 | 104620000 | 25410000 | 0 | 16120000 | Q04837 | Single-stranded DNA-binding protein, mitochondrial | SSBP1 |
| 62887000 | 100220000 | 56359000 | 0 | 40161000 | 0 | Q9Y6V7 | Probable ATP-dependent RNA helicase DDX49 | DDX49 |
| 79859000 | 131300000 | 107660000 | 0 | 64889000 | 0 | Q9UGM6 | Tryptophan--tRNA ligase, mitochondrial | WARS2 |
| 44299000 | 43110000 | 26608000 | 0 | 23761000 | 0 | Q9Y314 | Nitric oxide synthase-interacting protein | NOSIP |
| 48304000 | 70439000 | 40740000 | 0 | 33673000 | 0 | P85037 | Forkhead box protein K1 | FOXK1 |
| 67594000 | 57379000 | 58318000 | 42669000 | 0 | 0 | A8MQ03 | Cysteine-rich tail protein 1 | CYSRT1 |
| 92061000 | 100520000 | 86388000 | 32542000 | 43955000 | 0 | O95453 | Poly(A)-specific ribonuclease PARN | PARN |
| 783870000 | 652570000 | 929000000 | 230740000 | 269410000 | 164760000 | P42696 | RNA-binding protein 34 | RBM34 |
| 278790000 | 208250000 | 213290000 | 147180000 | 49750000 | 0 | P52434 | DNA-directed RNA polymerases I, II, and III subunit RPABC3 | POLR2H |
| 101580000 | 162510000 | 125390000 | 73758000 | 36692000 | 0 | P51665 | 26S proteasome non-ATPase regulatory subunit 7 | PSMD7 |
| 438630000 | 323680000 | 189010000 | 122810000 | 74246000 | 90326000 | P53582 | Methionine aminopeptidase 1 | METAP1 |
| 1703700000 | 2019400000 | 1577600000 | 445870000 | 607260000 | 561210000 | Q9NY93 | Probable ATP-dependent RNA helicase DDX56 | DDX56 |
| 144680000 | 168240000 | 99904000 | 66716000 | 60957000 | 0 | Q6PK04 | Coiled-coil domain-containing protein 137 | CCDC137 |
| 299750000 | 499680000 | 463440000 | 152650000 | 142710000 | 103440000 | Q9NZM5 | Glioma tumor suppressor candidate region gene 2 protein | GLTSCR2 |
| 190860000 | 145340000 | 169800000 | 76100000 | 42987000 | 51168000 | Q9H9Y2 | Ribosome production factor 1 | RPF1 |
| 67456000 | 96164000 | 110840000 | 41492000 | 52062000 | 0 | P48651 | Phosphatidylserine synthase 1 | PTDSS1 |
| 359070000 | 364280000 | 416320000 | 159960000 | 173180000 | 77119000 | Q6RFH5 | WD repeat-containing protein 74 | WDR74 |
| 727940000 | 730200000 | 855870000 | 384980000 | 283770000 | 182350000 | Q9NV06 | DDB1- and CUL4-associated factor 13 | DCAF13 |
| 4901900000 | 5355000000 | 5114700000 | 2217100000 | 1651200000 | 1906700000 | O76021 | Ribosomal L1 domain-containing protein 1 | RSL1D1 |
| 923600000 | 632360000 | 938830000 | 449120000 | 210420000 | 284460000 | Q86U38 | Nucleolar protein 9 | NOP9 |
| 476260000 | 653780000 | 488940000 | 203170000 | 245020000 | 165030000 | Q9NYH9 | U3 small nucleolar RNA-associated protein 6 homolog | UTP6 |
| 1087500000 | 952270000 | 899950000 | 230100000 | 511390000 | 409710000 | Q14692 | Ribosome biogenesis protein BMS1 homolog | BMS1 |
| 682050000 | 857600000 | 701610000 | 288800000 | 287170000 | 309020000 | O15213 | WD repeat-containing protein 46 | WDR46 |
| 940060000 | 1047500000 | 676870000 | 311870000 | 329170000 | 416130000 | Q6DKI1 | 60S ribosomal protein L7-like 1 | RPL7L1 |
| 762120000 | 790110000 | 660650000 | 310140000 | 292750000 | 276890000 | Q9BVJ6;Q5TAP6 | U3 small nucleolar RNA-associated protein 14 homolog A | UTP14A |
| 1539400000 | 2003200000 | 1810000000 | 681220000 | 766980000 | 688420000 | O00541 | Pescadillo homolog | PES1 |
| 796260000 | 588010000 | 598950000 | 196880000 | 340330000 | 258590000 | O95478 | Ribosome biogenesis protein NSA2 homolog | NSA2 |
| 781680000 | 1063800000 | 691210000 | 387270000 | 320490000 | 325550000 | Q9BVI4 | Nucleolar complex protein 4 homolog | NOC4L |
| 225200000 | 214550000 | 206520000 | 82855000 | 96278000 | 99570000 | Q9Y2P8 | RNA 3-terminal phosphate cyclase-like protein | RCL1 |
| 707140000 | 657070000 | 605240000 | 350320000 | 292730000 | 207560000 | P78316 | Nucleolar protein 14 | NOP14 |
| 283240000 | 354450000 | 259630000 | 86207000 | 201090000 | 109840000 | P78346 | Ribonuclease P protein subunit p30 | RPP30 |
| 3959900000 | 3591300000 | 4142700000 | 1801200000 | 1820800000 | 1659600000 | Q8TDN6 | Ribosome biogenesis protein BRX1 homolog | BRIX1 |
| 648670000 | 808430000 | 748070000 | 288070000 | 394970000 | 328080000 | Q9NV31 | U3 small nucleolar ribonucleoprotein protein IMP3 | IMP3 |
| 289870000 | 263300000 | 250960000 | 138430000 | 131060000 | 103690000 | Q8IY37 | Probable ATP-dependent RNA helicase DHX37 | DHX37 |
| 1520000000 | 1517200000 | 1497800000 | 691330000 | 760700000 | 681470000 | Q9BYG3 | MKI67 FHA domain-interacting nucleolar phosphoprotein | NIFK |
| 1814000000 | 2109000000 | 1960500000 | 970840000 | 893000000 | 904880000 | Q15397 | Pumilio domain-containing protein KIAA0020 | KIAA0020 |
| 233700000 | 167850000 | 155010000 | 115880000 | 87016000 | 63631000 | O75683 | Surfeit locus protein 6 | SURF6 |
| 4010800000 | 4066300000 | 3821600000 | 1790300000 | 2261000000 | 1749800000 | Q9BQG0 | Myb-binding protein 1A | MYBBP1A |
| 624060000 | 570630000 | 480830000 | 305120000 | 275410000 | 254820000 | Q13895 | Bystin | BYSL |
| 79983000 | 136620000 | 114140000 | 61086000 | 56594000 | 51568000 | Q15843;E9PL57 | NEDD8 | NEDD8;NEDD8-MDP1 |
| 914690000 | 1198500000 | 1127500000 | 621430000 | 597270000 | 448130000 | Q9H6R4 | Nucleolar protein 6 | NOL6 |
| 752950000 | 926020000 | 671830000 | 339490000 | 475740000 | 401390000 | Q92979 | Ribosomal RNA small subunit methyltransferase NEP1 | EMG1 |
| 1897700000 | 1631600000 | 1563400000 | 874270000 | 1078400000 | 686890000 | Q9GZL7 | Ribosome biogenesis protein WDR12 | WDR12 |
| 885380000 | 1376200000 | 935570000 | 503240000 | 648820000 | 527000000 | O75691 | Small subunit processome component 20 homolog | UTP20 |
| 2059900000 | 2124600000 | 2420400000 | 1016600000 | 1380900000 | 1122700000 | Q96GQ7 | Probable ATP-dependent RNA helicase DDX27 | DDX27 |
| 267080000 | 237620000 | 253980000 | 153970000 | 155400000 | 101480000 | Q9Y4C8 | Probable RNA-binding protein 19 | RBM19 |
| 2886400000 | 2931500000 | 3306900000 | 1765200000 | 1813800000 | 1382600000 | Q5JTH9 | RRP12-like protein | RRP12 |
| 291950000 | 265510000 | 332740000 | 215270000 | 152080000 | 117230000 | Q15024 | Exosome complex component RRP42 | EXOSC7 |
| 1094000000 | 1238400000 | 1246800000 | 642530000 | 818520000 | 579810000 | Q9NW13 | RNA-binding protein 28 | RBM28 |
| 636890000 | 605460000 | 492410000 | 266440000 | 459790000 | 300110000 | Q13823 | Nucleolar GTP-binding protein 2 | GNL2 |
| 2330200000 | 2505200000 | 2831700000 | 1749600000 | 1610100000 | 1228100000 | Q8TDD1 | ATP-dependent RNA helicase DDX54 | DDX54 |
| 2199800000 | 2163200000 | 1942800000 | 1211200000 | 1293600000 | 1270300000 | Q14137 | Ribosome biogenesis protein BOP1 | BOP1 |
| 1.3459E+10 | 1.3909E+10 | 1.3494E+10 | 8063000000 | 8899900000 | 7582400000 | P52272 | Heterogeneous nuclear ribonucleoprotein M | HNRNPM |
| 241730000 | 286910000 | 254920000 | 176760000 | 128870000 | 168340000 | Q9C005 | Protein dpy-30 homolog | DPY30 |
| 1475800000 | 1228700000 | 1329700000 | 718370000 | 1035100000 | 731810000 | Q9H7B2 | Ribosome production factor 2 homolog | RPF2 |
| 1201100000 | 1014900000 | 1136800000 | 816180000 | 714470000 | 538820000 | Q9Y5J1 | U3 small nucleolar RNA-associated protein 18 homolog | UTP18 |
| 1431600000 | 1606600000 | 1371600000 | 591620000 | 954310000 | 1177000000 | Q99848 | Probable rRNA-processing protein EBP2 | EBNA1BP2 |
| 1259700000 | 1147700000 | 1045800000 | 754310000 | 735090000 | 645470000 | Q5QJE6 | Deoxynucleotidyltransferase terminal-interacting protein 2 | DNTTIP2 |
| 348740000 | 269880000 | 356690000 | 247560000 | 205550000 | 151260000 | Q5T280 | Putative methyltransferase C9orf114 | C9orf114 |
| 4062400000 | 4463600000 | 3611300000 | 2804700000 | 2803600000 | 1986800000 | Q14690 | Protein RRP5 homolog | PDCD11 |
| 83267000 | 64722000 | 61177000 | 40688000 | 38526000 | 51980000 | Q9Y3B2 | Exosome complex component CSL4 | EXOSC1 |
| 461110000 | 480640000 | 412580000 | 247780000 | 363540000 | 258620000 | Q96G21 | U3 small nucleolar ribonucleoprotein protein IMP4 | IMP4 |
| 143940000 | 156470000 | 143810000 | 117080000 | 63153000 | 106320000 | P20618 | Proteasome subunit beta type-1 | PSMB1 |
| 575050000 | 679790000 | 457300000 | 381180000 | 334460000 | 391030000 | P08579 | U2 small nuclear ribonucleoprotein B | SNRPB2 |
| 817540000 | 1101300000 | 872230000 | 719400000 | 567640000 | 517170000 | A0A0B4J1V8;Q9NQ55 | Suppressor of SWI4 1 homolog | PPAN-P2RY11;PPAN |
| 4986200000 | 3878300000 | 3895000000 | 2735700000 | 2939500000 | 2597500000 | Q9BZE4 | Nucleolar GTP-binding protein 1 | GTPBP4 |
| 723080000 | 764270000 | 692650000 | 447890000 | 536100000 | 431230000 | Q9NWT1 | p21-activated protein kinase-interacting protein 1 | PAK1IP1 |
| 263620000 | 250670000 | 321050000 | 171740000 | 208010000 | 163010000 | Q9UQ88;P21127 | Cyclin-dependent kinase 11A;Cyclin-dependent kinase 11B | CDK11A;CDK11B |
| 214650000 | 281070000 | 189710000 | 149250000 | 139240000 | 157780000 | Q9Y2L1 | Exosome complex exonuclease RRP44 | DIS3 |
| 1111900000 | 1199400000 | 1059100000 | 919010000 | 669650000 | 635920000 | Q01780 | Exosome component 10 | EXOSC10 |
| 259430000 | 280500000 | 230830000 | 170760000 | 168490000 | 174200000 | Q9BQ75 | Protein CMSS1 | CMSS1 |
| 1223400000 | 933870000 | 1123200000 | 687660000 | 762970000 | 734700000 | Q9BVP2 | Guanine nucleotide-binding protein-like 3 | GNL3 |
| 1269000000 | 984890000 | 1170300000 | 846660000 | 779690000 | 660210000 | Q9H0S4 | Probable ATP-dependent RNA helicase DDX47 | DDX47 |
| 1065000000 | 1189300000 | 1118000000 | 766570000 | 809650000 | 684260000 | Q9GZR7 | ATP-dependent RNA helicase DDX24 | DDX24 |
| 430200000 | 556500000 | 548550000 | 303720000 | 323600000 | 404930000 | Q9NY12 | H/ACA ribonucleoprotein complex subunit 1 | GAR1 |
| 692270000 | 692590000 | 556490000 | 530380000 | 332690000 | 450560000 | Q8NEJ9 | Neuroguidin | NGDN |
| 4393000000 | 4529300000 | 4723100000 | 3352100000 | 3435600000 | 2490400000 | Q9NVP1 | ATP-dependent RNA helicase DDX18 | DDX18 |
| 682930000 | 630070000 | 611320000 | 383890000 | 480580000 | 461040000 | Q15020 | Squamous cell carcinoma antigen recognized by T-cells 3 | SART3 |
| 404850000 | 346900000 | 332140000 | 231900000 | 223450000 | 293710000 | Q13610 | Periodic tryptophan protein 1 homolog | PWP1 |
| 2094200000 | 1982700000 | 2012800000 | 1554500000 | 1526400000 | 1169800000 | Q12788 | Transducin beta-like protein 3 | TBL3 |
| 1408100000 | 1542400000 | 1202200000 | 915110000 | 990880000 | 1016700000 | O00148 | ATP-dependent RNA helicase DDX39A | DDX39A |
| 619770000 | 567980000 | 742520000 | 442280000 | 467620000 | 456270000 | Q9UKS6 | Protein kinase C and casein kinase substrate in neurons protein 3 | PACSIN3 |
| 7822600000 | 6897600000 | 7103300000 | 6105800000 | 5597300000 | 3776800000 | P22087 | rRNA 2-O-methyltransferase fibrillarin | FBL |
| 1931400000 | 1514500000 | 1554100000 | 1215400000 | 1220200000 | 1116500000 | Q9UKD2 | mRNA turnover protein 4 homolog | MRTO4 |
| 113350000 | 132450000 | 130930000 | 79063000 | 89117000 | 101280000 | Q9BTV4 | Transmembrane protein 43 | TMEM43 |
| 943350000 | 1245600000 | 976760000 | 679470000 | 815940000 | 770770000 | Q9H6F5 | Coiled-coil domain-containing protein 86 | CCDC86 |
| 609400000 | 623190000 | 566620000 | 420990000 | 498060000 | 374620000 | O15381 | Nuclear valosin-containing protein-like | NVL |
| 1449700000 | 1118400000 | 1129000000 | 927630000 | 958530000 | 773910000 | Q9NY61 | Protein AATF | AATF |
| 3513000000 | 3595400000 | 4091300000 | 3148000000 | 2107600000 | 2842900000 | P35268 | 60S ribosomal protein L22 | RPL22 |
| 1025800000 | 879430000 | 976760000 | 622570000 | 707300000 | 761050000 | P28288 | ATP-binding cassette sub-family D member 3 | ABCD3 |
| 1052200000 | 1136500000 | 1392800000 | 877390000 | 901270000 | 832250000 | Q9H0H5 | Rac GTPase-activating protein 1 | RACGAP1 |
| 1182400000 | 1055600000 | 1125000000 | 764770000 | 921230000 | 795770000 | O43159 | Ribosomal RNA-processing protein 8 | RRP8 |
| 1641900000 | 2072300000 | 1995300000 | 1464300000 | 1382700000 | 1380900000 | Q13263 | Transcription intermediary factor 1-beta | TRIM28 |
| 3765100000 | 4490600000 | 4053700000 | 3368800000 | 2909600000 | 2856400000 | Q9Y2X3 | Nucleolar protein 58 | NOP58 |
| 1265100000 | 1339400000 | 1402700000 | 1071300000 | 1077500000 | 856490000 | P55795 | Heterogeneous nuclear ribonucleoprotein H2 | HNRNPH2 |
| 2044000000 | 1734900000 | 1772600000 | 1380000000 | 1348200000 | 1444200000 | Q9BQ39 | ATP-dependent RNA helicase DDX50 | DDX50 |
| 299230000 | 352630000 | 341710000 | 245340000 | 277470000 | 230270000 | Q9Y5Q9 | General transcription factor 3C polypeptide 3 | GTF3C3 |
| 3606000000 | 4025500000 | 3640600000 | 3320200000 | 2650600000 | 2729300000 | Q9H0A0 | N-acetyltransferase 10 | NAT10 |
| 2678800000 | 2649900000 | 2725800000 | 1746800000 | 2481200000 | 2038700000 | P46776 | 60S ribosomal protein L27a | RPL27A |
| 4126800000 | 3290100000 | 3760900000 | 2808700000 | 3064000000 | 2855300000 | P52597 | Heterogeneous nuclear ribonucleoprotein F | HNRNPF |
| 1420100000 | 1510700000 | 1417000000 | 1078300000 | 1223600000 | 1093100000 | O15226 | NF-kappa-B-repressing factor | NKRF |
| 3980600000 | 3977800000 | 4114800000 | 2933400000 | 3495700000 | 3057200000 | O43143 | Pre-mRNA-splicing factor ATP-dependent RNA helicase DHX15 | DHX15 |
| 9438300000 | 8277200000 | 8704800000 | 7380400000 | 7468400000 | 6031700000 | Q92841 | Probable ATP-dependent RNA helicase DDX17 | DDX17 |
| 3699300000 | 3726100000 | 3679600000 | 2846400000 | 3066800000 | 2950500000 | Q8IY81 | pre-rRNA processing protein FTSJ3 | FTSJ3 |
| 1632700000 | 1776500000 | 1658100000 | 1167500000 | 1476800000 | 1428300000 | Q12965;O00160 | Unconventional myosin-Ie | MYO1E |
| 1876100000 | 2022700000 | 1927600000 | 1690400000 | 1586100000 | 1414000000 | Q9UNX4 | WD repeat-containing protein 3 | WDR3 |
| 724440000 | 693950000 | 665230000 | 621490000 | 571240000 | 497560000 | Q13206 | Probable ATP-dependent RNA helicase DDX10 | DDX10 |
| 9854900000 | 1.0028E+10 | 1.0775E+10 | 8721700000 | 7614600000 | 8639300000 | Q15233 | Non-POU domain-containing octamer-binding protein | NONO |
| 3254300000 | 3404500000 | 3747700000 | 2864700000 | 2664500000 | 2948800000 | P68363;P68366;A6NHL2;Q9H853 | Tubulin alpha-1B chain;Tubulin alpha-4A chain | TUBA1B;TUBA4A |
| 760640000 | 803540000 | 696390000 | 600180000 | 669960000 | 604500000 | Q5SY16 | Polynucleotide 5-hydroxyl-kinase NOL9 | NOL9 |
| 1715200000 | 1607300000 | 1677200000 | 1338700000 | 1503500000 | 1403700000 | Q9Y3Y2 | Chromatin target of PRMT1 protein | CHTOP |
| 2625400000 | 2594500000 | 2647300000 | 2370100000 | 2333700000 | 2112500000 | O94776 | Metastasis-associated protein MTA2 | MTA2 |
| 9938700000 | 9688500000 | 1.0035E+10 | 8957200000 | 9153200000 | 8233200000 | Q15393 | Splicing factor 3B subunit 3 | SF3B3 |
| 7311600000 | 7503600000 | 7693600000 | 6592200000 | 6805400000 | 6802900000 | P12270 | Nucleoprotein TPR | TPR |
| 2315700000 | 2352900000 | 2244600000 | 2134300000 | 2186800000 | 2007800000 | Q5BKZ1 | DBIRD complex subunit ZNF326 | ZNF326 |
| 3586700000 | 3525400000 | 3299000000 | 3197000000 | 3146800000 | 3234900000 | Q8IX12 | Cell division cycle and apoptosis regulator protein 1 | CCAR1 |

| **Sixty-seven decreased proteins from experiment 1 and 2** | | | | | | | | |
| --- | --- | --- | --- | --- | --- | --- | --- | --- |
| **Mitotic cells treated with DMSO** | | | **Mitotic cells treated with Act D** | | |  |  |  |
| LFQ intensity | LFQ intensity | LFQ intensity | LFQ intensity | LFQ intensity | LFQ intensity | Protein IDs | Protein names | Gene names |
| 118380000 | 88072000 | 89288000 | 0 | 0 | 0 | Q9Y6V7 | Probable ATP-dependent RNA helicase DDX49 | DDX49 |
| 577540000 | 529370000 | 544850000 | 159440000 | 151890000 | 189740000 | Q9Y2P8 | RNA 3-terminal phosphate cyclase-like protein | RCL1 |
| 2198300000 | 1932400000 | 1848300000 | 605360000 | 628090000 | 654030000 | Q9NY93 | Probable ATP-dependent RNA helicase DDX56 | DDX56 |
| 600530000 | 716280000 | 619190000 | 202050000 | 211170000 | 245010000 | Q9NZM5 | Glioma tumor suppressor candidate region gene 2 protein | GLTSCR2 |
| 1653400000 | 1571800000 | 1673400000 | 614120000 | 564540000 | 548990000 | Q9BVJ6;Q5TAP6 | U3 small nucleolar RNA-associated protein 14 homolog A | UTP14A |
| 1.0045E+10 | 9299500000 | 8995500000 | 3765800000 | 3425400000 | 3560400000 | O76021 | Ribosomal L1 domain-containing protein 1 | RSL1D1 |
| 1747600000 | 1619400000 | 1671600000 | 689060000 | 656720000 | 688130000 | Q14692 | Ribosome biogenesis protein BMS1 homolog | BMS1 |
| 1973300000 | 1700400000 | 1788400000 | 802040000 | 744040000 | 674700000 | Q9H6R4 | Nucleolar protein 6 | NOL6 |
| 325010000 | 404460000 | 404600000 | 133040000 | 195260000 | 145050000 | O95453 | Poly(A)-specific ribonuclease PARN | PARN |
| 1036500000 | 1018500000 | 906350000 | 394220000 | 439680000 | 433240000 | Q13895 | Bystin | BYSL |
| 222580000 | 252900000 | 213330000 | 85560000 | 83791000 | 129150000 | Q8IY37 | Probable ATP-dependent RNA helicase DHX37 | DHX37 |
| 893390000 | 728410000 | 759260000 | 331790000 | 327520000 | 388340000 | P42696 | RNA-binding protein 34 | RBM34 |
| 2074200000 | 1774600000 | 2095500000 | 900000000 | 936700000 | 795560000 | Q6DKI1 | 60S ribosomal protein L7-like 1 | RPL7L1 |
| 246080000 | 436960000 | 321180000 | 129800000 | 138770000 | 182020000 | O75683 | Surfeit locus protein 6 | SURF6 |
| 1767300000 | 1635100000 | 1583400000 | 748820000 | 716280000 | 821940000 | O75691 | Small subunit processome component 20 homolog | UTP20 |
| 1138800000 | 1144200000 | 1114800000 | 505970000 | 413280000 | 728980000 | Q9BVI4 | Nucleolar complex protein 4 homolog | NOC4L |
| 5321900000 | 5834300000 | 5319400000 | 2710600000 | 2692600000 | 2903900000 | Q14690 | Protein RRP5 homolog | PDCD11 |
| 1043900000 | 916970000 | 890290000 | 499320000 | 475860000 | 494880000 | P78316 | Nucleolar protein 14 | NOP14 |
| 1663400000 | 1444600000 | 1588800000 | 670460000 | 858270000 | 909170000 | Q9NV06 | DDB1- and CUL4-associated factor 13 | DCAF13 |
| 3076200000 | 2422300000 | 2710000000 | 1626500000 | 1422200000 | 1226700000 | Q9BYG3 | MKI67 FHA domain-interacting nucleolar phosphoprotein | NIFK |
| 1165400000 | 1008600000 | 1174600000 | 555950000 | 458380000 | 759530000 | O95478 | Ribosome biogenesis protein NSA2 homolog | NSA2 |
| 390210000 | 427260000 | 388090000 | 202340000 | 219130000 | 220330000 | Q9BQ75 | Protein CMSS1 | CMSS1 |
| 2469600000 | 2337900000 | 2158800000 | 1336400000 | 910450000 | 1462600000 | Q9BVP2 | Guanine nucleotide-binding protein-like 3 | GNL3 |
| 1254600000 | 1297200000 | 1070100000 | 596110000 | 677790000 | 658060000 | O15213 | WD repeat-containing protein 46 | WDR46 |
| 1096400000 | 940340000 | 825120000 | 591470000 | 465330000 | 477880000 | Q9NYH9 | U3 small nucleolar RNA-associated protein 6 homolog | UTP6 |
| 572780000 | 500550000 | 560720000 | 247750000 | 321520000 | 310790000 | Q9Y4C8 | Probable RNA-binding protein 19 | RBM19 |
| 4080600000 | 4347000000 | 4080700000 | 2558700000 | 2297300000 | 2054200000 | Q15397 | Pumilio domain-containing protein KIAA0020 | KIAA0020 |
| 4651600000 | 5658800000 | 4833300000 | 2816400000 | 2507900000 | 3211800000 | Q5JTH9 | RRP12-like protein | RRP12 |
| 3384700000 | 2722300000 | 2783300000 | 1829200000 | 1603800000 | 1598500000 | O00541 | Pescadillo homolog | PES1 |
| 1747800000 | 1684500000 | 1564000000 | 967300000 | 979990000 | 917140000 | Q9NW13 | RNA-binding protein 28 | RBM28 |
| 486530000 | 403620000 | 404910000 | 228660000 | 244160000 | 272370000 | Q9H9Y2 | Ribosome production factor 1 | RPF1 |
| 917520000 | 885700000 | 780950000 | 453150000 | 482340000 | 562890000 | P53582 | Methionine aminopeptidase 1 | METAP1 |
| 730750000 | 620680000 | 695610000 | 466980000 | 348940000 | 378280000 | Q13610 | Periodic tryptophan protein 1 homolog | PWP1 |
| 5404400000 | 5089600000 | 5828400000 | 3298600000 | 3146100000 | 3079700000 | Q9BQG0 | Myb-binding protein 1A | MYBBP1A |
| 2842500000 | 3313800000 | 2873900000 | 1836000000 | 1703400000 | 1784500000 | Q9GZR7 | ATP-dependent RNA helicase DDX24 | DDX24 |
| 1395500000 | 1337100000 | 1281800000 | 766050000 | 901230000 | 718320000 | Q13823 | Nucleolar GTP-binding protein 2 | GNL2 |
| 985250000 | 899650000 | 833100000 | 582330000 | 535810000 | 523510000 | A0A0B4J1V8;Q9NQ55 | Suppressor of SWI4 1 homolog | PPAN-P2RY11;PPAN |
| 2502600000 | 2411200000 | 2655900000 | 1552500000 | 1525800000 | 1499600000 | Q99848 | Probable rRNA-processing protein EBP2 | EBNA1BP2 |
| 618970000 | 595640000 | 520340000 | 440730000 | 321600000 | 289300000 | Q6RFH5 | WD repeat-containing protein 74 | WDR74 |
| 3913600000 | 3074400000 | 3294200000 | 2013400000 | 1742100000 | 2483000000 | Q96GQ7 | Probable ATP-dependent RNA helicase DDX27 | DDX27 |
| 3451200000 | 3690800000 | 3778800000 | 2297000000 | 2206700000 | 2227000000 | Q9GZL7 | Ribosome biogenesis protein WDR12 | WDR12 |
| 1003500000 | 948730000 | 885250000 | 431680000 | 762530000 | 572590000 | Q86U38 | Nucleolar protein 9 | NOP9 |
| 4384900000 | 4051200000 | 3609000000 | 2437500000 | 2487800000 | 2631600000 | Q14137 | Ribosome biogenesis protein BOP1 | BOP1 |
| 2788900000 | 2839900000 | 3038900000 | 1697400000 | 1786900000 | 2030100000 | Q8TDD1 | ATP-dependent RNA helicase DDX54 | DDX54 |
| 8187100000 | 9793400000 | 8557200000 | 5493100000 | 5783900000 | 5935800000 | Q8TDN6 | Ribosome biogenesis protein BRX1 homolog | BRIX1 |
| 2589000000 | 2544900000 | 2654100000 | 2064100000 | 1589200000 | 1447500000 | Q9H0S4 | Probable ATP-dependent RNA helicase DDX47 | DDX47 |
| 891450000 | 726110000 | 853830000 | 559600000 | 528980000 | 544010000 | Q5T280 | Putative methyltransferase C9orf114 | C9orf114 |
| 2068200000 | 2606700000 | 2341600000 | 1603500000 | 1584400000 | 1521900000 | Q9H7B2 | Ribosome production factor 2 homolog | RPF2 |
| 1641000000 | 1405000000 | 1602600000 | 1086700000 | 899760000 | 1145700000 | O43159 | Ribosomal RNA-processing protein 8 | RRP8 |
| 3859800000 | 4094800000 | 3437200000 | 2743100000 | 2651200000 | 2356000000 | Q9UKD2 | mRNA turnover protein 4 homolog | MRTO4 |
| 1896100000 | 1905500000 | 1675700000 | 1268300000 | 1162000000 | 1318700000 | Q9Y5J1 | U3 small nucleolar RNA-associated protein 18 homolog | UTP18 |
| 237650000 | 244800000 | 199250000 | 160400000 | 148360000 | 160330000 | Q9Y3B2 | Exosome complex component CSL4 | EXOSC1 |
| 6383700000 | 5901500000 | 5800400000 | 4726500000 | 3835900000 | 4108300000 | Q9NVP1 | ATP-dependent RNA helicase DDX18 | DDX18 |
| 647690000 | 571870000 | 535380000 | 387880000 | 379420000 | 462510000 | Q96G21 | U3 small nucleolar ribonucleoprotein protein IMP4 | IMP4 |
| 603140000 | 731970000 | 657560000 | 489540000 | 489820000 | 441090000 | Q15024 | Exosome complex component RRP42 | EXOSC7 |
| 860190000 | 963380000 | 900430000 | 705310000 | 689880000 | 561000000 | Q8NEJ9 | Neuroguidin | NGDN |
| 1478600000 | 1270400000 | 1299000000 | 1121200000 | 958880000 | 837910000 | Q9NWT1 | p21-activated protein kinase-interacting protein 1 | PAK1IP1 |
| 887990000 | 752160000 | 727260000 | 610770000 | 489610000 | 612370000 | Q92979 | Ribosomal RNA small subunit methyltransferase NEP1 | EMG1 |
| 4584100000 | 4583200000 | 4460000000 | 3580000000 | 3366800000 | 3145100000 | Q12788 | Transducin beta-like protein 3 | TBL3 |
| 2166000000 | 2006800000 | 2215300000 | 1681000000 | 1507800000 | 1560000000 | Q5QJE6 | Deoxynucleotidyltransferase terminal-interacting protein 2 | DNTTIP2 |
| 1927800000 | 1856600000 | 1851800000 | 1479600000 | 1382500000 | 1331900000 | Q9NY61 | Protein AATF | AATF |
| 1595900000 | 1439600000 | 1436800000 | 1119400000 | 1039300000 | 1204100000 | Q01780 | Exosome component 10 | EXOSC10 |
| 5828300000 | 5926600000 | 5168400000 | 4485000000 | 4140500000 | 4195500000 | Q9H0A0 | N-acetyltransferase 10 | NAT10 |
| 8591300000 | 7394700000 | 7493600000 | 6148700000 | 5895200000 | 5927100000 | Q9BZE4 | Nucleolar GTP-binding protein 1 | GTPBP4 |
| 1248200000 | 1384800000 | 1225600000 | 1009900000 | 994930000 | 954070000 | Q13206 | Probable ATP-dependent RNA helicase DDX10 | DDX10 |
| 5439300000 | 5363200000 | 6304900000 | 4460200000 | 4525600000 | 4300700000 | Q8IY81 | pre-rRNA processing protein FTSJ3 | FTSJ3 |
| 8369300000 | 9606100000 | 9089900000 | 6773800000 | 7054500000 | 7272600000 | Q9Y2X3 | Nucleolar protein 58 | NOP58 |
