# Supplementary material for: Pre-rRNAs control mitosis by maintaining chromosomal segregation through protecting SMC2 from AURKA-mediated phosphorylation
Source: Cell Death Dis. 2025 Nov 7;16(1):812. doi: 10.1038/s41419-025-08169-9 (PMC12594857; doi:10.1038/s41419-025-08169-9)
Supplement: Supplementary file 7 — Table S2 [file 41419_2025_8169_MOESM7_ESM.docx]

| **Increased proteins from experiment 1** | | | | | | | | |
| --- | --- | --- | --- | --- | --- | --- | --- | --- |
| **Mitotic cells treated with DMSO** | | | **Mitotic cells treated with Act D** | | |  |  |  |
| LFQ intensity | LFQ intensity | LFQ intensity | LFQ intensity | LFQ intensity | LFQ intensity | Protein IDs | Protein names | Gene names |
| 3717500000 | 3751500000 | 3869600000 | 4029900000 | 3914000000 | 4085800000 | Q6PJT7 | Zinc finger CCCH domain-containing protein 14 | ZC3H14 |
| 1034800000 | 1019200000 | 1004400000 | 1124400000 | 1085800000 | 1056400000 | Q9NVI7 | ATPase family AAA domain-containing protein 3A | ATAD3A |
| 1155800000 | 1113300000 | 1171400000 | 1249900000 | 1266600000 | 1191400000 | Q86W42 | THO complex subunit 6 homolog | THOC6 |
| 2453300000 | 2502600000 | 2399900000 | 2774300000 | 2552800000 | 2733700000 | Q14151 | Scaffold attachment factor B2 | SAFB2 |
| 1.846E+10 | 1.8157E+10 | 1.9589E+10 | 2.1279E+10 | 2.0233E+10 | 2.0421E+10 | O00571;O15523;Q9NQI0 | ATP-dependent RNA helicase DDX3X;ATP-dependent RNA helicase DDX3Y | DDX3X;DDX3Y |
| 9.5948E+10 | 9.2255E+10 | 9.4926E+10 | 1.0379E+11 | 1.0141E+11 | 1.0726E+11 | Q6FI13;Q16777 | Histone H2A type 2-A;Histone H2A type 2-C | HIST2H2AA3 |
| 278170000 | 310340000 | 298250000 | 319080000 | 322950000 | 337220000 | Q96HW7 | Integrator complex subunit 4 | INTS4 |
| 292480000 | 286430000 | 268210000 | 319990000 | 308750000 | 307070000 | P67775;P62714;P60510 | Serine/threonine-protein phosphatase 2A catalytic subunit alpha isoform | PPP2CA;PPP2CB |
| 557950000 | 538300000 | 553440000 | 639400000 | 581380000 | 601840000 | Q96L91 | E1A-binding protein p400 | EP400 |
| 5055100000 | 5003400000 | 5232700000 | 5787100000 | 5680400000 | 5547900000 | Q9ULW0 | Targeting protein for Xklp2 | TPX2 |
| 561430000 | 586120000 | 560380000 | 604420000 | 671800000 | 629360000 | O60684;O15131 | Importin subunit alpha-7;Importin subunit alpha-6 | KPNA6;KPNA5 |
| 1.4215E+10 | 1.4196E+10 | 1.3861E+10 | 1.6001E+10 | 1.5506E+10 | 1.5754E+10 | Q9UQ35 | Serine/arginine repetitive matrix protein 2 | SRRM2 |
| 2850500000 | 2596200000 | 2869600000 | 3143400000 | 3198200000 | 2980000000 | P46781 | 40S ribosomal protein S9 | RPS9 |
| 8687300000 | 8839900000 | 8452900000 | 1.0068E+10 | 9116200000 | 1.0115E+10 | O60264;P28370 | SWI/SNF-related matrix-associated actin-dependent regulator of chromatin subfamily A member 5 | SMARCA5 |
| 2.9074E+10 | 2.9794E+10 | 3.19E+10 | 3.5073E+10 | 3.4306E+10 | 3.3082E+10 | P68431;Q16695;Q6NXT2 | Histone H3.1;Histone H3.1t;Histone H3.3C | HIST1H3A;HIST3H3 |
| 802210000 | 699140000 | 756050000 | 858430000 | 819440000 | 881840000 | P41223 | Protein BUD31 homolog | BUD31 |
| 213840000 | 215800000 | 199870000 | 234690000 | 250660000 | 231640000 | Q9C0C2 | 182 kDa tankyrase-1-binding protein | TNKS1BP1 |
| 1278600000 | 1448000000 | 1321100000 | 1582300000 | 1557500000 | 1481000000 | Q7L2E3 | Putative ATP-dependent RNA helicase DHX30 | DHX30 |
| 462890000 | 483480000 | 429060000 | 551760000 | 512430000 | 506330000 | Q8NCA5 | Protein FAM98A | FAM98A |
| 60587000 | 59566000 | 56744000 | 66359000 | 63722000 | 72640000 | Q9Y2S0 | DNA-directed RNA polymerases I and III subunit RPAC2 | POLR1D |
| 5231500000 | 4665800000 | 4881400000 | 5683800000 | 5657100000 | 5676100000 | Q9UKM9 | RNA-binding protein Raly | RALY |
| 611460000 | 568120000 | 671250000 | 694030000 | 710060000 | 738880000 | O95785 | Protein Wiz | WIZ |
| 232850000 | 200860000 | 228130000 | 267550000 | 248510000 | 252970000 | Q8NDT2 | Putative RNA-binding protein 15B | RBM15B |
| 758820000 | 807850000 | 851650000 | 882210000 | 970600000 | 958700000 | Q8NEY8 | Periphilin-1 | PPHLN1 |
| 1378800000 | 1554800000 | 1557000000 | 1631900000 | 1752200000 | 1840900000 | Q5SY16 | Polynucleotide 5-hydroxyl-kinase NOL9 | NOL9 |
| 698970000 | 658360000 | 680160000 | 786560000 | 744250000 | 853450000 | Q96J01 | THO complex subunit 3 | THOC3 |
| 6194900000 | 6009800000 | 5469400000 | 7135500000 | 6471400000 | 7092700000 | P18583 | Protein SON | SON |
| 1.0402E+10 | 9555600000 | 9856900000 | 1.2225E+10 | 1.1225E+10 | 1.164E+10 | Q12905 | Interleukin enhancer-binding factor 2 | ILF2 |
| 245860000 | 260730000 | 280740000 | 312990000 | 312330000 | 301620000 | Q9GZS3 | WD repeat-containing protein 61 | WDR61 |
| 416100000 | 403980000 | 382580000 | 504520000 | 455740000 | 458710000 | Q5T3J3 | Ligand-dependent nuclear receptor-interacting factor 1 | LRIF1 |
| 1584400000 | 1527400000 | 1783900000 | 2070800000 | 1934600000 | 1806000000 | Q6UN15;A0A0B4J203 | Pre-mRNA 3-end-processing factor FIP1 | FIP1L1 |
| 3.6301E+10 | 3.9127E+10 | 3.4587E+10 | 4.7218E+10 | 4.2964E+10 | 4.0959E+10 | P08670;P17661;P41219;P14136 | Vimentin | VIM |
| 476270000 | 531120000 | 510790000 | 645360000 | 609730000 | 554890000 | Q9BTX1 | Nucleoporin NDC1 | NDC1 |
| 2373000000 | 2130000000 | 2417800000 | 2866600000 | 2642400000 | 2742400000 | P84090 | Enhancer of rudimentary homolog | ERH |
| 839120000 | 744400000 | 933520000 | 985120000 | 1065200000 | 979270000 | Q8NFW8 | N-acylneuraminate cytidylyltransferase | CMAS |
| 1842200000 | 1653000000 | 1978900000 | 2318600000 | 2151100000 | 2129700000 | Q16630 | Cleavage and polyadenylation specificity factor subunit 6 | CPSF6 |
| 703370000 | 830680000 | 721320000 | 913300000 | 896300000 | 913320000 | Q9NZI8;Q9Y6M1 | Insulin-like growth factor 2 mRNA-binding protein 1 | IGF2BP1 |
| 4291800000 | 4896800000 | 4726700000 | 5564300000 | 5683500000 | 5758400000 | Q9UKV3 | Apoptotic chromatin condensation inducer in the nucleus | ACIN1 |
| 163180000 | 170190000 | 169810000 | 180660000 | 225910000 | 208550000 | Q9NUU7 | ATP-dependent RNA helicase DDX19A | DDX19A |
| 238290000 | 193900000 | 228450000 | 258740000 | 267810000 | 283190000 | Q9HCE1 | Putative helicase MOV-10 | MOV10 |
| 5784300000 | 6031100000 | 6231000000 | 7255700000 | 7255500000 | 7658000000 | O60506 | Heterogeneous nuclear ribonucleoprotein Q | SYNCRIP |
| 198680000 | 206730000 | 196530000 | 265430000 | 237940000 | 236710000 | Q5TGY3 | AT-hook DNA-binding motif-containing protein 1 | AHDC1 |
| 507900000 | 517030000 | 482270000 | 590630000 | 629270000 | 639230000 | Q9NZB2;Q5T035 | Constitutive coactivator of PPAR-gamma-like protein 1 | FAM120A |
| 274780000 | 261760000 | 230040000 | 284020000 | 334900000 | 331210000 | F8WAN1;Q69YQ0 | Cytospin-A | SPECC1L-ADORA2A |
| 396130000 | 450480000 | 421010000 | 557390000 | 492740000 | 522210000 | P51114 | Fragile X mental retardation syndrome-related protein 1 | FXR1 |
| 3691400000 | 3853600000 | 3897400000 | 4411000000 | 5029100000 | 4763800000 | P11940;Q9H361;Q4VXU2 | Polyadenylate-binding protein 1 | PABPC1 |
| 189720000 | 197260000 | 161310000 | 214550000 | 219980000 | 247310000 | Q9UPN9 | E3 ubiquitin-protein ligase TRIM33 | TRIM33 |
| 926710000 | 878330000 | 872640000 | 1174100000 | 1006200000 | 1164400000 | P27694 | Replication protein A 70 kDa DNA-binding subunit | RPA1 |
| 622560000 | 581390000 | 591310000 | 821630000 | 746080000 | 675320000 | O14965 | Aurora kinase A | AURKA |
| 70035000 | 65782000 | 67684000 | 78781000 | 81153000 | 96623000 | Q15695;Q15696 | U2 small nuclear ribonucleoprotein auxiliary factor 35 kDa subunit-related protein 1 | ZRSR1;ZRSR2 |
| 712960000 | 642670000 | 736050000 | 874600000 | 922630000 | 845060000 | Q86V48 | Leucine zipper protein 1 | LUZP1 |
| 316300000 | 347720000 | 267010000 | 401090000 | 369980000 | 416350000 | P25440 | Bromodomain-containing protein 2 | BRD2 |
| 221180000 | 195950000 | 185990000 | 231420000 | 266900000 | 274690000 | Q15291 | Retinoblastoma-binding protein 5 | RBBP5 |
| 1960500000 | 1571500000 | 1546700000 | 2044200000 | 2215600000 | 2258400000 | P26641 | Elongation factor 1-gamma | EEF1G |
| 629360000 | 509540000 | 619490000 | 702910000 | 801660000 | 754040000 | P63208 | S-phase kinase-associated protein 1 | SKP1 |
| 889280000 | 834600000 | 824830000 | 1141500000 | 942620000 | 1240100000 | O95251 | Histone acetyltransferase KAT7 | KAT7 |
| 3529900000 | 3057100000 | 2831300000 | 4562900000 | 3617500000 | 4163000000 | Q6PL18 | ATPase family AAA domain-containing protein 2 | ATAD2 |
| 280400000 | 249030000 | 236630000 | 336470000 | 298070000 | 371670000 | Q8IY18 | Structural maintenance of chromosomes protein 5 | SMC5 |
| 516270000 | 447630000 | 378310000 | 588570000 | 583320000 | 595890000 | Q9UPN3 | Microtubule-actin cross-linking factor 1, isoforms 1/2/3/5 | MACF1 |
| 420490000 | 361480000 | 383710000 | 505750000 | 486190000 | 548300000 | Q96SB8 | Structural maintenance of chromosomes protein 6 | SMC6 |
| 70089000 | 75819000 | 79351000 | 98006000 | 96178000 | 103870000 | O14908;Q8TF64 | PDZ domain-containing protein GIPC1 | GIPC1 |
| 806470000 | 774130000 | 663940000 | 896840000 | 988950000 | 1088000000 | Q05519 | Serine/arginine-rich splicing factor 11 | SRSF11 |
| 558560000 | 515220000 | 452330000 | 657000000 | 719750000 | 661770000 | O00629 | Importin subunit alpha-3 | KPNA4 |
| 85645000 | 96170000 | 101740000 | 120320000 | 145510000 | 117660000 | Q9NUA8 | Zinc finger and BTB domain-containing protein 40 | ZBTB40 |
| 425000000 | 391670000 | 411910000 | 560400000 | 585300000 | 519270000 | O15392 | Baculoviral IAP repeat-containing protein 5 | BIRC5 |
| 1001200000 | 1078300000 | 1091500000 | 1402000000 | 1340000000 | 1585000000 | Q86UP2;Q9Y4F3 | Kinectin | KTN1 |
| 181350000 | 155940000 | 185160000 | 246880000 | 206830000 | 272800000 | P05549;Q6VUC0;Q92481 | Transcription factor AP-2-alpha | TFAP2A;TFAP2E |
| 68983000 | 60012000 | 64307000 | 96933000 | 87553000 | 85920000 | Q96EZ8 | Microspherule protein 1 | MCRS1 |
| 126480000 | 111460000 | 126370000 | 150890000 | 171530000 | 194490000 | Q9Y6X3 | MAU2 chromatid cohesion factor homolog | MAU2 |
| 209650000 | 173420000 | 149460000 | 278480000 | 240320000 | 245870000 | Q6PKG0;Q659C4 | La-related protein 1 | LARP1 |
| 164500000 | 105680000 | 168090000 | 230150000 | 216840000 | 226910000 | Q6PIJ6 | F-box only protein 38 | FBXO38 |
| 311700000 | 240790000 | 265440000 | 404010000 | 352840000 | 514800000 | Q06481 | Amyloid-like protein 2 | APLP2 |
| 55035000 | 45600000 | 54451000 | 68272000 | 94257000 | 96600000 | P41214 | Eukaryotic translation initiation factor 2D | EIF2D |
| 54415000 | 33877000 | 40167000 | 82119000 | 66622000 | 67969000 | P08758 | Annexin A5 | ANXA5 |
| 507010000 | 478420000 | 513960000 | 917110000 | 833760000 | 978110000 | Q9NVN8 | Guanine nucleotide-binding protein-like 3-like protein | GNL3L |
| 120730000 | 167970000 | 191860000 | 404440000 | 218810000 | 362430000 | Q92974 | Rho guanine nucleotide exchange factor 2 | ARHGEF2 |
| 68436000 | 77337000 | 95332000 | 148530000 | 176980000 | 178630000 | Q13492;O60641 | Phosphatidylinositol-binding clathrin assembly protein | PICALM |
| 0 | 81585000 | 0 | 128630000 | 123770000 | 94140000 | Q96TA2 | ATP-dependent zinc metalloprotease YME1L1 | YME1L1 |
| 0 | 0 | 74299000 | 109700000 | 97147000 | 112990000 | O15347 | High mobility group protein B3 | HMGB3 |
| 24235000 | 0 | 0 | 44407000 | 32747000 | 43759000 | P50395;P31150 | Rab GDP dissociation inhibitor beta | GDI2 |
| 0 | 0 | 8404800 | 19806000 | 10576000 | 11831000 | Q9NVM6 | DnaJ homolog subfamily C member 17 | DNAJC17 |
| 0 | 34351000 | 0 | 76900000 | 81333000 | 55192000 | P43487 | Ran-specific GTPase-activating protein | RANBP1 |
| 0 | 0 | 0 | 43647000 | 42632000 | 51583000 | Q13347 | Eukaryotic translation initiation factor 3 subunit I | EIF3I |
| 0 | 0 | 0 | 17234000 | 18387000 | 16011000 | Q6R327 | Rapamycin-insensitive companion of mTOR | RICTOR |
| 0 | 0 | 0 | 52371000 | 57558000 | 33210000 | Q8TEQ6 | Gem-associated protein 5 | GEMIN5 |
| 0 | 0 | 0 | 48029000 | 31515000 | 32832000 | Q96KM6 | Zinc finger protein 512B | ZNF512B |
| 0 | 0 | 0 | 48391000 | 49708000 | 57860000 | Q99583 | Max-binding protein MNT | MNT |
| 0 | 0 | 0 | 31438000 | 16195000 | 19127000 | Q9BQI6 | Ankyrin repeat domain-containing protein 32 | ANKRD32 |

| **Increased proteins from experiment 2** | | | | | | | | |
| --- | --- | --- | --- | --- | --- | --- | --- | --- |
| **Mitotic cells treated with DMSO** | | | **Mitotic cells treated with Act D** | | |  |  |  |
| LFQ intensity | LFQ intensity | LFQ intensity | LFQ intensity | LFQ intensity | LFQ intensity | Protein IDs | Protein names | Gene names |
| 7.526E+10 | 7.3368E+10 | 7.4615E+10 | 8.2624E+10 | 8.0674E+10 | 7.8687E+10 | Q99879;Q99877;Q93079 | Histone H2B type 1-M;Histone H2B type 1-N;Histone H2B type 1-H | HIST1H2BM |
| 2207000000 | 2211900000 | 1991800000 | 2367400000 | 2420500000 | 2325300000 | Q8WYP5 | Protein ELYS | AHCTF1 |
| 1.0298E+11 | 1.0404E+11 | 1.0071E+11 | 1.2174E+11 | 1.1304E+11 | 1.0798E+11 | P62805 | Histone H4 | HIST1H4A |
| 261440000 | 291070000 | 276370000 | 295840000 | 324300000 | 308830000 | Q69YH5 | Cell division cycle-associated protein 2 | CDCA2 |
| 189310000 | 203190000 | 199600000 | 231380000 | 231910000 | 220130000 | A8CG34;A6NF01;Q6PJE2 | Nuclear envelope pore membrane protein POM 121C | POM121C |
| 5265900000 | 5602000000 | 5870200000 | 6359900000 | 6383400000 | 7006500000 | P21333 | Filamin-A | FLNA |
| 106130000 | 102310000 | 89056000 | 116450000 | 122670000 | 121300000 | Q9C0B5 | Palmitoyltransferase ZDHHC5 | ZDHHC5 |
| 2.1791E+10 | 1.9563E+10 | 2.0693E+10 | 2.4817E+10 | 2.6886E+10 | 2.3802E+10 | Q09666 | Neuroblast differentiation-associated protein AHNAK | AHNAK |
| 3488600000 | 3192900000 | 3135100000 | 3788600000 | 3879200000 | 4293500000 | Q99459 | Cell division cycle 5-like protein | CDC5L |
| 1458400000 | 1314200000 | 1248200000 | 1696200000 | 1759200000 | 1537000000 | O75152;A0A1B0GUI2 | Zinc finger CCCH domain-containing protein 11A | ZC3H11A |
| 434680000 | 417890000 | 423920000 | 580220000 | 529540000 | 481060000 | Q9BW19 | Kinesin-like protein KIFC1 | KIFC1 |
| 2715900000 | 2555800000 | 2340700000 | 2867400000 | 3446300000 | 3174500000 | Q9ULW0 | Targeting protein for Xklp2 | TPX2 |
| 219150000 | 221360000 | 213980000 | 291200000 | 281690000 | 246870000 | Q9BR76 | Coronin-1B | CORO1B |
| 133000000 | 101720000 | 117210000 | 149020000 | 155100000 | 139490000 | O00425 | Insulin-like growth factor 2 mRNA-binding protein 3 | IGF2BP3 |
| 552100000 | 678870000 | 512110000 | 716380000 | 765500000 | 715370000 | Q9Y2U8 | Inner nuclear membrane protein Man1 | LEMD3 |
| 7860600000 | 6890300000 | 5931800000 | 8899500000 | 8051500000 | 9350500000 | Q9UMS4 | Pre-mRNA-processing factor 19 | PRPF19 |
| 1088400000 | 987120000 | 960150000 | 1346400000 | 1373300000 | 1156200000 | Q14978 | Nucleolar and coiled-body phosphoprotein 1 | NOLC1 |
| 254740000 | 268300000 | 242280000 | 329610000 | 334790000 | 316460000 | Q03164 | Histone-lysine N-methyltransferase 2A;MLL cleavage product N320 | KMT2A |
| 2109100000 | 2528000000 | 2660800000 | 3288600000 | 3127500000 | 2954800000 | P27105 | Erythrocyte band 7 integral membrane protein | STOM |
| 1.4546E+10 | 1.415E+10 | 1.4157E+10 | 1.9408E+10 | 1.9549E+10 | 1.6589E+10 | P11388 | DNA topoisomerase 2-alpha | TOP2A |
| 202740000 | 169800000 | 165480000 | 244970000 | 233180000 | 227780000 | Q9NSD9 | Phenylalanine--tRNA ligase beta subunit | FARSB |
| 232520000 | 251310000 | 254820000 | 332590000 | 341180000 | 296580000 | Q9NRL2 | Bromodomain adjacent to zinc finger domain protein 1A | BAZ1A |
| 131690000 | 110590000 | 103140000 | 148840000 | 146210000 | 159000000 | Q9Y6U3 | Adseverin | SCIN |
| 467180000 | 460360000 | 599010000 | 717590000 | 662640000 | 628880000 | Q92614 | Unconventional myosin-XVIIIa | MYO18A |
| 1354000000 | 1092700000 | 1258200000 | 1463200000 | 1666100000 | 1750600000 | Q9BWF3 | RNA-binding protein 4 | RBM4 |
| 1560600000 | 1652600000 | 1649500000 | 1963700000 | 2470900000 | 1974800000 | Q96T37 | Putative RNA-binding protein 15 | RBM15 |
| 398070000 | 422310000 | 489210000 | 543480000 | 555520000 | 641530000 | Q13501 | Sequestosome-1 | SQSTM1 |
| 935740000 | 830390000 | 1034900000 | 1198700000 | 1352900000 | 1180500000 | Q02880 | DNA topoisomerase 2-beta | TOP2B |
| 530250000 | 450220000 | 458990000 | 546520000 | 662190000 | 713080000 | Q14247 | Src substrate cortactin | CTTN |
| 32674000 | 27009000 | 35988000 | 48293000 | 40498000 | 39496000 | P33176 | Kinesin-1 heavy chain | KIF5B |
| 324100000 | 335210000 | 345420000 | 428900000 | 510180000 | 411640000 | Q9BZJ0 | Crooked neck-like protein 1 | CRNKL1 |
| 958580000 | 1065800000 | 1114300000 | 1303400000 | 1381000000 | 1537000000 | P60660;P14649 | Myosin light polypeptide 6 | MYL6 |
| 236640000 | 289330000 | 280750000 | 371780000 | 320870000 | 406100000 | Q07157 | Tight junction protein ZO-1 | TJP1 |
| 1646900000 | 1408100000 | 1323200000 | 2020700000 | 2249900000 | 1732400000 | O60841 | Eukaryotic translation initiation factor 5B | EIF5B |
| 2062700000 | 2385700000 | 2417600000 | 3543700000 | 3016000000 | 2882200000 | P35579 | Myosin-9 | MYH9 |
| 882180000 | 914300000 | 800680000 | 1285100000 | 1226100000 | 1087000000 | O75475 | PC4 and SFRS1-interacting protein | PSIP1 |
| 178820000 | 233030000 | 202440000 | 313480000 | 271430000 | 267990000 | P25205 | DNA replication licensing factor MCM3 | MCM3 |
| 255720000 | 319850000 | 250660000 | 416780000 | 330120000 | 400920000 | P30153 | Serine/threonine-protein phosphatase 2A 65 kDa regulatory subunit A alpha isoform | PPP2R1A |
| 136110000 | 163440000 | 160670000 | 216910000 | 227100000 | 200830000 | P51608 | Methyl-CpG-binding protein 2 | MECP2 |
| 119730000 | 106090000 | 114920000 | 161330000 | 171090000 | 146240000 | Q15910 | Histone-lysine N-methyltransferase EZH2 | EZH2 |
| 371260000 | 273210000 | 338390000 | 435490000 | 514360000 | 431000000 | P16989 | Y-box-binding protein 3 | YBX3 |
| 404730000 | 421740000 | 389000000 | 528200000 | 630480000 | 562480000 | P39748 | Flap endonuclease 1 | FEN1 |
| 938880000 | 983240000 | 901050000 | 1299700000 | 1449800000 | 1267900000 | O95235 | Kinesin-like protein KIF20A | KIF20A |
| 422940000 | 408470000 | 484560000 | 609050000 | 750730000 | 533300000 | O14965 | Aurora kinase A | AURKA |
| 218430000 | 229290000 | 236300000 | 330250000 | 368750000 | 285490000 | Q9NQG5 | Regulation of nuclear pre-mRNA domain-containing protein 1B | RPRD1B |
| 1.0937E+10 | 9161600000 | 9858500000 | 1.4331E+10 | 1.5489E+10 | 1.398E+10 | P16401 | Histone H1.5 | HIST1H1B |
| 208170000 | 296000000 | 196680000 | 369160000 | 323290000 | 341970000 | O94842;O15405 | TOX high mobility group box family member 4 | TOX4 |
| 676810000 | 527040000 | 637610000 | 818240000 | 1057500000 | 891340000 | Q13310;P0CB38 | Polyadenylate-binding protein 4 | PABPC4 |
| 160860000 | 190880000 | 200870000 | 312620000 | 290340000 | 227990000 | P78347 | General transcription factor II-I | GTF2I |
| 463810000 | 547850000 | 499340000 | 684970000 | 766550000 | 846040000 | P29692 | Elongation factor 1-delta | EEF1D |
| 367850000 | 510690000 | 409220000 | 708680000 | 557420000 | 704830000 | P04844 | Dolichyl-diphosphooligosaccharide--protein glycosyltransferase subunit 2 | RPN2 |
| 90173000 | 70267000 | 65568000 | 123760000 | 105750000 | 119330000 | Q96ME7 | Zinc finger protein 512 | ZNF512 |
| 221980000 | 190250000 | 301630000 | 401560000 | 322100000 | 385530000 | P35250 | Replication factor C subunit 2 | RFC2 |
| 534550000 | 639200000 | 596490000 | 892420000 | 779870000 | 1082700000 | P04843 | Dolichyl-diphosphooligosaccharide--protein glycosyltransferase subunit 1 | RPN1 |
| 182380000 | 186450000 | 232820000 | 357130000 | 277310000 | 324440000 | P35251 | Replication factor C subunit 1 | RFC1 |
| 1123200000 | 1344000000 | 1187300000 | 1896800000 | 1767100000 | 2261800000 | P26641 | Elongation factor 1-gamma | EEF1G |
| 101370000 | 107010000 | 110970000 | 183100000 | 156660000 | 189250000 | Q14011 | Cold-inducible RNA-binding protein | CIRBP |
| 1724800000 | 1203300000 | 1188200000 | 1941300000 | 2409300000 | 2522700000 | P62081 | 40S ribosomal protein S7 | RPS7 |
| 14593000 | 12334000 | 13168000 | 24085000 | 17312000 | 27946000 | O94826 | Mitochondrial import receptor subunit TOM70 | TOMM70A |
| 319530000 | 240470000 | 151870000 | 454230000 | 420410000 | 362440000 | Q9NVN8 | Guanine nucleotide-binding protein-like 3-like protein | GNL3L |
| 154150000 | 128210000 | 150840000 | 253790000 | 277770000 | 225480000 | P23921 | Ribonucleoside-diphosphate reductase large subunit | RRM1 |
| 36523000 | 37446000 | 58081000 | 75151000 | 66950000 | 89825000 | P16615;O14983 | Sarcoplasmic/endoplasmic reticulum calcium ATPase 2 | ATP2A2 |
| 243150000 | 104950000 | 144710000 | 298350000 | 271520000 | 300610000 | O14880 | Microsomal glutathione S-transferase 3 | MGST3 |
| 135800000 | 140870000 | 121180000 | 236620000 | 241780000 | 234970000 | Q02809 | Procollagen-lysine,2-oxoglutarate 5-dioxygenase 1 | PLOD1 |
| 68411000 | 61316000 | 62048000 | 125980000 | 90032000 | 128040000 | Q8WUM4 | Programmed cell death 6-interacting protein | PDCD6IP |
| 94140000 | 84373000 | 88353000 | 124780000 | 149490000 | 204930000 | Q9Y3F4 | Serine-threonine kinase receptor-associated protein | STRAP |
| 78345000 | 137480000 | 93499000 | 186780000 | 225420000 | 144300000 | O00488 | Zinc finger protein 593 | ZNF593 |
| 436710000 | 415620000 | 286300000 | 622640000 | 693220000 | 733350000 | Q9UQ80;H0YIN7 | Proliferation-associated protein 2G4 | PA2G4 |
| 47842000 | 75472000 | 81262000 | 114270000 | 121560000 | 132650000 | O75477 | Erlin-1 | ERLIN1 |
| 397680000 | 372430000 | 380330000 | 690840000 | 673280000 | 709170000 | Q96P16 | Regulation of nuclear pre-mRNA domain-containing protein 1A | RPRD1A |
| 174770000 | 121040000 | 132760000 | 246800000 | 273880000 | 282960000 | Q02539 | Histone H1.1 | HIST1H1A |
| 176030000 | 178500000 | 245640000 | 478710000 | 457270000 | 285950000 | P36954 | DNA-directed RNA polymerase II subunit RPB9 | POLR2I |
| 63604000 | 62028000 | 53558000 | 109000000 | 139820000 | 133040000 | Q14694 | Ubiquitin carboxyl-terminal hydrolase 10 | USP10 |
| 91780000 | 122560000 | 100800000 | 244020000 | 305160000 | 161700000 | P63208 | S-phase kinase-associated protein 1 | SKP1 |
| 25561000 | 37531000 | 26021000 | 58190000 | 74812000 | 77928000 | Q9Y6I4 | Ubiquitin carboxyl-terminal hydrolase 3 | USP3 |
| 0 | 67510000 | 0 | 75250000 | 90738000 | 96926000 | Q96AE4 | Far upstream element-binding protein 1 | FUBP1 |
| 0 | 0 | 61696000 | 81355000 | 86503000 | 75099000 | O95391 | Pre-mRNA-splicing factor SLU7 | SLU7 |
| 0 | 0 | 47244000 | 74747000 | 57104000 | 59104000 | Q96CU9 | FAD-dependent oxidoreductase domain-containing protein 1 | FOXRED1 |
| 0 | 37482000 | 0 | 42177000 | 102150000 | 80164000 | Q8WX92 | Negative elongation factor B | NELFB |
| 0 | 0 | 18686000 | 54892000 | 57576000 | 34446000 | Q9Y295 | Developmentally-regulated GTP-binding protein 1 | DRG1 |
| 0 | 21943000 | 0 | 55030000 | 59846000 | 72448000 | Q9UHA4 | Ragulator complex protein LAMTOR3 | LAMTOR3 |
| 0 | 0 | 0 | 41649000 | 55737000 | 46771000 | P62834;P61224 | Ras-related protein Rap-1A;Ras-related protein Rap-1b | RAP1A;RAP1B |
| 0 | 0 | 0 | 42475000 | 25761000 | 23354000 | Q8NAP3;Q9P1Z0 | Zinc finger and BTB domain-containing protein 38 | ZBTB38 |
| 0 | 0 | 0 | 9507800 | 11062000 | 18126000 | Q9BRP8 | Partner of Y14 and mago | WIBG |

| **Increased proteins from experiment 1 and 2** | | | | | | | | |
| --- | --- | --- | --- | --- | --- | --- | --- | --- |
| **Mitotic cells treated with DMSO** | | | **Mitotic cells treated with Act D** | | |  |  |  |
| LFQ intensity | LFQ intensity | LFQ intensity | LFQ intensity | LFQ intensity | LFQ intensity | Protein IDs | Protein names | Gene names |
| 5055100000 | 5003400000 | 5232700000 | 5787100000 | 5680400000 | 5547900000 | Q9ULW0 | Targeting protein for Xklp2 | TPX2 |
| 622560000 | 581390000 | 591310000 | 821630000 | 746080000 | 675320000 | O14965 | Aurora kinase A | AURKA |
| 1960500000 | 1571500000 | 1546700000 | 2044200000 | 2215600000 | 2258400000 | P26641 | Elongation factor 1-gamma | EEF1G |
| 629360000 | 509540000 | 619490000 | 702910000 | 801660000 | 754040000 | P63208 | S-phase kinase-associated protein 1 | SKP1 |
| 507010000 | 478420000 | 513960000 | 917110000 | 833760000 | 978110000 | Q9NVN8 | Guanine nucleotide-binding protein-like 3-like protein | GNL3L |
