# Supplementary material for: Pre-rRNAs control mitosis by maintaining chromosomal segregation through protecting SMC2 from AURKA-mediated phosphorylation
Source: Cell Death Dis. 2025 Nov 7;16(1):812. doi: 10.1038/s41419-025-08169-9 (PMC12594857; doi:10.1038/s41419-025-08169-9)
Supplement: Supplementary file 8 — Table S3 [file 41419_2025_8169_MOESM8_ESM.docx]

| **AURKA-binding proteins in chromosomal region from mitotic cells treated with Act D** | | |
| --- | --- | --- |
| **Protein IDs** | **Protein names** | **Gene names** |
| P19105;O14950;P24844 | Myosin regulatory light chain 12A;Myosin regulatory light chain 12B | MYL12A;MYL12B |
| O43175 | D-3-phosphoglycerate dehydrogenase | PHGDH |
| O43823 | A-kinase anchor protein 8 | AKAP8 |
| O60287 | Nucleolar pre-ribosomal-associated protein 1 | URB1 |
| O60318 | Germinal-center associated nuclear protein | MCM3AP |
| O60504 | Vinexin | SORBS3 |
| O95391 | Pre-mRNA-splicing factor SLU7 | SLU7 |
| O95425 | Supervillin | SVIL |
| O95602 | DNA-directed RNA polymerase I subunit RPA1 | POLR1A |
| P22314 | Ubiquitin-like modifier-activating enzyme 1 | UBA1 |
| P29375 | Lysine-specific demethylase 5A | KDM5A |
| P47756 | F-actin-capping protein subunit beta | CAPZB |
| P49411 | Elongation factor Tu, mitochondrial | TUFM |
| P54132 | Bloom syndrome protein | BLM |
| P61981 | 14-3-3 protein gamma;14-3-3 protein gamma, N-terminally processed | YWHAG |
| P62266 | 40S ribosomal protein S23 | RPS23 |
| P63010 | AP-2 complex subunit beta | AP2B1 |
| Q01831 | DNA repair protein complementing XP-C cells | XPC |
| Q06787 | Fragile X mental retardation protein 1 | FMR1 |
| Q08170 | Serine/arginine-rich splicing factor 4 | SRSF4 |
| Q12874 | Splicing factor 3A subunit 3 | SF3A3 |
| Q13472 | DNA topoisomerase 3-alpha | TOP3A |
| Q13838;O00148 | Spliceosome RNA helicase DDX39B;ATP-dependent RNA helicase DDX39A | DDX39B;DDX39A |
| Q14527 | Helicase-like transcription factor | HLTF |
| Q14692 | Ribosome biogenesis protein BMS1 homolog | BMS1 |
| Q15014 | Mortality factor 4-like protein 2 | MORF4L2 |
| Q15059 | Bromodomain-containing protein 3 | BRD3 |
| Q15293 | Reticulocalbin-1 | RCN1 |
| Q15911 | Zinc finger homeobox protein 3 | ZFHX3 |
| Q5TGY3 | AT-hook DNA-binding motif-containing protein 1 | AHDC1 |
| Q5VUA4 | Zinc finger protein 318 | ZNF318 |
| Q6P1J9 | Parafibromin | CDC73 |
| Q7Z333 | Probable helicase senataxin | SETX |
| Q7Z417 | Nuclear fragile X mental retardation-interacting protein 2 | NUFIP2 |
| Q7Z4V5 | Hepatoma-derived growth factor-related protein 2 | HDGFRP2 |
| Q8IXQ5 | Kelch-like protein 7 | KLHL7 |
| Q8IZT6 | Abnormal spindle-like microcephaly-associated protein | ASPM |
| Q8N7H5 | RNA polymerase II-associated factor 1 homolog | PAF1 |
| Q8TAQ2 | SWI/SNF complex subunit SMARCC2 | SMARCC2 |
| Q8WUA4 | General transcription factor 3C polypeptide 2 | GTF3C2 |
| Q8WXH0;Q8NF91 | Nesprin-2 | SYNE2 |
| Q92614 | Unconventional myosin-XVIIIa | MYO18A |
| Q969G3 | SWI/SNF-related matrix-associated actin-dependent regulator of chromatin subfamily E member 1 | SMARCE1 |
| Q96BK5;A0A0A6YYK5 | PIN2/TERF1-interacting telomerase inhibitor 1 | PINX1 |
| Q96GM5 | SWI/SNF-related matrix-associated actin-dependent regulator of chromatin subfamily D member 1 | SMARCD1 |
| Q96I25 | Splicing factor 45 | RBM17 |
| Q96KM6 | Zinc finger protein 512B | ZNF512B |
| Q96QC0 | Serine/threonine-protein phosphatase 1 regulatory subunit 10 | PPP1R10 |
| Q96SI9 | Spermatid perinuclear RNA-binding protein | STRBP |
| Q99575 | Ribonucleases P/MRP protein subunit POP1 | POP1 |
| Q99714 | 3-hydroxyacyl-CoA dehydrogenase type-2 | HSD17B10 |
| Q9BZJ0 | Crooked neck-like protein 1 | CRNKL1 |
| Q9H3R5 | Centromere protein H | CENPH |
| Q9H501 | ESF1 homolog | ESF1 |
| Q9H582 | Zinc finger protein 644 | ZNF644 |
| Q9H8H2 | Probable ATP-dependent RNA helicase DDX31 | DDX31 |
| Q9H9B1 | Histone-lysine N-methyltransferase EHMT1 | EHMT1 |
| Q9HCG8 | Pre-mRNA-splicing factor CWC22 homolog | CWC22 |
| Q9NR48 | Histone-lysine N-methyltransferase ASH1L | ASH1L |
| Q9NRZ9 | Lymphoid-specific helicase | HELLS |
| Q9NVU7 | Protein SDA1 homolog | SDAD1 |
| Q9P1Y6 | PHD and RING finger domain-containing protein 1 | PHRF1 |
| Q9UBB9 | Tuftelin-interacting protein 11 | TFIP11 |
| Q9ULJ3 | Zinc finger and BTB domain-containing protein 21 | ZBTB21 |
| Q9ULV4 | Coronin-1C | CORO1C |
| Q9ULX9 | Transcription factor MafF | MAFF |
| Q9Y265 | RuvB-like 1 | RUVBL1 |
| Q9Y2K7 | Lysine-specific demethylase 2A | KDM2A |
| Q9Y3X0 | Coiled-coil domain-containing protein 9 | CCDC9 |
| Q9Y4I1;Q9NQX4 | Unconventional myosin-Va | MYO5A |
| A0A0B4J1V8;Q9NQ55 | Suppressor of SWI4 1 homolog | PPAN-P2RY11;PPAN |
| O14646 | Chromodomain-helicase-DNA-binding protein 1 | CHD1 |
| O15042 | U2 snRNP-associated SURP motif-containing protein | U2SURP |
| O15226 | NF-kappa-B-repressing factor | NKRF |
| O60264;P28370 | SWI/SNF-related matrix-associated actin-dependent regulator of chromatin subfamily A member 5 | SMARCA5 |
| O60437 | Periplakin | PPL |
| O60675 | Transcription factor MafK | MAFK |
| O75494;Q8WXF0 | Serine/arginine-rich splicing factor 10 | SRSF10 |
| O75533 | Splicing factor 3B subunit 1 | SF3B1 |
| O94776 | Metastasis-associated protein MTA2 | MTA2 |
| O94875 | Sorbin and SH3 domain-containing protein 2 | SORBS2 |
| O94906 | Pre-mRNA-processing factor 6 | PRPF6 |
| O95235 | Kinesin-like protein KIF20A | KIF20A |
| O95239;Q2VIQ3 | Chromosome-associated kinesin KIF4A | KIF4A |
| O95347 | Structural maintenance of chromosomes protein 2 | SMC2 |
| O95478 | Ribosome biogenesis protein NSA2 homolog | NSA2 |
| O95696 | Bromodomain-containing protein 1 | BRD1 |
| O96019;O94805 | Actin-like protein 6A | ACTL6A |
| O96028 | Histone-lysine N-methyltransferase NSD2 | WHSC1 |
| P04792 | Heat shock protein beta-1 | HSPB1 |
| P05187;P10696 | Alkaline phosphatase, placental type;Alkaline phosphatase, placental-like | ALPP;ALPPL2 |
| P05549;Q6VUC0;Q92754;Q7Z6R9;Q92481 | Transcription factor AP-2-alpha | TFAP2A |
| P06748 | Nucleophosmin | NPM1 |
| P07910;A0A0G2JPF8 | Heterogeneous nuclear ribonucleoproteins C1/C2 | HNRNPC |
| P08651 | Nuclear factor 1 C-type | NFIC |
| P0DN76;Q01081;Q8WU68 | Splicing factor U2AF 35 kDa subunit | U2AF1 |
| P11021 | 78 kDa glucose-regulated protein | HSPA5 |
| P11142;P54652 | Heat shock cognate 71 kDa protein;Heat shock-related 70 kDa protein 2 | HSPA8;HSPA2 |
| P14866;M0QYT0;Q8WVV9 | Heterogeneous nuclear ribonucleoprotein L | HNRNPL |
| P17480 | Nucleolar transcription factor 1 | UBTF |
| P18754 | Regulator of chromosome condensation | RCC1 |
| P19338 | Nucleolin | NCL |
| P20700 | Lamin-B1 | LMNB1 |
| P31942 | Heterogeneous nuclear ribonucleoprotein H3 | HNRNPH3 |
| P31946;Q04917 | 14-3-3 protein beta/alpha;14-3-3 protein beta/alpha, N-terminally processed | YWHAB |
| P35579;P35749;REV__Q9UKV3 | Myosin-9 | MYH9 |
| P36578 | 60S ribosomal protein L4 | RPL4 |
| P43243 | Matrin-3 | MATR3 |
| P46013 | Antigen KI-67 | MKI67 |
| P46779 | 60S ribosomal protein L28 | RPL28 |
| P49756 | RNA-binding protein 25 | RBM25 |
| P49790 | Nuclear pore complex protein Nup153 | NUP153 |
| P50402 | Emerin | EMD |
| P51116 | Fragile X mental retardation syndrome-related protein 2 | FXR2 |
| P51532 | Transcription activator BRG1 | SMARCA4 |
| P51608 | Methyl-CpG-binding protein 2 | MECP2 |
| P52292 | Importin subunit alpha-1 | KPNA2 |
| P53999 | Activated RNA polymerase II transcriptional coactivator p15 | SUB1 |
| P61247 | 40S ribosomal protein S3a | RPS3A |
| P61978 | Heterogeneous nuclear ribonucleoprotein K | HNRNPK |
| P62318 | Small nuclear ribonucleoprotein Sm D3 | SNRPD3 |
| P62826 | GTP-binding nuclear protein Ran | RAN |
| P62899 | 60S ribosomal protein L31 | RPL31 |
| P62913 | 60S ribosomal protein L11 | RPL11 |
| P83916 | Chromobox protein homolog 1 | CBX1 |
| P84098 | 60S ribosomal protein L19 | RPL19 |
| Q00839 | Heterogeneous nuclear ribonucleoprotein U | HNRNPU |
| Q02241 | Kinesin-like protein KIF23 | KIF23 |
| Q03188 | Centromere protein C | CENPC |
| Q03252 | Lamin-B2 | LMNB2 |
| Q06587 | E3 ubiquitin-protein ligase RING1 | RING1 |
| Q08945 | FACT complex subunit SSRP1 | SSRP1 |
| Q12906 | Interleukin enhancer-binding factor 3 | ILF3 |
| Q13112 | Chromatin assembly factor 1 subunit B | CHAF1B |
| Q13415 | Origin recognition complex subunit 1 | ORC1 |
| Q13595 | Transformer-2 protein homolog alpha | TRA2A |
| Q13610 | Periodic tryptophan protein 1 homolog | PWP1 |
| Q13619 | Cullin-4A | CUL4A |
| Q14126 | Desmoglein-2 | DSG2 |
| Q14137 | Ribosome biogenesis protein BOP1 | BOP1 |
| Q14151 | Scaffold attachment factor B2 | SAFB2 |
| Q14498;Q86U06 | RNA-binding protein 39 | RBM39 |
| Q14683 | Structural maintenance of chromosomes protein 1A | SMC1A |
| Q15022 | Polycomb protein SUZ12 | SUZ12 |
| Q15149 | Plectin | PLEC |

| **AURKA-binding proteins in chromosomal region from mitotic cells treated with BMH-21** | | |
| --- | --- | --- |
| **Protein IDs** | **Protein names** | **Gene names** |
| P02545 | Prelamin-A/C OS=Homo sapiens GN=LMNA PE=1 SV=1 | LMNA |
| P10412 | Histone H1.4 OS=Homo sapiens GN=HIST1H1E PE=1 SV=2 | H1-4 |
| P16402 | Histone H1.3 OS=Homo sapiens GN=HIST1H1D PE=1 SV=2 | H1-3 |
| O95347 | Structural maintenance of chromosomes protein 2 OS=Homo sapiens OX=9606 GN=SMC2 PE=1 SV=2 - [SMC2_HUMAN] | SMC2 |
| P16401 | Histone H1.5 OS=Homo sapiens GN=HIST1H1B PE=1 SV=3 | H1-5 |
| P38159 | RNA-binding motif protein, X chromosome OS=Homo sapiens GN=RBMX PE=1 SV=3 | RBMX |
| Q9NR30 | Nucleolar RNA helicase 2 OS=Homo sapiens GN=DDX21 PE=1 SV=5 | DDX21 |
| P62805 | Histone H4 OS=Homo sapiens GN=HIST1H4A PE=1 SV=2 | H4C1; H4C11; H4C12 |
| P46013 | Antigen KI-67 OS=Homo sapiens GN=MKI67 PE=1 SV=2 | MKI67 |
| P84243 | Histone H3.3 OS=Homo sapiens GN=H3F3A PE=1 SV=2 | H3-3A; H3-3B |
| P46087 | Probable 28S rRNA (cytosine(4447)-C(5))-methyltransferase OS=Homo sapiens GN=NOP2 PE=1 SV=2 | NOP2 |
| Q5QNW6 | Histone H2B type 2-F OS=Homo sapiens GN=HIST2H2BF PE=1 SV=3 | H2BC18 |
| P58876 | Histone H2B type 1-D OS=Homo sapiens GN=HIST1H2BD PE=1 SV=2 | H2BC5 |
| Q99877 | Histone H2B type 1-N OS=Homo sapiens GN=HIST1H2BN PE=1 SV=3 | H2BC15 |
| P11388 | DNA topoisomerase 2-alpha OS=Homo sapiens GN=TOP2A PE=1 SV=3 | TOP2A |
| Q02539 | Histone H1.1 OS=Homo sapiens GN=HIST1H1A PE=1 SV=3 | H1-1 |
| O60814 | Histone H2B type 1-K OS=Homo sapiens GN=HIST1H2BK PE=1 SV=3 | H2BC12 |
| Q14980 | Nuclear mitotic apparatus protein 1 OS=Homo sapiens GN=NUMA1 PE=1 SV=2 | NUMA1 |
| Q9NQS7 | Inner centromere protein OS=Homo sapiens GN=INCENP PE=1 SV=3 | INCENP |
| Q9UQ35 | Serine/arginine repetitive matrix protein 2 OS=Homo sapiens GN=SRRM2 PE=1 SV=2 | SRRM2 |
| Q8N257 | Histone H2B type 3-B OS=Homo sapiens GN=HIST3H2BB PE=1 SV=3 | H2BC26 |
| O75367 | Core histone macro-H2A.1 OS=Homo sapiens GN=H2AFY PE=1 SV=4 | H2afy; H2AFY; LOC100466888 |
| P20700 | Lamin-B1 OS=Homo sapiens GN=LMNB1 PE=1 SV=2 | LMNB1 |
| Q15149 | Plectin OS=Homo sapiens GN=PLEC PE=1 SV=3 | PLEC |
| P17844 | Probable ATP-dependent RNA helicase DDX5 OS=Homo sapiens GN=DDX5 PE=1 SV=1 | DDX5 |
| Q15424 | Scaffold attachment factor B1 OS=Homo sapiens GN=SAFB PE=1 SV=4 | SAFB |
| Q9BZE4 | Nucleolar GTP-binding protein 1 OS=Homo sapiens GN=GTPBP4 PE=1 SV=3 | GTPBP4 |
| Q00839 | Heterogeneous nuclear ribonucleoprotein U OS=Homo sapiens GN=HNRNPU PE=1 SV=6 | HNRNPU |
| O00567 | Nucleolar protein 56 OS=Homo sapiens GN=NOP56 PE=1 SV=4 | NOP56 |
| P22087 | rRNA 2'-O-methyltransferase fibrillarin OS=Homo sapiens GN=FBL PE=1 SV=2 | FBL |
| Q03252 | Lamin-B2 OS=Homo sapiens GN=LMNB2 PE=1 SV=4 | LMNB2 |
| O60264 | SWI/SNF-related matrix-associated actin-dependent regulator of chromatin subfamily A member 5 OS=Homo sapiens GN=SMARCA5 PE=1 SV=1 | SMARCA5 |
| Q9UIG0 | Tyrosine-protein kinase BAZ1B OS=Homo sapiens GN=BAZ1B PE=1 SV=2 | BAZ1B |
| O60832 | H/ACA ribonucleoprotein complex subunit 4 OS=Homo sapiens GN=DKC1 PE=1 SV=3 | DKC1 |
| Q96PK6 | RNA-binding protein 14 OS=Homo sapiens GN=RBM14 PE=1 SV=2 | RBM14 |
| Q9H307 | Pinin OS=Homo sapiens GN=PNN PE=1 SV=4 |  |
| Q16777 | Histone H2A type 2-C OS=Homo sapiens GN=HIST2H2AC PE=1 SV=4 | H2AC20 |
| Q9H6F5 | Coiled-coil domain-containing protein 86 OS=Homo sapiens GN=CCDC86 PE=1 SV=1 | CCDC86 |
| P62987 | Ubiquitin-60S ribosomal protein L40 OS=Homo sapiens GN=UBA52 PE=1 SV=2 | UBA52 |
| P62424 | 60S ribosomal protein L7a OS=Homo sapiens GN=RPL7A PE=1 SV=2 | RPL7A |
| Q92841 | Probable ATP-dependent RNA helicase DDX17 OS=Homo sapiens GN=DDX17 PE=1 SV=2 | DDX17 |
| Q8WYP5 | Protein ELYS OS=Homo sapiens GN=AHCTF1 PE=1 SV=3 | AHCTF1 |
| O76021 | Ribosomal L1 domain-containing protein 1 OS=Homo sapiens GN=RSL1D1 PE=1 SV=3 | RSL1D1 |
| Q9NVP1 | ATP-dependent RNA helicase DDX18 OS=Homo sapiens GN=DDX18 PE=1 SV=2 | DDX18 |
| Q8WWQ0 | PH-interacting protein OS=Homo sapiens GN=PHIP PE=1 SV=2 | PHIP |
| Q9BQ39 | ATP-dependent RNA helicase DDX50 OS=Homo sapiens GN=DDX50 PE=1 SV=1 | DDX50 |
| Q7L7L0 | Histone H2A type 3 OS=Homo sapiens GN=HIST3H2A PE=1 SV=3 | H2AC25 |
| P11387 | DNA topoisomerase 1 OS=Homo sapiens GN=TOP1 PE=1 SV=2 | TOP1 |
| Q96GQ7 | Probable ATP-dependent RNA helicase DDX27 OS=Homo sapiens GN=DDX27 PE=1 SV=2 | DDX27 |
| Q08945 | FACT complex subunit SSRP1 OS=Homo sapiens GN=SSRP1 PE=1 SV=1 | SSRP1 |
| Q9Y5B9 | FACT complex subunit SPT16 OS=Homo sapiens GN=SUPT16H PE=1 SV=1 | SUPT16H |
| Q9ULW0 | Targeting protein for Xklp2 OS=Homo sapiens GN=TPX2 PE=1 SV=2 | TPX2 |
| P36578 | 60S ribosomal protein L4 OS=Homo sapiens GN=RPL4 PE=1 SV=5 | RPL4 |
| Q9UKV3 | Apoptotic chromatin condensation inducer in the nucleus OS=Homo sapiens GN=ACIN1 PE=1 SV=2 | ACIN1 |
| Q15029 | 116 kDa U5 small nuclear ribonucleoprotein component OS=Homo sapiens GN=EFTUD2 PE=1 SV=1 | EFTUD2 |
| Q9Y2W1 | Thyroid hormone receptor-associated protein 3 OS=Homo sapiens GN=THRAP3 PE=1 SV=2 | THRAP3 |
| Q6P2Q9 | Pre-mRNA-processing-splicing factor 8 OS=Homo sapiens GN=PRPF8 PE=1 SV=2 | PRPF8 |
| P22626 | Heterogeneous nuclear ribonucleoproteins A2/B1 OS=Homo sapiens GN=HNRNPA2B1 PE=1 SV=2 | HNRNPA2B1 |
| O00571 | ATP-dependent RNA helicase DDX3X OS=Homo sapiens GN=DDX3X PE=1 SV=3 | DDX3X |
| Q14137 | Ribosome biogenesis protein BOP1 OS=Homo sapiens GN=BOP1 PE=1 SV=2 | BOP1 |
| Q9UDX5 | Mitochondrial fission process protein 1 OS=Homo sapiens GN=MTFP1 PE=1 SV=1 | MTFP1 |
| Q5SSJ5 | Heterochromatin protein 1-binding protein 3 OS=Homo sapiens GN=HP1BP3 PE=1 SV=1 | HP1BP3 |
| P52272 | Heterogeneous nuclear ribonucleoprotein M OS=Homo sapiens GN=HNRNPM PE=1 SV=3 | HNRNPM |
| Q08211 | ATP-dependent RNA helicase A OS=Homo sapiens GN=DHX9 PE=1 SV=4 | DHX9 |
| Q7Z7K6 | Centromere protein V OS=Homo sapiens GN=CENPV PE=1 SV=1 | CENPV |
| P68104 | Elongation factor 1-alpha 1 OS=Homo sapiens GN=EEF1A1 PE=1 SV=1 | EEF1A1 |
| Q5QJE6 | Deoxynucleotidyltransferase terminal-interacting protein 2 OS=Homo sapiens GN=DNTTIP2 PE=1 SV=2 | DNTTIP2 |
| P09874 | Poly [ADP-ribose] polymerase 1 OS=Homo sapiens GN=PARP1 PE=1 SV=4 | PARP1 |
| Q16629 | Serine/arginine-rich splicing factor 7 OS=Homo sapiens GN=SRSF7 PE=1 SV=1 | SRSF7 |
| Q8WTT2 | Nucleolar complex protein 3 homolog OS=Homo sapiens GN=NOC3L PE=1 SV=1 | NOC3L |
| Q14151 | Scaffold attachment factor B2 OS=Homo sapiens GN=SAFB2 PE=1 SV=1 | SAFB2 |
| P09651 | Heterogeneous nuclear ribonucleoprotein A1 OS=Homo sapiens GN=HNRNPA1 PE=1 SV=5 | HNRNPA1 |
| P16104 | Histone H2AX OS=Homo sapiens GN=H2AFX PE=1 SV=2 | H2AX |
| P35579 | Myosin-9 OS=Homo sapiens GN=MYH9 PE=1 SV=4 | MYH9 |
| Q02878 | 60S ribosomal protein L6 OS=Homo sapiens GN=RPL6 PE=1 SV=3 | RPL6 |
| Q13247 | Serine/arginine-rich splicing factor 6 OS=Homo sapiens GN=SRSF6 PE=1 SV=2 | SRSF6 |
| P42167 | Lamina-associated polypeptide 2, isoforms beta/gamma OS=Homo sapiens GN=TMPO PE=1 SV=2 | TMPO |
| O75643 | U5 small nuclear ribonucleoprotein 200 kDa helicase OS=Homo sapiens GN=SNRNP200 PE=1 SV=2 | SNRNP200 |
| P50402 | Emerin OS=Homo sapiens GN=EMD PE=1 SV=1 | EMD |
| P62906 | 60S ribosomal protein L10a OS=Homo sapiens GN=RPL10A PE=1 SV=2 | RPL10A |
| Q5JTH9 | RRP12-like protein OS=Homo sapiens GN=RRP12 PE=1 SV=2 | RRP12 |
| Q9NY93 | Probable ATP-dependent RNA helicase DDX56 OS=Homo sapiens GN=DDX56 PE=1 SV=1 | DDX56 |
| P26373 | 60S ribosomal protein L13 OS=Homo sapiens GN=RPL13 PE=1 SV=4 | RPL13 |
| Q9BVP2 | Guanine nucleotide-binding protein-like 3 OS=Homo sapiens GN=GNL3 PE=1 SV=2 | GNL3 |
| Q5BKZ1 | DBIRD complex subunit ZNF326 OS=Homo sapiens GN=ZNF326 PE=1 SV=2 | ZNF326 |
| Q02880 | DNA topoisomerase 2-beta OS=Homo sapiens GN=TOP2B PE=1 SV=3 | TOP2B |
| Q53HL2 | Borealin OS=Homo sapiens GN=CDCA8 PE=1 SV=2 | CDCA8 |
| P56182 | Ribosomal RNA processing protein 1 homolog A OS=Homo sapiens GN=RRP1 PE=1 SV=1 | RRP1 |
| Q9NYF8 | Bcl-2-associated transcription factor 1 OS=Homo sapiens GN=BCLAF1 PE=1 SV=2 | BCLAF1 |
| Q14839 | Chromodomain-helicase-DNA-binding protein 4 OS=Homo sapiens GN=CHD4 PE=1 SV=2 | CHD4 |
| P06748 | Nucleophosmin OS=Homo sapiens GN=NPM1 PE=1 SV=2 | NPM1 |
| Q9NWH9 | SAFB-like transcription modulator OS=Homo sapiens GN=SLTM PE=1 SV=2 | SLTM |
| Q9H0A0 | RNA cytidine acetyltransferase OS=Homo sapiens GN=NAT10 PE=1 SV=2 | NAT10 |
| P07305 | Histone H1.0 OS=Homo sapiens GN=H1F0 PE=1 SV=3 | H1-0 |
| P35659 | Protein DEK OS=Homo sapiens GN=DEK PE=1 SV=1 | DEK |
| Q8IVT2 | Mitotic interactor and substrate of PLK1 OS=Homo sapiens GN=MISP PE=1 SV=1 | MISP |
| Q96GD4 | Aurora kinase B OS=Homo sapiens GN=AURKB PE=1 SV=3 | AURKB |
| P43243 | Matrin-3 OS=Homo sapiens GN=MATR3 PE=1 SV=2 | MATR3 |
| O94906 | Pre-mRNA-processing factor 6 OS=Homo sapiens GN=PRPF6 PE=1 SV=1 | PRPF6 |
| Q15050 | Ribosome biogenesis regulatory protein homolog OS=Homo sapiens GN=RRS1 PE=1 SV=2 | RRS1 |
| Q13435 | Splicing factor 3B subunit 2 OS=Homo sapiens GN=SF3B2 PE=1 SV=2 | SF3B2 |
| Q14807 | Kinesin-like protein KIF22 OS=Homo sapiens GN=KIF22 PE=1 SV=5 | KIF22 |
| Q8TDD1 | ATP-dependent RNA helicase DDX54 OS=Homo sapiens GN=DDX54 PE=1 SV=2 | DDX54 |
| P62917 | 60S ribosomal protein L8 OS=Homo sapiens GN=RPL8 PE=1 SV=2 | RPL8 |
| P14618 | Pyruvate kinase PKM OS=Homo sapiens GN=PKM PE=1 SV=4 | PKM |
| P18124 | 60S ribosomal protein L7 OS=Homo sapiens GN=RPL7 PE=1 SV=1 | RPL7 |
| P38919 | Eukaryotic initiation factor 4A-III OS=Homo sapiens GN=EIF4A3 PE=1 SV=4 | EIF4A3 |
| Q9H0H5 | Rac GTPase-activating protein 1 OS=Homo sapiens GN=RACGAP1 PE=1 SV=1 | RACGAP1 |
| Q13523 | Serine/threonine-protein kinase PRP4 homolog OS=Homo sapiens GN=PRPF4B PE=1 SV=3 | PRPF4B |
| P18583 | Protein SON OS=Homo sapiens GN=SON PE=1 SV=4 | SON |
| Q9UMS4 | Pre-mRNA-processing factor 19 OS=Homo sapiens GN=PRPF19 PE=1 SV=1 | PRPF19 |
| Q9BUQ8 | Probable ATP-dependent RNA helicase DDX23 OS=Homo sapiens GN=DDX23 PE=1 SV=3 | DDX23 |
| P12956 | X-ray repair cross-complementing protein 6 OS=Homo sapiens GN=XRCC6 PE=1 SV=2 | XRCC6 |
| P38646 | Stress-70 protein, mitochondrial OS=Homo sapiens GN=HSPA9 PE=1 SV=2 | HSPA9 |
| Q14978 | Nucleolar and coiled-body phosphoprotein 1 OS=Homo sapiens GN=NOLC1 PE=1 SV=2 | NOLC1 |
| Q9NSI6 | Bromodomain and WD repeat-containing protein 1 OS=Homo sapiens GN=BRWD1 PE=1 SV=4 | BRWD1 |
| Q9H4L4 | Sentrin-specific protease 3 OS=Homo sapiens GN=SENP3 PE=1 SV=2 | SENP3 |
| Q99459 | Cell division cycle 5-like protein OS=Homo sapiens GN=CDC5L PE=1 SV=2 | CDC5L |
| Q07666 | KH domain-containing, RNA-binding, signal transduction-associated protein 1 OS=Homo sapiens GN=KHDRBS1 PE=1 SV=1 | KHDRBS1 |
| Q9NXF1 | Testis-expressed sequence 10 protein OS=Homo sapiens GN=TEX10 PE=1 SV=2 | TEX10 |
| P19338 | Nucleolin OS=Homo sapiens GN=NCL PE=1 SV=3 | NCL |
| Q14676 | Mediator of DNA damage checkpoint protein 1 OS=Homo sapiens GN=MDC1 PE=1 SV=3 | MDC1 |
| Q9Y2X3 | Nucleolar protein 58 OS=Homo sapiens GN=NOP58 PE=1 SV=1 | NOP58 |
| P07199 | Major centromere autoantigen B OS=Homo sapiens GN=CENPB PE=1 SV=2 | CENPB |
| P06576 | ATP synthase subunit beta, mitochondrial OS=Homo sapiens GN=ATP5B PE=1 SV=3 | ATP5F1B |
| Q9H6R4 | Nucleolar protein 6 OS=Homo sapiens GN=NOL6 PE=1 SV=2 | NOL6 |
| Q8WWI1 | LIM domain only protein 7 OS=Homo sapiens GN=LMO7 PE=1 SV=3 | LMO7 |
| Q99848 | Probable rRNA-processing protein EBP2 OS=Homo sapiens GN=EBNA1BP2 PE=1 SV=2 | EBNA1BP2 |
| Q07020 | 60S ribosomal protein L18 OS=Homo sapiens GN=RPL18 PE=1 SV=2 | RPL18 |
| P51991 | Heterogeneous nuclear ribonucleoprotein A3 OS=Homo sapiens GN=HNRNPA3 PE=1 SV=2 | HNRNPA3 |
| Q9P275 | Ubiquitin carboxyl-terminal hydrolase 36 OS=Homo sapiens GN=USP36 PE=1 SV=3 | |
| P02768 | Serum albumin OS=Homo sapiens GN=ALB PE=1 SV=2 | ALB |
| O00159 | Unconventional myosin-Ic OS=Homo sapiens GN=MYO1C PE=1 SV=4 | MYO1C |
| P17480 | Nucleolar transcription factor 1 OS=Homo sapiens GN=UBTF PE=1 SV=1 | UBTF |
| Q14683 | Structural maintenance of chromosomes protein 1A OS=Homo sapiens GN=SMC1A PE=1 SV=2 | SMC1A |
| Q14974 | Importin subunit beta-1 OS=Homo sapiens GN=KPNB1 PE=1 SV=2 | KPNB1 |
| Q9BTC0 | Death-inducer obliterator 1 OS=Homo sapiens GN=DIDO1 PE=1 SV=5 | DIDO1 |
| A8CG34 | Nuclear envelope pore membrane protein POM 121C OS=Homo sapiens GN=POM121C PE=1 SV=2 |  |
| Q9BXY0 | Protein MAK16 homolog OS=Homo sapiens GN=MAK16 PE=1 SV=2 | MAK16 |
| Q9P1Y6 | PHD and RING finger domain-containing protein 1 OS=Homo sapiens GN=PHRF1 PE=1 SV=3 | PHRF1 |
| P49454 | Centromere protein F OS=Homo sapiens GN=CENPF PE=1 SV=2 |  |
| Q9BQG0 | Myb-binding protein 1A OS=Homo sapiens GN=MYBBP1A PE=1 SV=2 | MYBBP1A |
| Q9Y2K7 | Lysine-specific demethylase 2A OS=Homo sapiens GN=KDM2A PE=1 SV=3 | KDM2A |
| O43159 | Ribosomal RNA-processing protein 8 OS=Homo sapiens GN=RRP8 PE=1 SV=2 | RRP8 |
| P62995 | Transformer-2 protein homolog beta OS=Homo sapiens GN=TRA2B PE=1 SV=1 | TRA2B |

| **AURKA-binding proteins in chromosomal region from mitotic cells treated with CX5461** | | |
| --- | --- | --- |
| **Protein IDs** | **Protein names** | **Gene names** |
| P19105;O14950;P24844 | Myosin regulatory light chain 12A;Myosin regulatory light chain 12B | MYL12A;MYL12B |
| O43175 | D-3-phosphoglycerate dehydrogenase | PHGDH |
| O43823 | A-kinase anchor protein 8 | AKAP8 |
| O60287 | Nucleolar pre-ribosomal-associated protein 1 | URB1 |
| O60318 | Germinal-center associated nuclear protein | MCM3AP |
| O60504 | Vinexin | SORBS3 |
| O95391 | Pre-mRNA-splicing factor SLU7 | SLU7 |
| O95425 | Supervillin | SVIL |
| O95602 | DNA-directed RNA polymerase I subunit RPA1 | POLR1A |
| P22314 | Ubiquitin-like modifier-activating enzyme 1 | UBA1 |
| P29375 | Lysine-specific demethylase 5A | KDM5A |
| P47756 | F-actin-capping protein subunit beta | CAPZB |
| P49411 | Elongation factor Tu, mitochondrial | TUFM |
| P54132 | Bloom syndrome protein | BLM |
| P61981 | 14-3-3 protein gamma;14-3-3 protein gamma, N-terminally processed | YWHAG |
| P62266 | 40S ribosomal protein S23 | RPS23 |
| P63010 | AP-2 complex subunit beta | AP2B1 |
| Q01831 | DNA repair protein complementing XP-C cells | XPC |
| Q06787 | Fragile X mental retardation protein 1 | FMR1 |
| Q08170 | Serine/arginine-rich splicing factor 4 | SRSF4 |
| Q12874 | Splicing factor 3A subunit 3 | SF3A3 |
| Q13472 | DNA topoisomerase 3-alpha | TOP3A |
| Q13838;O00148 | Spliceosome RNA helicase DDX39B;ATP-dependent RNA helicase DDX39A | DDX39B;DDX39A |
| Q14527 | Helicase-like transcription factor | HLTF |
| Q14692 | Ribosome biogenesis protein BMS1 homolog | BMS1 |
| Q15014 | Mortality factor 4-like protein 2 | MORF4L2 |
| Q15059 | Bromodomain-containing protein 3 | BRD3 |
| Q15293 | Reticulocalbin-1 | RCN1 |
| Q15911 | Zinc finger homeobox protein 3 | ZFHX3 |
| Q5TGY3 | AT-hook DNA-binding motif-containing protein 1 | AHDC1 |
| Q5VUA4 | Zinc finger protein 318 | ZNF318 |
| Q6P1J9 | Parafibromin | CDC73 |
| Q7Z333 | Probable helicase senataxin | SETX |
| Q7Z417 | Nuclear fragile X mental retardation-interacting protein 2 | NUFIP2 |
| Q7Z4V5 | Hepatoma-derived growth factor-related protein 2 | HDGFRP2 |
| Q8IXQ5 | Kelch-like protein 7 | KLHL7 |
| Q8IZT6 | Abnormal spindle-like microcephaly-associated protein | ASPM |
| Q8N7H5 | RNA polymerase II-associated factor 1 homolog | PAF1 |
| Q8TAQ2 | SWI/SNF complex subunit SMARCC2 | SMARCC2 |
| Q8WUA4 | General transcription factor 3C polypeptide 2 | GTF3C2 |
| Q8WXH0;Q8NF91 | Nesprin-2 | SYNE2 |
| Q92614 | Unconventional myosin-XVIIIa | MYO18A |
| Q969G3 | SWI/SNF-related matrix-associated actin-dependent regulator of chromatin subfamily E member 1 | SMARCE1 |
| Q96BK5;A0A0A6YYK5 | PIN2/TERF1-interacting telomerase inhibitor 1 | PINX1 |
| Q96GM5 | SWI/SNF-related matrix-associated actin-dependent regulator of chromatin subfamily D member 1 | SMARCD1 |
| Q96I25 | Splicing factor 45 | RBM17 |
| Q96KM6 | Zinc finger protein 512B | ZNF512B |
| Q96QC0 | Serine/threonine-protein phosphatase 1 regulatory subunit 10 | PPP1R10 |
| Q96SI9 | Spermatid perinuclear RNA-binding protein | STRBP |
| Q99575 | Ribonucleases P/MRP protein subunit POP1 | POP1 |
| Q99714 | 3-hydroxyacyl-CoA dehydrogenase type-2 | HSD17B10 |
| Q9BZJ0 | Crooked neck-like protein 1 | CRNKL1 |
| Q9H3R5 | Centromere protein H | CENPH |
| Q9H501 | ESF1 homolog | ESF1 |
| Q9H582 | Zinc finger protein 644 | ZNF644 |
| Q9H8H2 | Probable ATP-dependent RNA helicase DDX31 | DDX31 |
| Q9H9B1 | Histone-lysine N-methyltransferase EHMT1 | EHMT1 |
| Q9HCG8 | Pre-mRNA-splicing factor CWC22 homolog | CWC22 |
| Q9NR48 | Histone-lysine N-methyltransferase ASH1L | ASH1L |
| Q9NRZ9 | Lymphoid-specific helicase | HELLS |
| Q9NVU7 | Protein SDA1 homolog | SDAD1 |
| Q9P1Y6 | PHD and RING finger domain-containing protein 1 | PHRF1 |
| Q9UBB9 | Tuftelin-interacting protein 11 | TFIP11 |
| Q9ULJ3 | Zinc finger and BTB domain-containing protein 21 | ZBTB21 |
| Q9ULV4 | Coronin-1C | CORO1C |
| Q9ULX9 | Transcription factor MafF | MAFF |
| Q9Y265 | RuvB-like 1 | RUVBL1 |
| Q9Y2K7 | Lysine-specific demethylase 2A | KDM2A |
| Q9Y3X0 | Coiled-coil domain-containing protein 9 | CCDC9 |
| Q9Y4I1;Q9NQX4 | Unconventional myosin-Va | MYO5A |
| A0A0A6YYL6;P18621 | 60S ribosomal protein L17 | RPL17-C18orf32;RPL17 |
| A0A0B4J1V8;Q9NQ55 | Suppressor of SWI4 1 homolog | PPAN-P2RY11;PPAN |
| A6NHR9 | Structural maintenance of chromosomes flexible hinge domain-containing protein 1 | SMCHD1 |
| A8CG34;Q6PJE2;A6NF01 | Nuclear envelope pore membrane protein POM 121C | POM121C |
| O14646 | Chromodomain-helicase-DNA-binding protein 1 | CHD1 |
| O14647;A0A0D9SFI3 | Chromodomain-helicase-DNA-binding protein 2 | CHD2 |
| O15042 | U2 snRNP-associated SURP motif-containing protein | U2SURP |
| O15226 | NF-kappa-B-repressing factor | NKRF |
| O43143 | Pre-mRNA-splicing factor ATP-dependent RNA helicase DHX15 | DHX15 |
| O43172 | U4/U6 small nuclear ribonucleoprotein Prp4 | PRPF4 |
| O43248 | Homeobox protein Hox-C11 | HOXC11 |
| O43290 | U4/U6.U5 tri-snRNP-associated protein 1 | SART1 |
| O75152;A0A1B0GUI2 | Zinc finger CCCH domain-containing protein 11A | ZC3H11A |
| O75494;Q8WXF0 | Serine/arginine-rich splicing factor 10 | SRSF10 |
| O75530 | Polycomb protein EED | EED |
| O75531 | Barrier-to-autointegration factor;Barrier-to-autointegration factor, N-terminally processed | BANF1 |
| O76021 | Ribosomal L1 domain-containing protein 1 | RSL1D1 |
| O94875 | Sorbin and SH3 domain-containing protein 2 | SORBS2 |
| O94880 | PHD finger protein 14 | PHF14 |
| O95347 | Structural maintenance of chromosomes protein 2 | SMC2 |
| O95478 | Ribosome biogenesis protein NSA2 homolog | NSA2 |
| O95696 | Bromodomain-containing protein 1 | BRD1 |
| O96028 | Histone-lysine N-methyltransferase NSD2 | WHSC1 |
| P05187;P10696 | Alkaline phosphatase, placental type;Alkaline phosphatase, placental-like | ALPP;ALPPL2 |
| P07910;A0A0G2JPF8 | Heterogeneous nuclear ribonucleoproteins C1/C2 | HNRNPC |
| P0DN76;Q01081 | Splicing factor U2AF 35 kDa subunit | U2AF1 |
| P11940;Q4VXU2 | Polyadenylate-binding protein 1 | PABPC1 |
| P14866;M0QYT0 | Heterogeneous nuclear ribonucleoprotein L | HNRNPL |
| P17480 | Nucleolar transcription factor 1 | UBTF |
| P18124 | 60S ribosomal protein L7 | RPL7 |
| P18754 | Regulator of chromosome condensation | RCC1 |
| P19338 | Nucleolin | NCL |
| P20700 | Lamin-B1 | LMNB1 |
| P22087 | rRNA 2-O-methyltransferase fibrillarin | FBL |
| P23528 | Cofilin-1 | CFL1 |
| P30876 | DNA-directed RNA polymerase II subunit RPB2 | POLR2B |
| P31942 | Heterogeneous nuclear ribonucleoprotein H3 | HNRNPH3 |
| P31946;Q04917 | 14-3-3 protein beta/alpha;14-3-3 protein beta/alpha, N-terminally processed | YWHAB |
| P35579;P35749 | Myosin-9 | MYH9 |
| P35658 | Nuclear pore complex protein Nup214 | NUP214 |
| P36578 | 60S ribosomal protein L4 | RPL4 |
| P38919 | Eukaryotic initiation factor 4A-III;Eukaryotic initiation factor 4A-III, N-terminally processed | EIF4A3 |
| P42696 | RNA-binding protein 34 | RBM34 |
| P43243 | Matrin-3 | MATR3 |
| P46013 | Antigen KI-67 | MKI67 |
| P46087 | Probable 28S rRNA (cytosine(4447)-C(5))-methyltransferase | NOP2 |
| P47914 | 60S ribosomal protein L29 | RPL29 |
| P49756 | RNA-binding protein 25 | RBM25 |
| P49790 | Nuclear pore complex protein Nup153 | NUP153 |
| P50914 | 60S ribosomal protein L14 | RPL14 |
| P51114 | Fragile X mental retardation syndrome-related protein 1 | FXR1 |
| P51116 | Fragile X mental retardation syndrome-related protein 2 | FXR2 |
| P51531 | Probable global transcription activator SNF2L2 | SMARCA2 |
| P51532 | Transcription activator BRG1 | SMARCA4 |
| P51608 | Methyl-CpG-binding protein 2 | MECP2 |
| P52298 | Nuclear cap-binding protein subunit 2 | NCBP2 |
| P53999 | Activated RNA polymerase II transcriptional coactivator p15 | SUB1 |
| P62081 | 40S ribosomal protein S7 | RPS7 |
| P62263 | 40S ribosomal protein S14 | RPS14 |
| P62826 | GTP-binding nuclear protein Ran | RAN |
| P62829 | 60S ribosomal protein L23 | RPL23 |
| P62899 | 60S ribosomal protein L31 | RPL31 |
| P62917 | 60S ribosomal protein L8 | RPL8 |
| P78316 | Nucleolar protein 14 | NOP14 |
| Q00839 | Heterogeneous nuclear ribonucleoprotein U | HNRNPU |
| Q02241 | Kinesin-like protein KIF23 | KIF23 |
| Q03188 | Centromere protein C | CENPC |
| Q03252 | Lamin-B2 | LMNB2 |
| Q03701 | CCAAT/enhancer-binding protein zeta | CEBPZ |
| Q08945 | FACT complex subunit SSRP1 | SSRP1 |
| Q12965;O00160 | Unconventional myosin-Ie | MYO1E |
| Q13112 | Chromatin assembly factor 1 subunit B | CHAF1B |
| Q13185 | Chromobox protein homolog 3 | CBX3 |
| Q13523 | Serine/threonine-protein kinase PRP4 homolog | PRPF4B |
| Q13595 | Transformer-2 protein homolog alpha | TRA2A |
| Q14103 | Heterogeneous nuclear ribonucleoprotein D0 | HNRNPD |
| Q14137 | Ribosome biogenesis protein BOP1 | BOP1 |
| Q14669 | E3 ubiquitin-protein ligase TRIP12 | TRIP12 |
| Q14683 | Structural maintenance of chromosomes protein 1A | SMC1A |
| Q14839;Q8TDI0 | Chromodomain-helicase-DNA-binding protein 4 | CHD4 |
| Q14966 | Zinc finger protein 638 | ZNF638 |
| Q15022 | Polycomb protein SUZ12 | SUZ12 |
| Q15428 | Splicing factor 3A subunit 2 | SF3A2 |
| Q15717;Q12926;P26378 | ELAV-like protein 1 | ELAVL1 |
| Q16352 | Alpha-internexin | INA |
| Q16531 | DNA damage-binding protein 1 | DDB1 |
| Q16666 | Gamma-interferon-inducible protein 16 | IFI16 |
| Q16778;P33778;P23527 | Histone H2B type 2-E;Histone H2B type 1-B;Histone H2B type 1-O | HIST2H2BE;HIST1H2BB |
| Q562F6 | Shugoshin-like 2 | SGOL2 |
| Q5QJE6 | Deoxynucleotidyltransferase terminal-interacting protein 2 | DNTTIP2 |
| Q5TEC6 | Histone H3 | HIST2H3PS2 |
| Q5UIP0 | Telomere-associated protein RIF1 | RIF1 |
| Q6KC79 | Nipped-B-like protein | NIPBL |
| Q6P0N0 | Mis18-binding protein 1 | MIS18BP1 |
| Q6SJ93 | Protein FAM111B | FAM111B |
| Q6UN15;A0A0B4J203 | Pre-mRNA 3-end-processing factor FIP1 | FIP1L1 |
| Q76FK4;Q8NAA4 | Nucleolar protein 8 | NOL8 |
| Q7L2E3 | Putative ATP-dependent RNA helicase DHX30 | DHX30 |
| Q7Z6E9 | E3 ubiquitin-protein ligase RBBP6 | RBBP6 |
| Q86SQ0 | Pleckstrin homology-like domain family B member 2 | PHLDB2 |
| Q86V81 | THO complex subunit 4 | ALYREF |
| Q8IVT2 | Mitotic interactor and substrate of PLK1 | MISP |
| Q8IY18 | Structural maintenance of chromosomes protein 5 | SMC5 |
| Q8N3X1 | Formin-binding protein 4 | FNBP4 |
| Q8NAP3 | Zinc finger and BTB domain-containing protein 38 | ZBTB38 |
| Q8NC56 | LEM domain-containing protein 2 | LEMD2 |
| Q8NEJ9 | Neuroguidin | NGDN |
| Q8NEY8 | Periphilin-1 | PPHLN1 |
| Q8WTT2 | Nucleolar complex protein 3 homolog | NOC3L |
| Q8WWI1 | LIM domain only protein 7 | LMO7 |
| Q8WWK9 | Cytoskeleton-associated protein 2 | CKAP2 |
| Q8WWQ0 | PH-interacting protein | PHIP |
| Q92576 | PHD finger protein 3 | PHF3 |
| Q92804 | TATA-binding protein-associated factor 2N | TAF15 |
| Q96G21 | U3 small nucleolar ribonucleoprotein protein IMP4 | IMP4 |
| Q96T23 | Remodeling and spacing factor 1 | RSF1 |
| Q96T58 | Msx2-interacting protein | SPEN |
| Q99549 | M-phase phosphoprotein 8 | MPHOSPH8 |
| Q99590 | Protein SCAF11 | SCAF11 |
| Q9BQG0 | Myb-binding protein 1A | MYBBP1A |
| Q9BSC4 | Nucleolar protein 10 | NOL10 |
| Q9BTC0 | Death-inducer obliterator 1 | DIDO1 |
| Q9C0J8 | pre-mRNA 3 end processing protein WDR33 | WDR33 |
| Q9H2Y7 | Zinc finger protein 106 | ZNF106 |
| Q9H307 | Pinin | PNN |
| Q9H4F8 | SPARC-related modular calcium-binding protein 1 | SMOC1 |
| Q9H6F5 | Coiled-coil domain-containing protein 86 | CCDC86 |
| Q9H6R4 | Nucleolar protein 6 | NOL6 |
| Q9HC52;O95503 | Chromobox protein homolog 8 | CBX8 |
| Q9HCD5 | Nuclear receptor coactivator 5 | NCOA5 |
| Q9NQZ2 | Something about silencing protein 10 | UTP3 |
| Q9NR30 | Nucleolar RNA helicase 2 | DDX21 |
| Q9NVI7;Q5T9A4 | ATPase family AAA domain-containing protein 3A;ATPase family AAA domain-containing protein 3B | ATAD3A;ATAD3B |
| Q9NWH9 | SAFB-like transcription modulator | SLTM |
| Q9NYF8 | Bcl-2-associated transcription factor 1 | BCLAF1 |
| Q9NYH9 | U3 small nucleolar RNA-associated protein 6 homolog | UTP6 |
| Q9NZM5 | Glioma tumor suppressor candidate region gene 2 protein | GLTSCR2 |
| Q9P0U3 | Sentrin-specific protease 1 | SENP1 |
| Q9P275 | Ubiquitin carboxyl-terminal hydrolase 36 | USP36 |
| Q9UH99 | SUN domain-containing protein 2 | SUN2 |
| Q9UHB6 | LIM domain and actin-binding protein 1 | LIMA1 |
| Q9UKV3 | Apoptotic chromatin condensation inducer in the nucleus | ACIN1 |
| Q9ULI0 | ATPase family AAA domain-containing protein 2B | ATAD2B |
| Q9UMN6 | Histone-lysine N-methyltransferase 2B | KMT2B |
| Q9UQE7 | Structural maintenance of chromosomes protein 3 | SMC3 |
| Q9Y2W1 | Thyroid hormone receptor-associated protein 3 | THRAP3 |
| Q9Y2X3 | Nucleolar protein 58 | NOP58 |
| Q9Y2X9 | Zinc finger protein 281 | ZNF281 |
| Q9Y3C6 | Peptidyl-prolyl cis-trans isomerase-like 1 | PPIL1 |
| Q9Y5J1 | U3 small nucleolar RNA-associated protein 18 homolog | UTP18 |
