# Supplementary material for: Pre-rRNAs control mitosis by maintaining chromosomal segregation through protecting SMC2 from AURKA-mediated phosphorylation
Source: Cell Death Dis. 2025 Nov 7;16(1):812. doi: 10.1038/s41419-025-08169-9 (PMC12594857; doi:10.1038/s41419-025-08169-9)

Figure 1G:


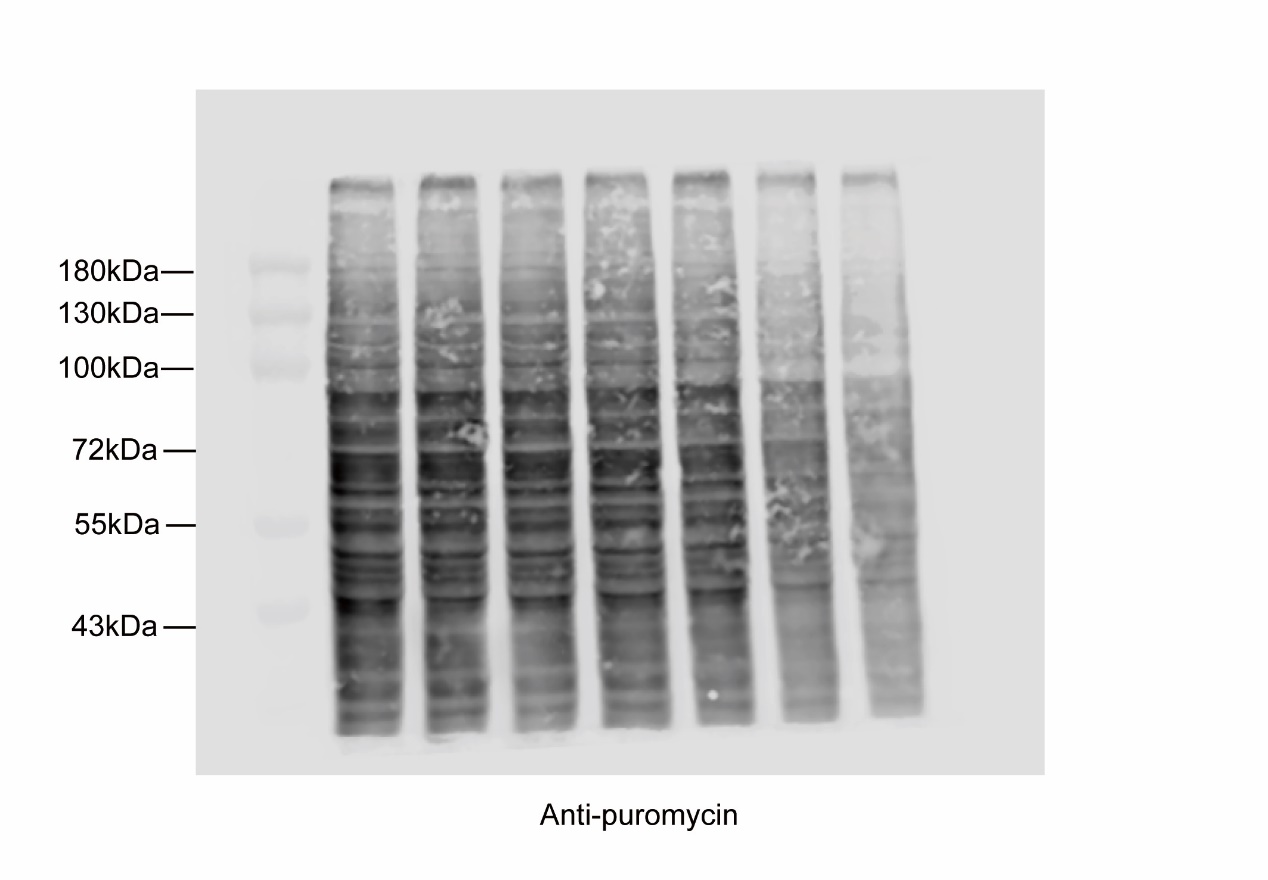


Figure 2E:


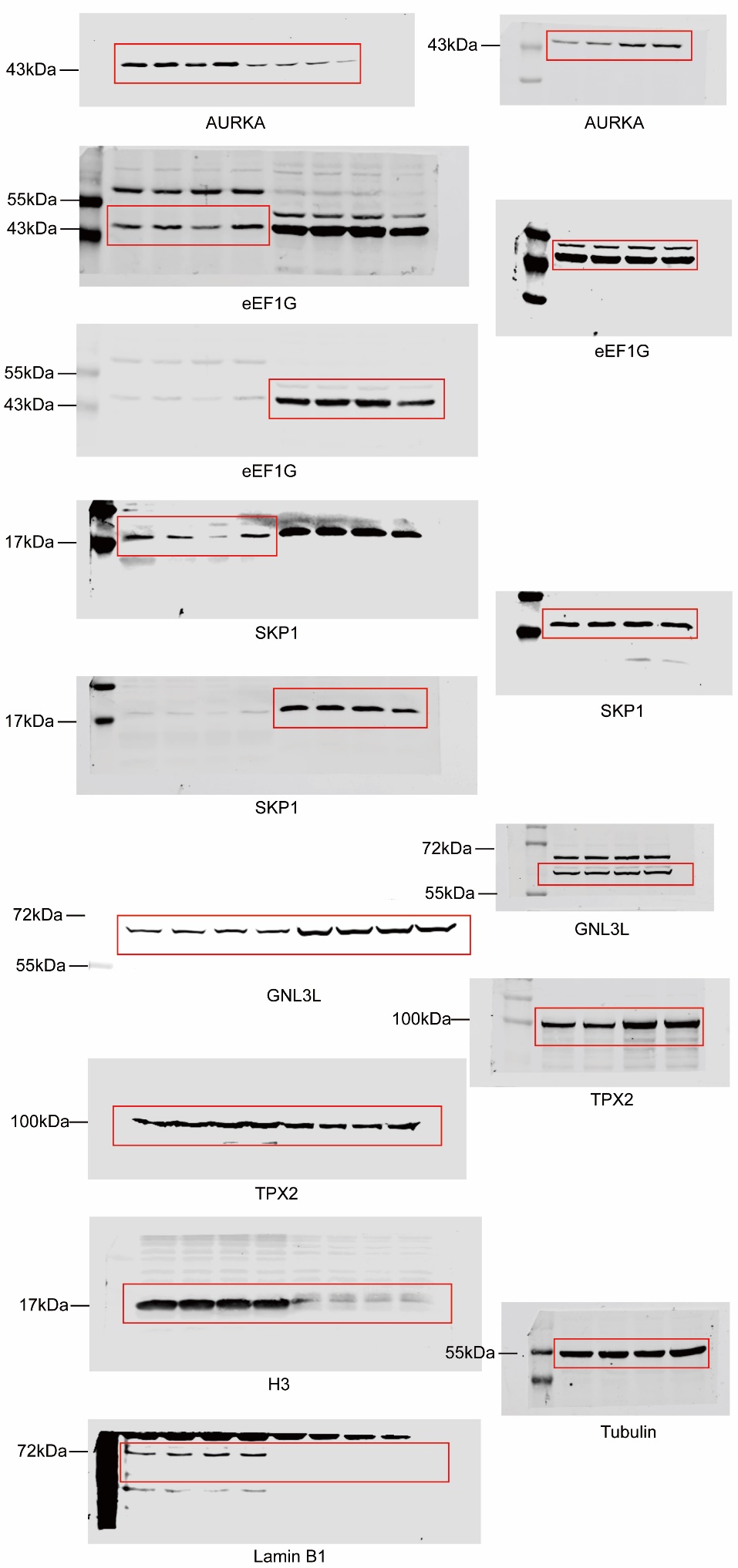


Figure 2G:


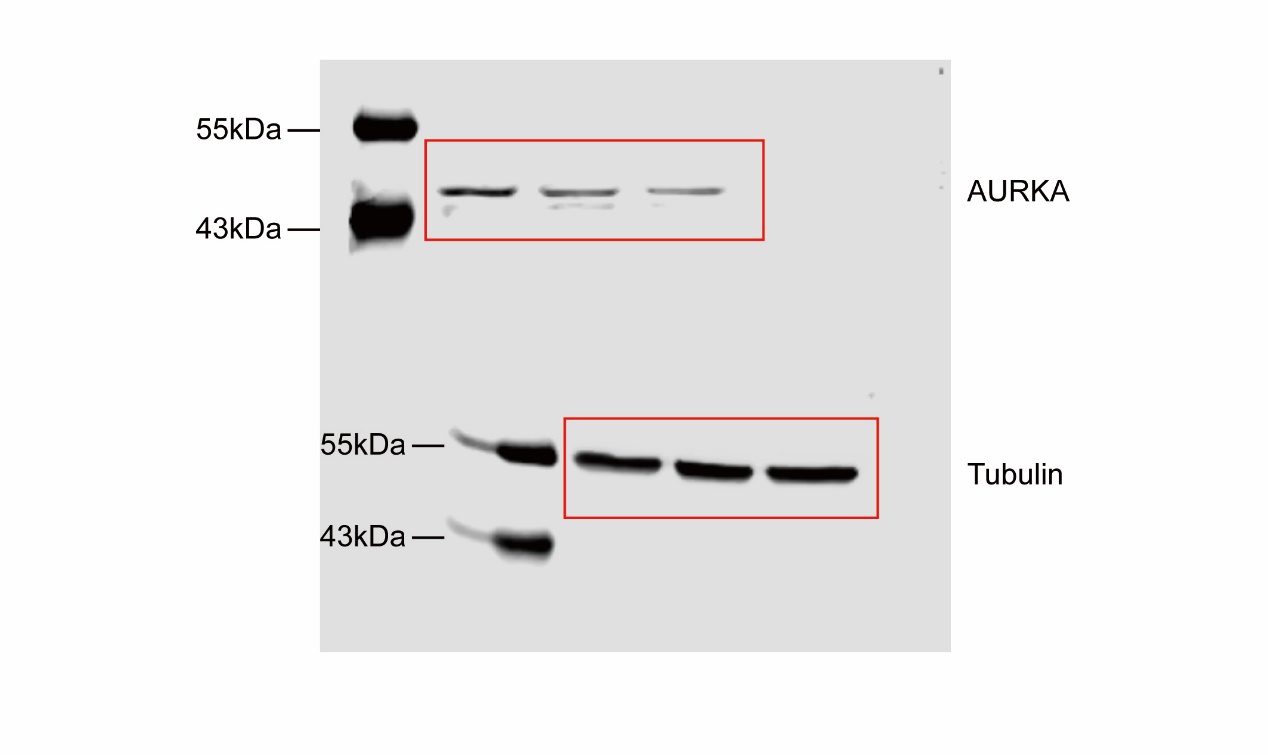


Figure 3C:


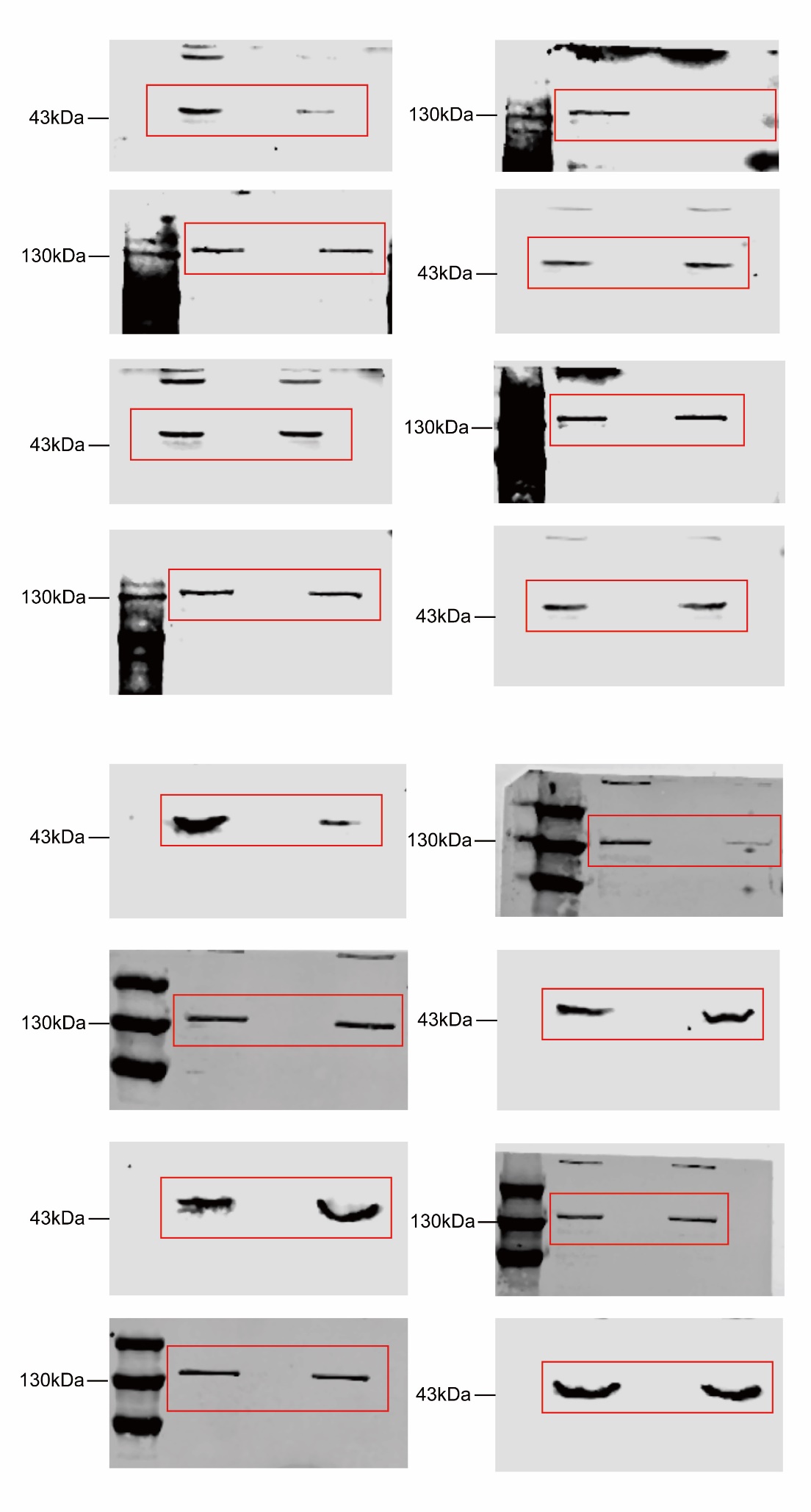


Figure 3E:


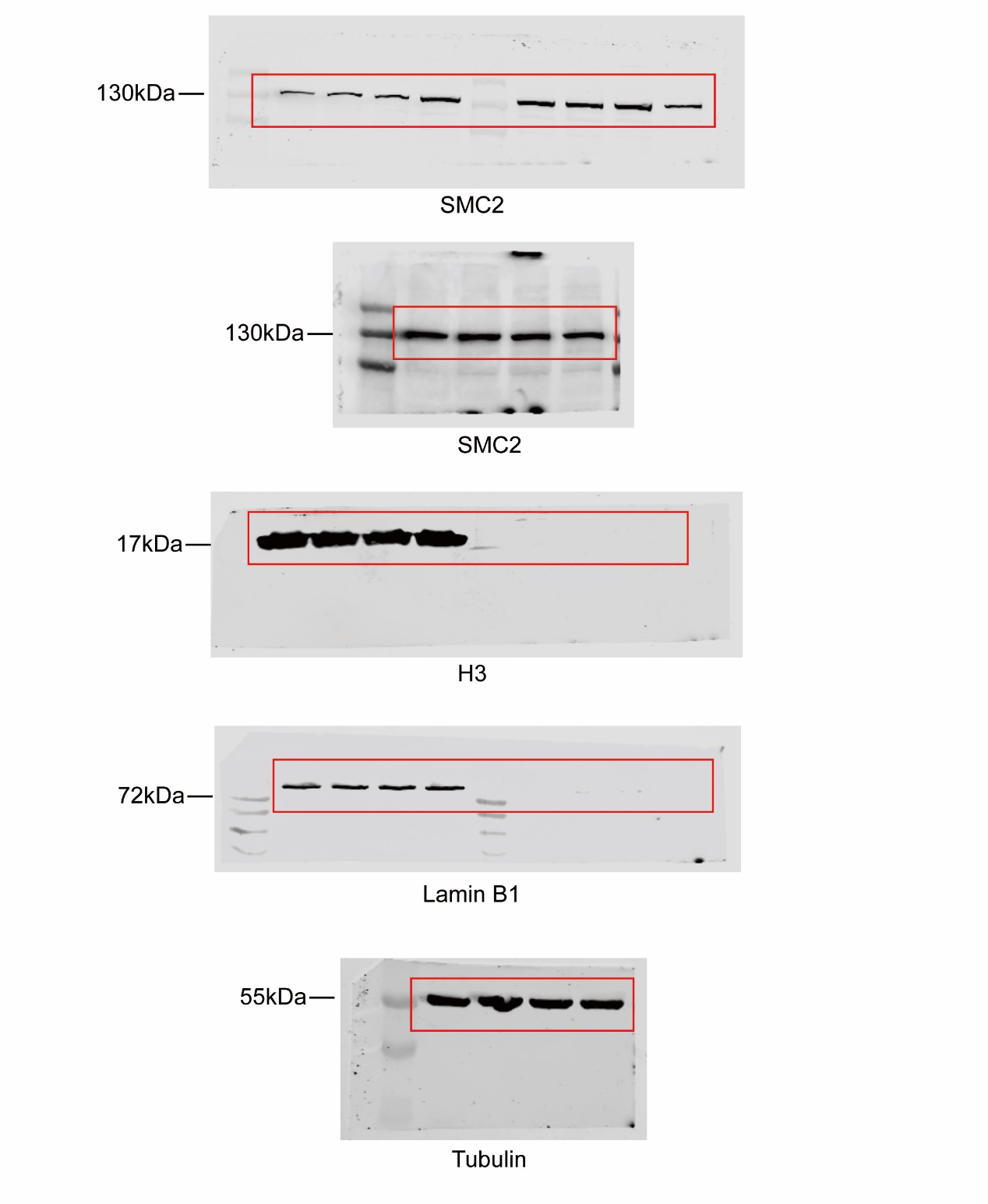


Figure 3F:


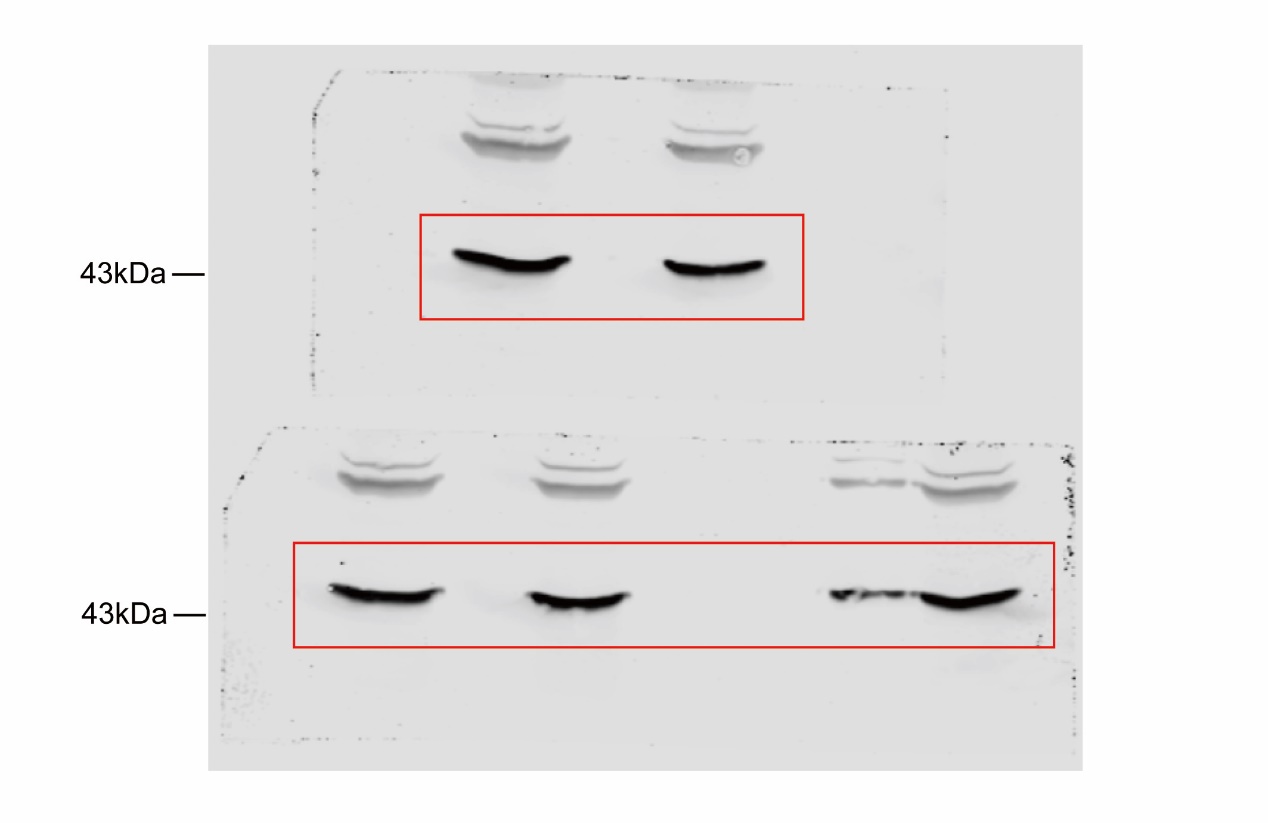


Figure 4A:


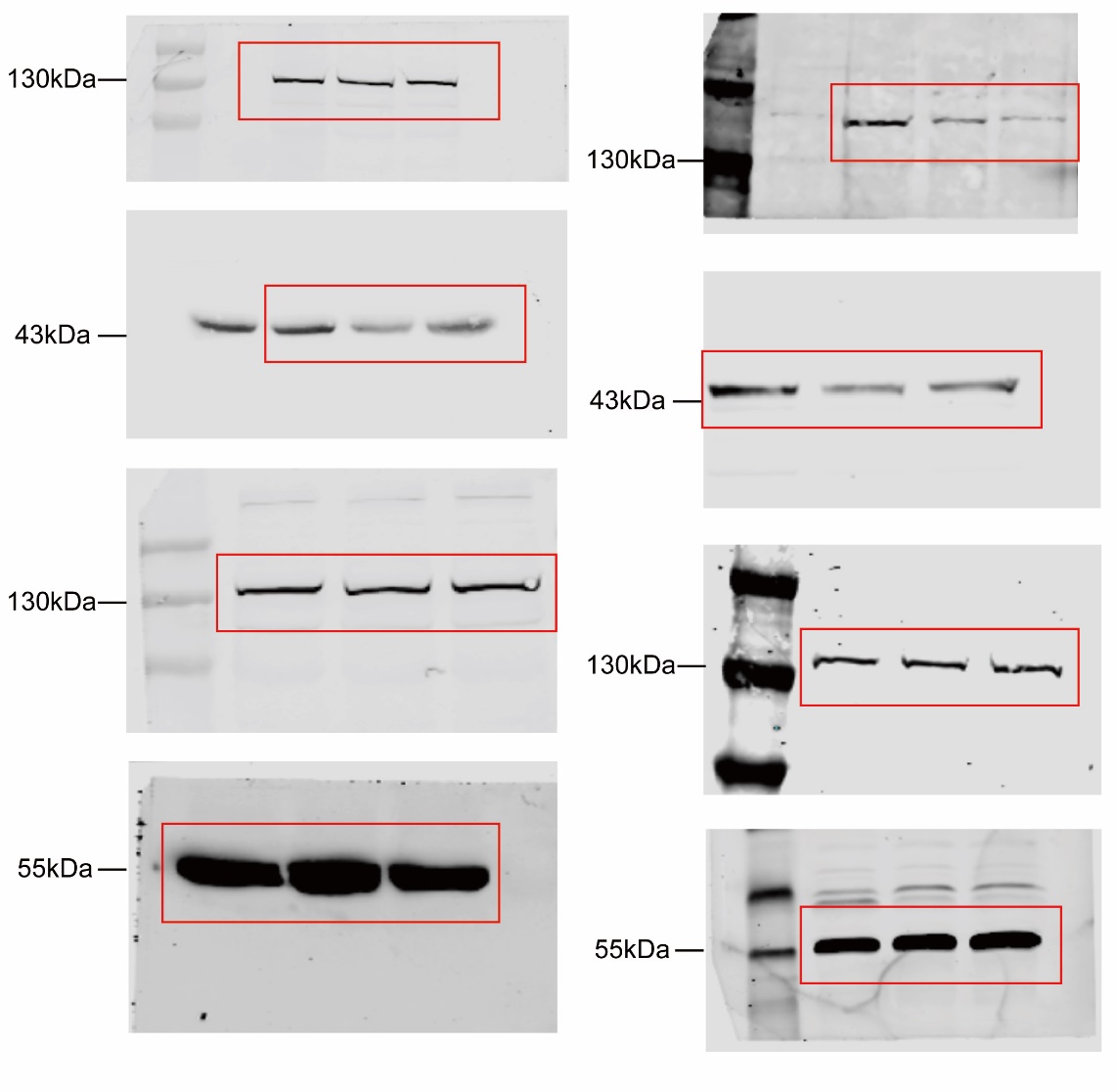


Figure 4B:


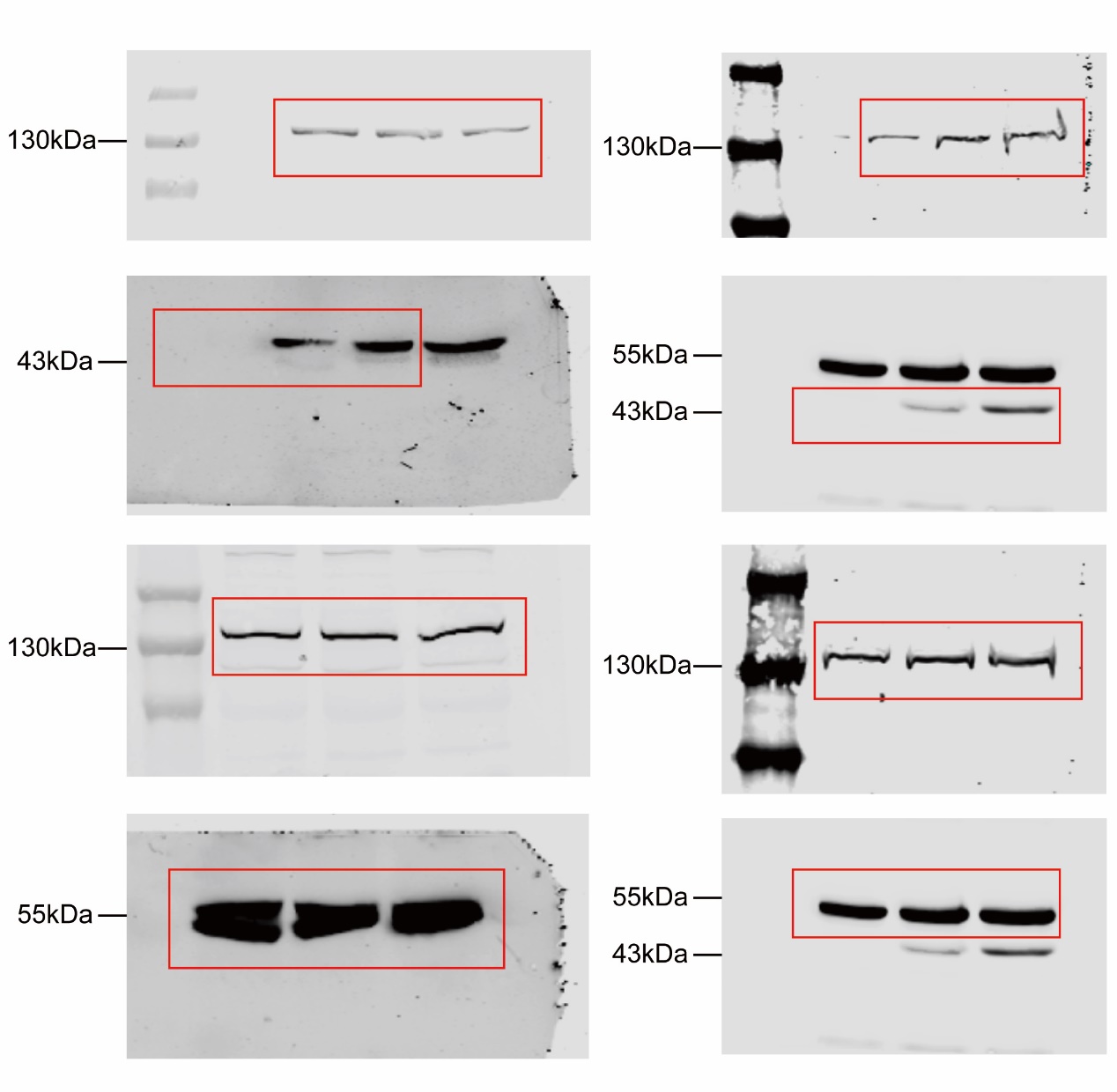


Figure 4C:


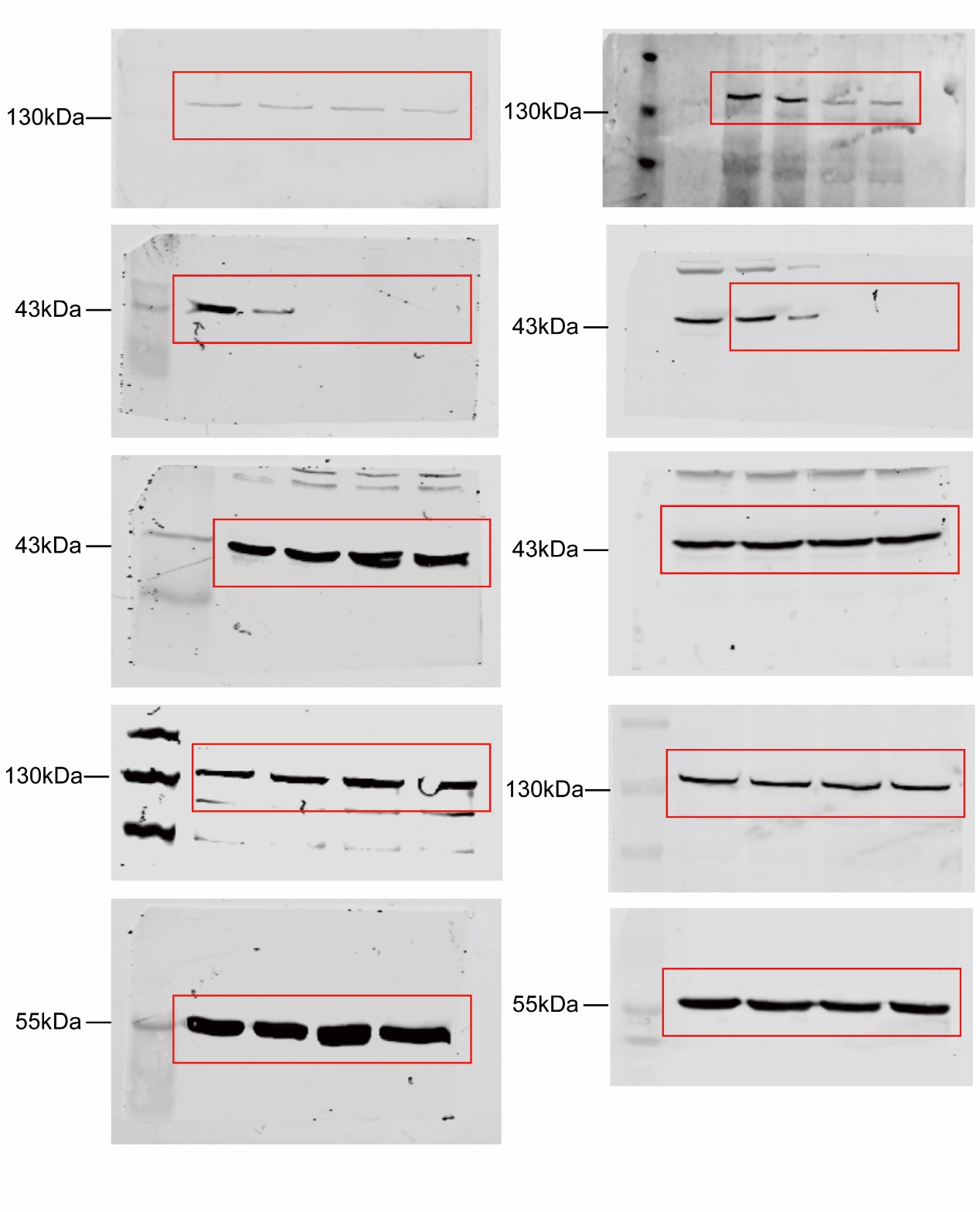


Figure 4D:


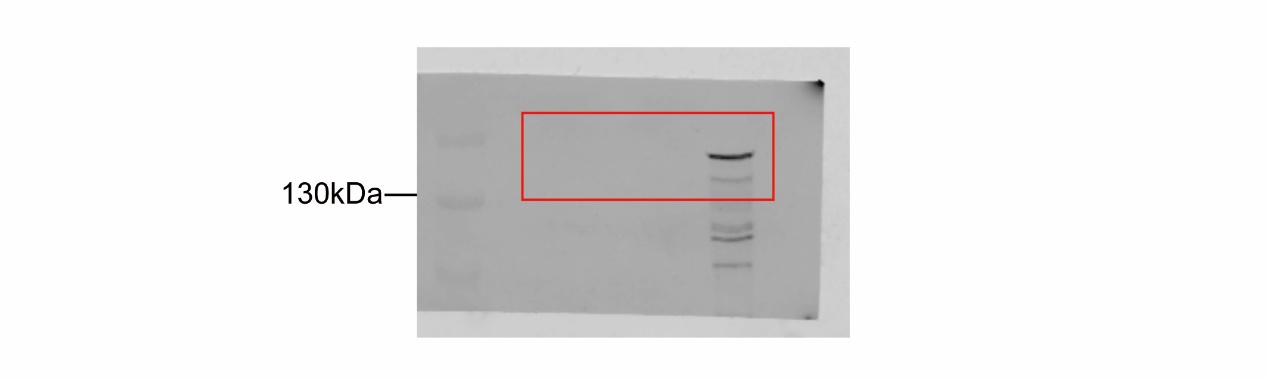


Figure 4F:


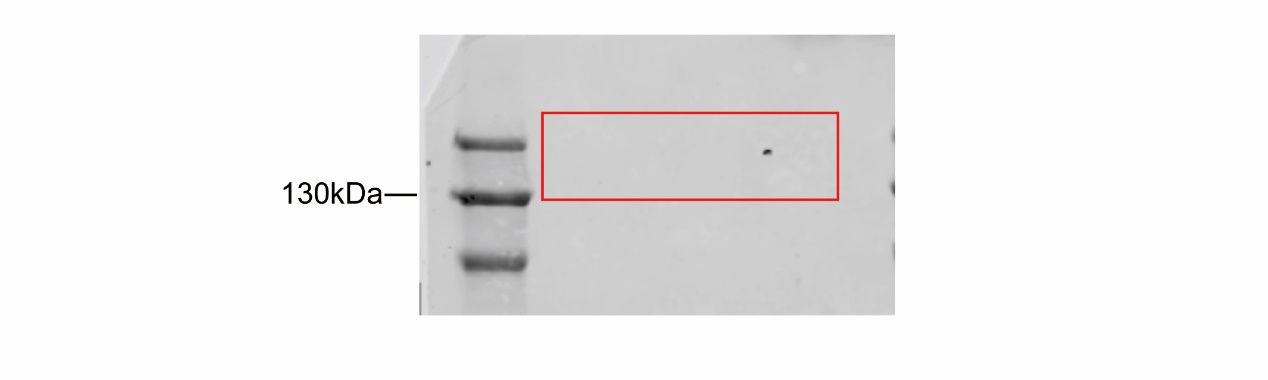


Figure 4G:


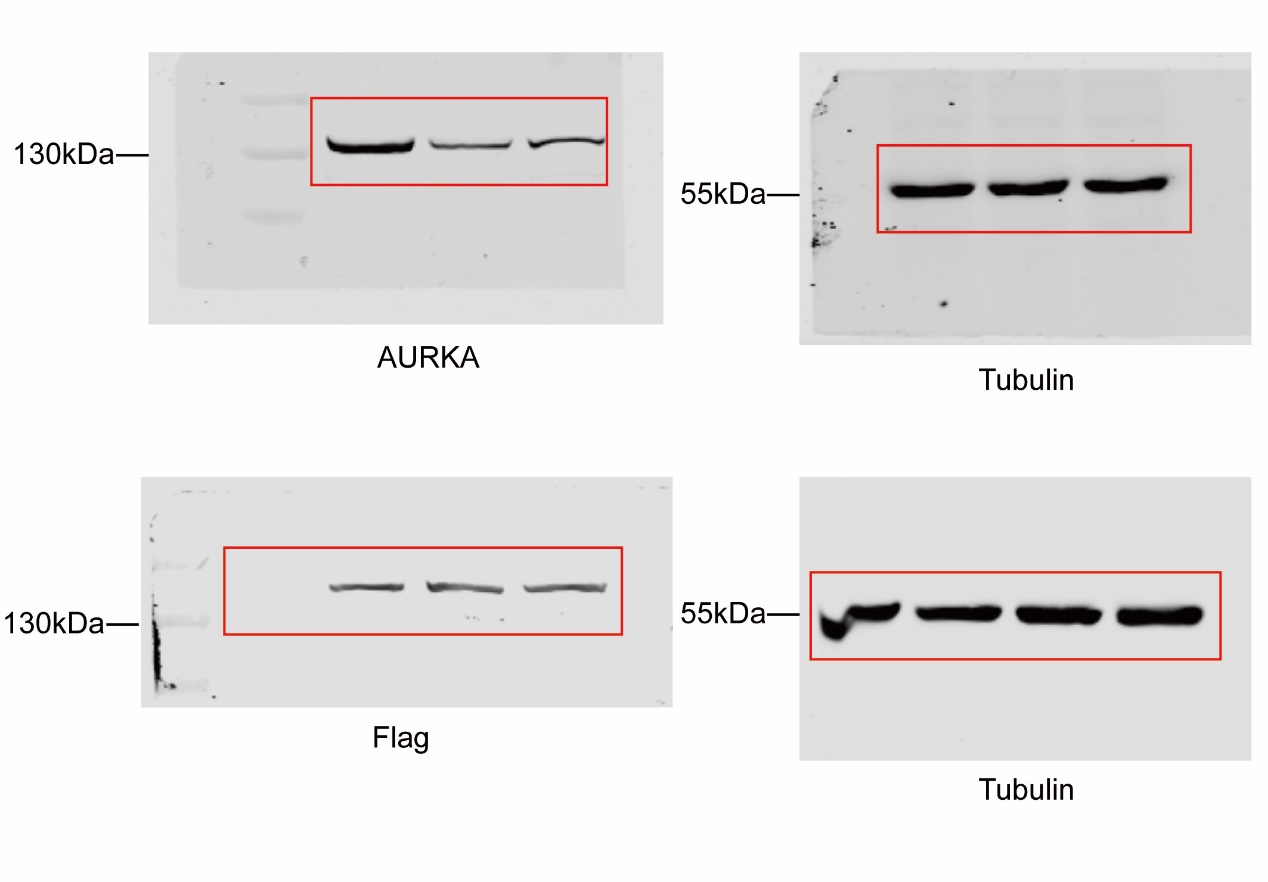


Figure 5A:


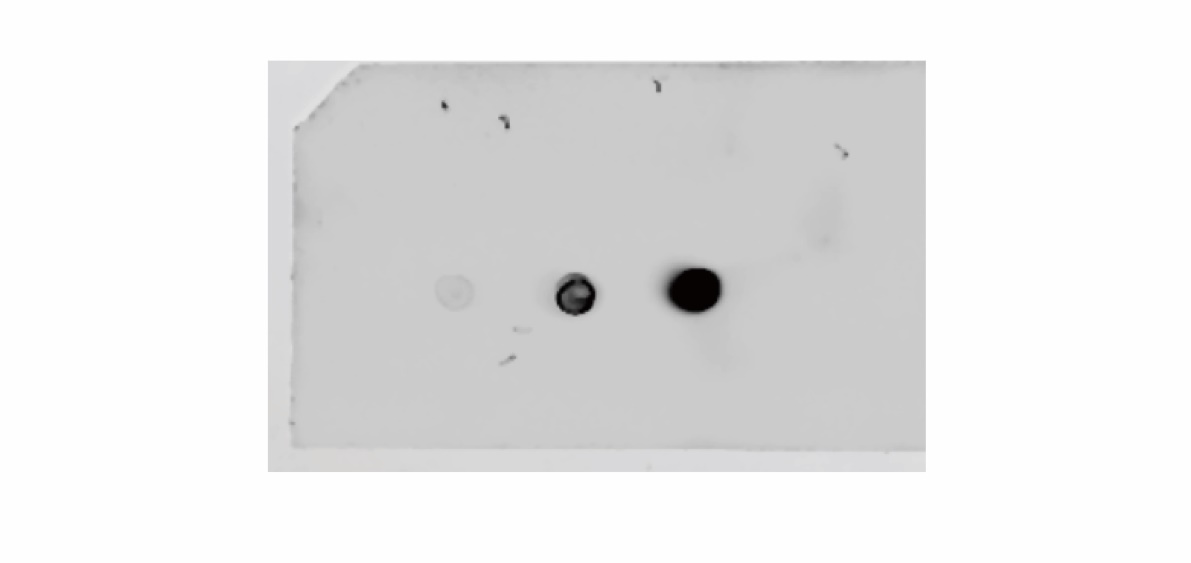


Figure 5B:


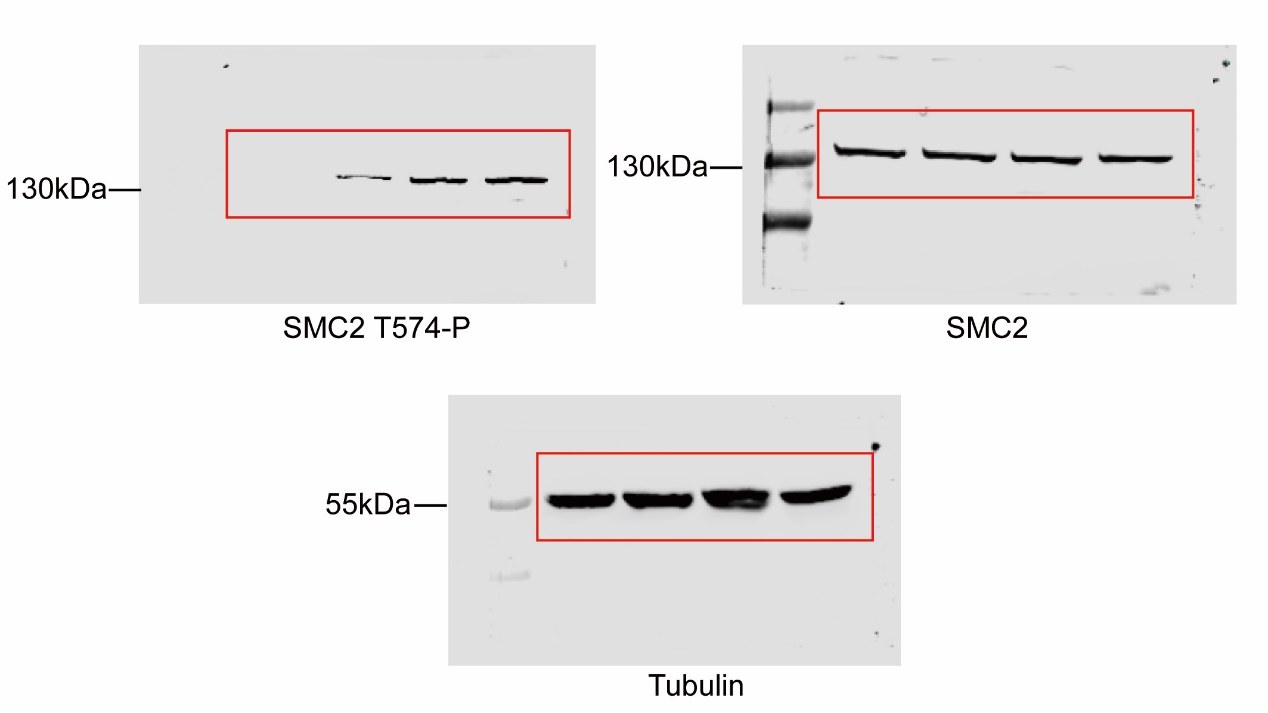


Figure 5C:


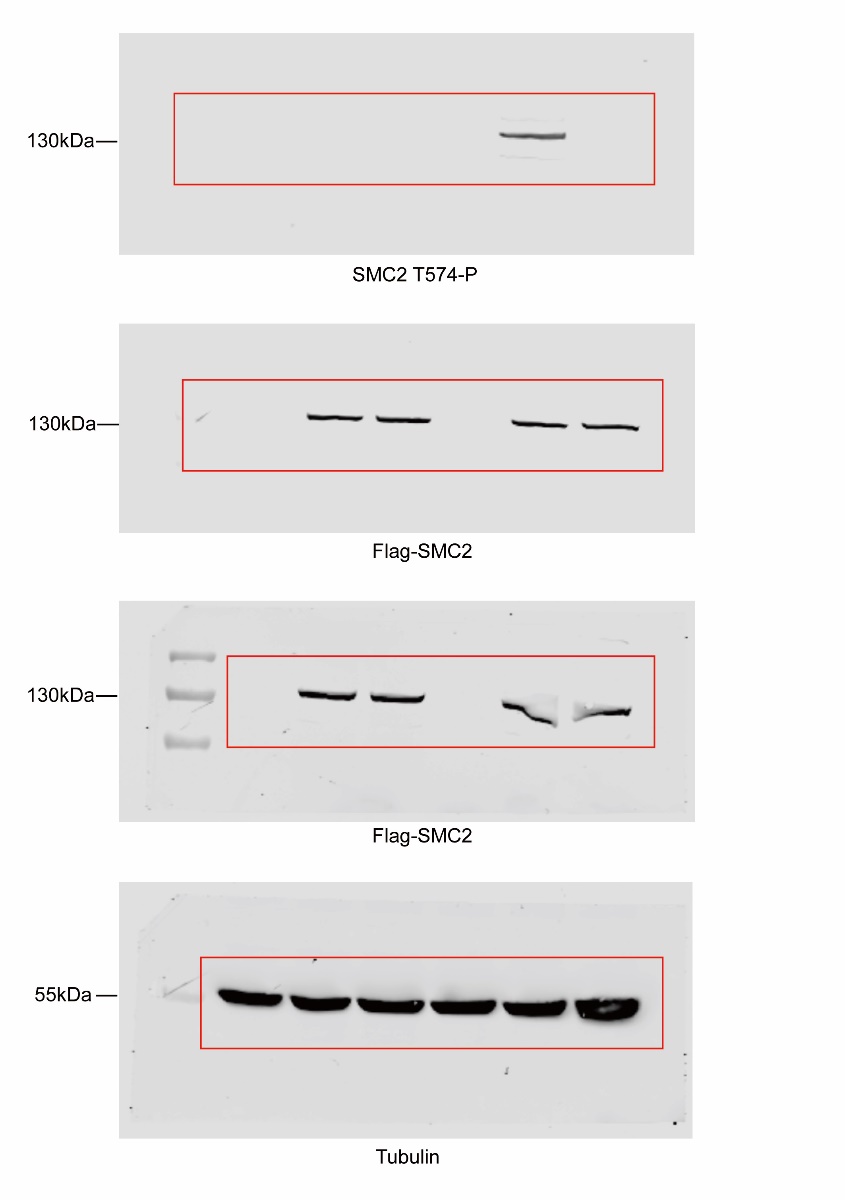


Figure 5D:


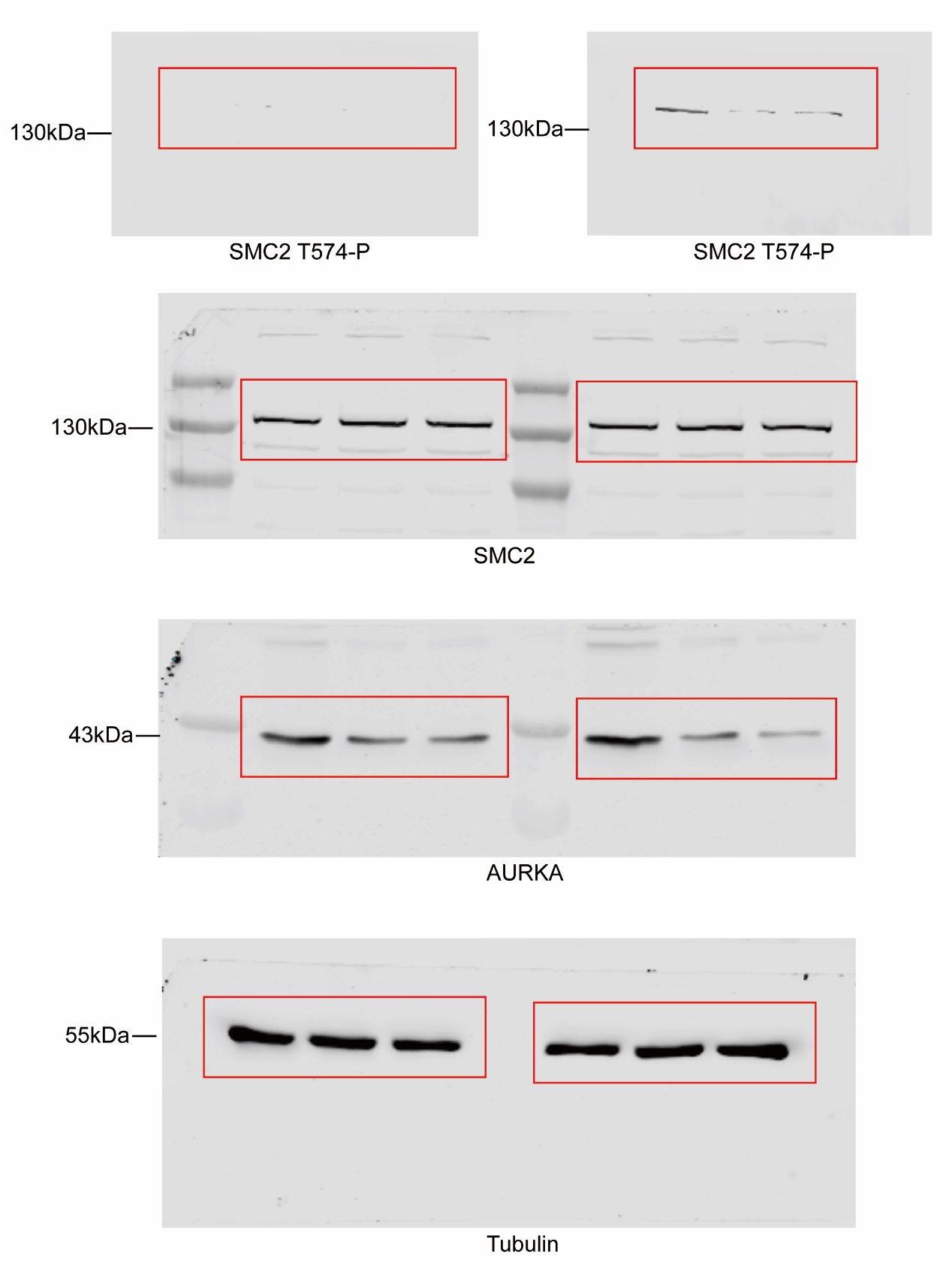


Figure 5E:


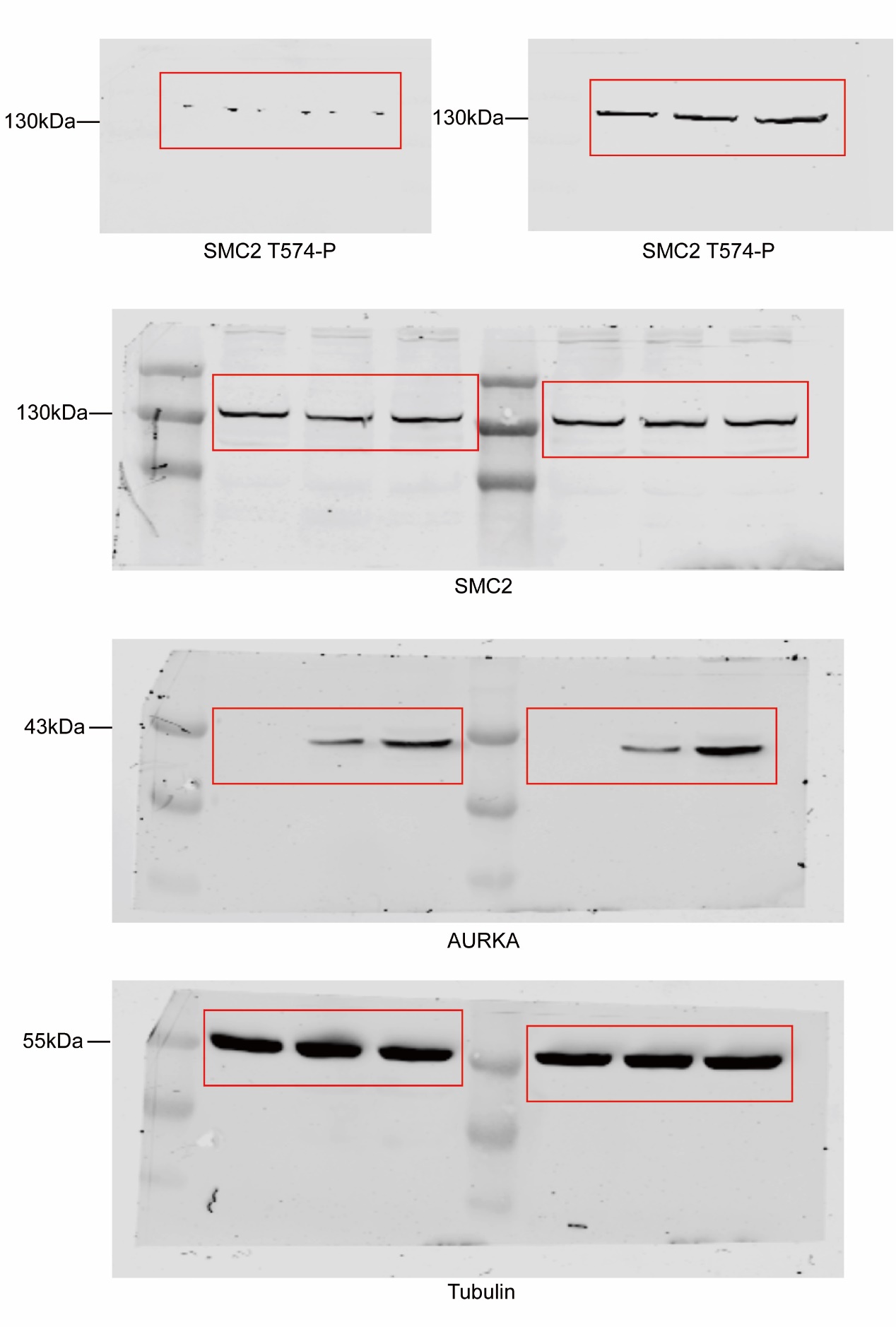


Figure 5F:


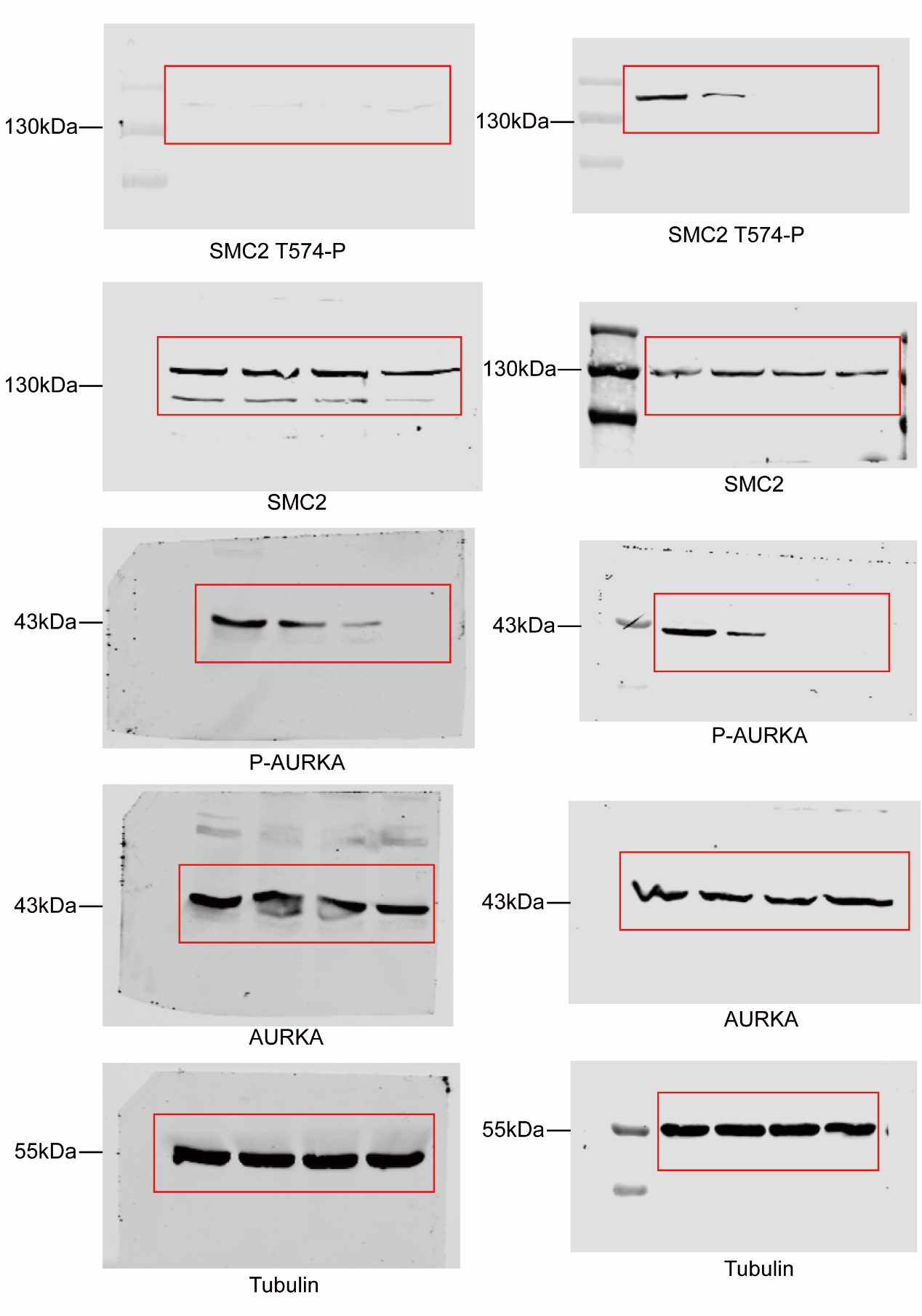


Figure 6B:


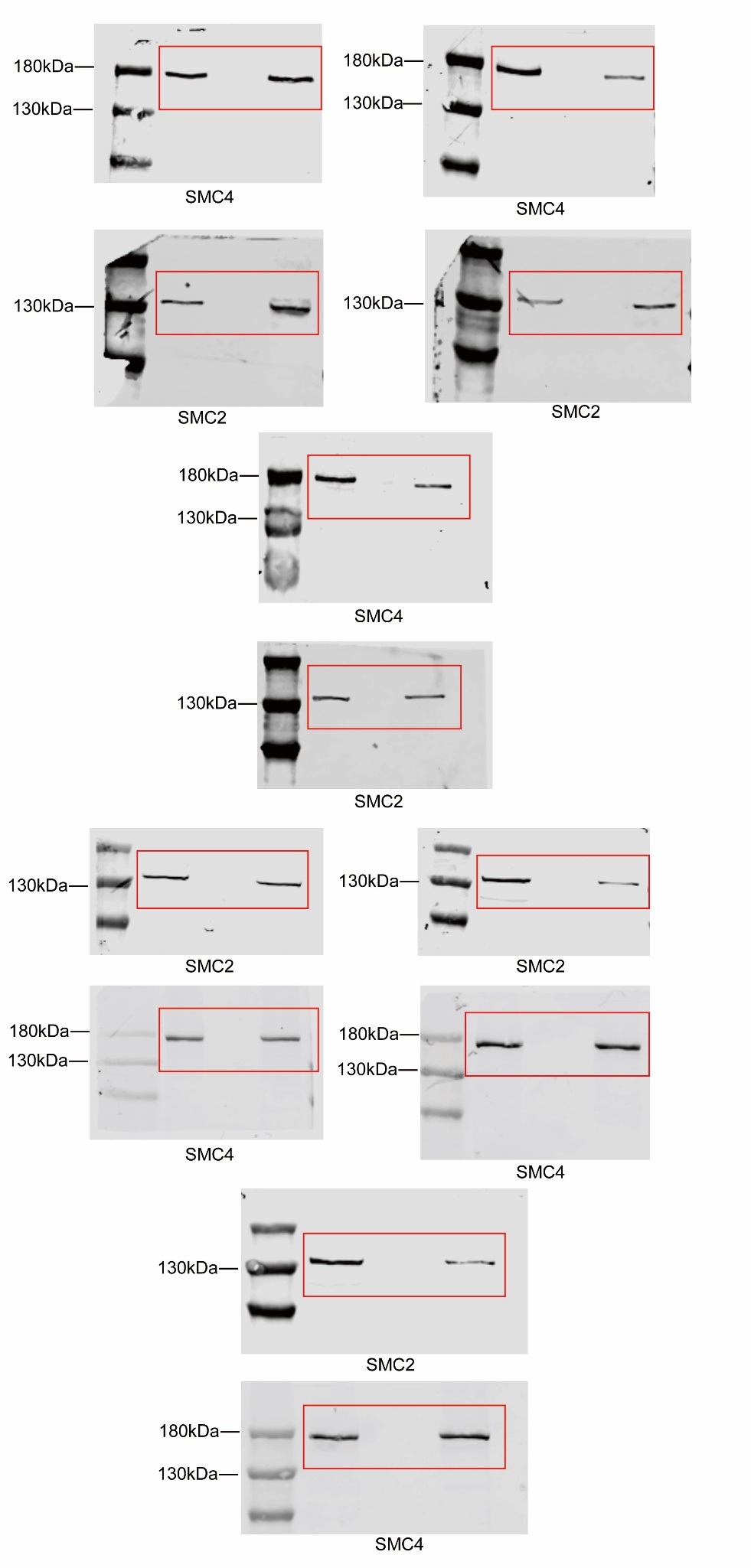


Figure 6D:


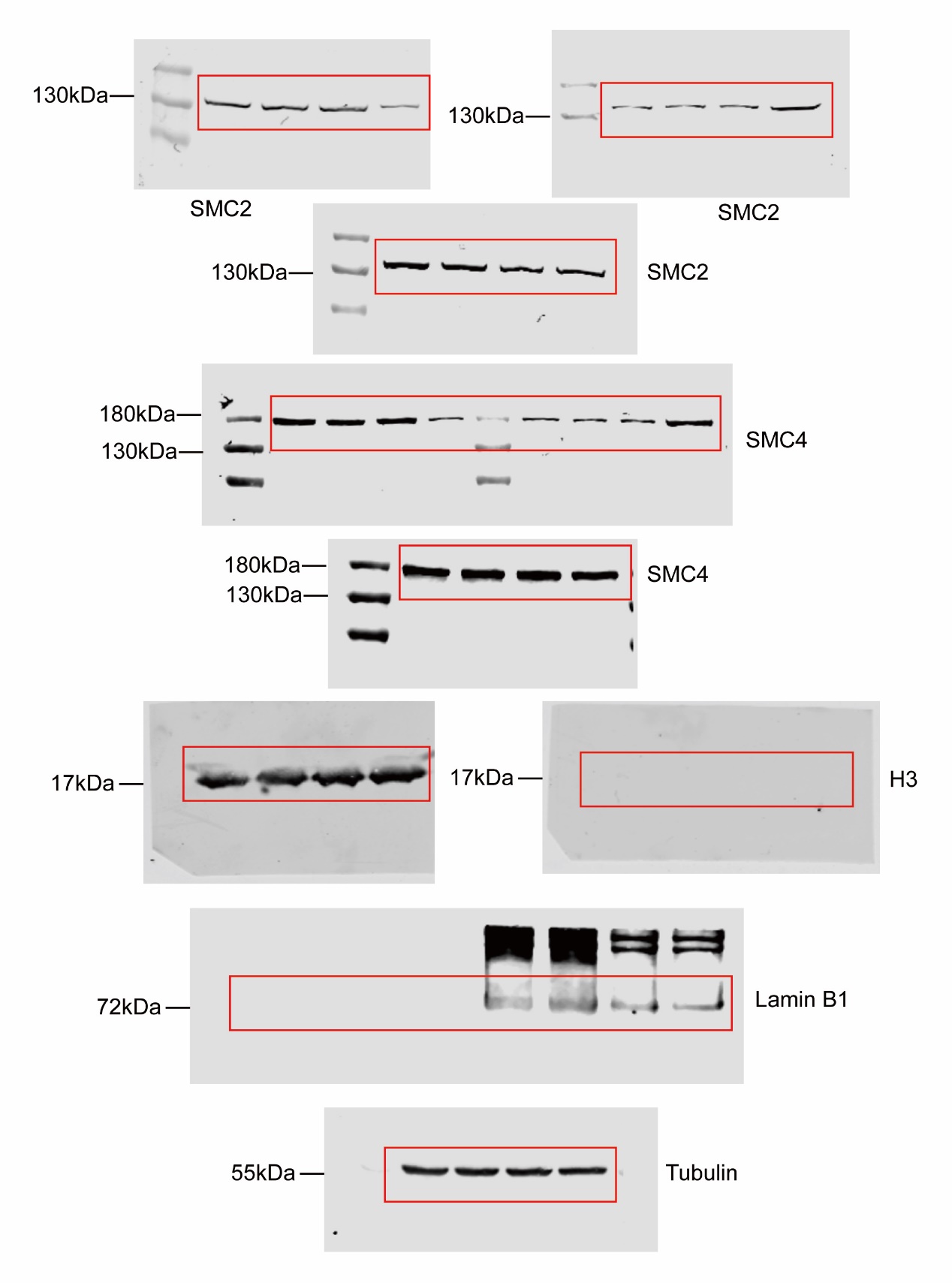


Figure 6E:


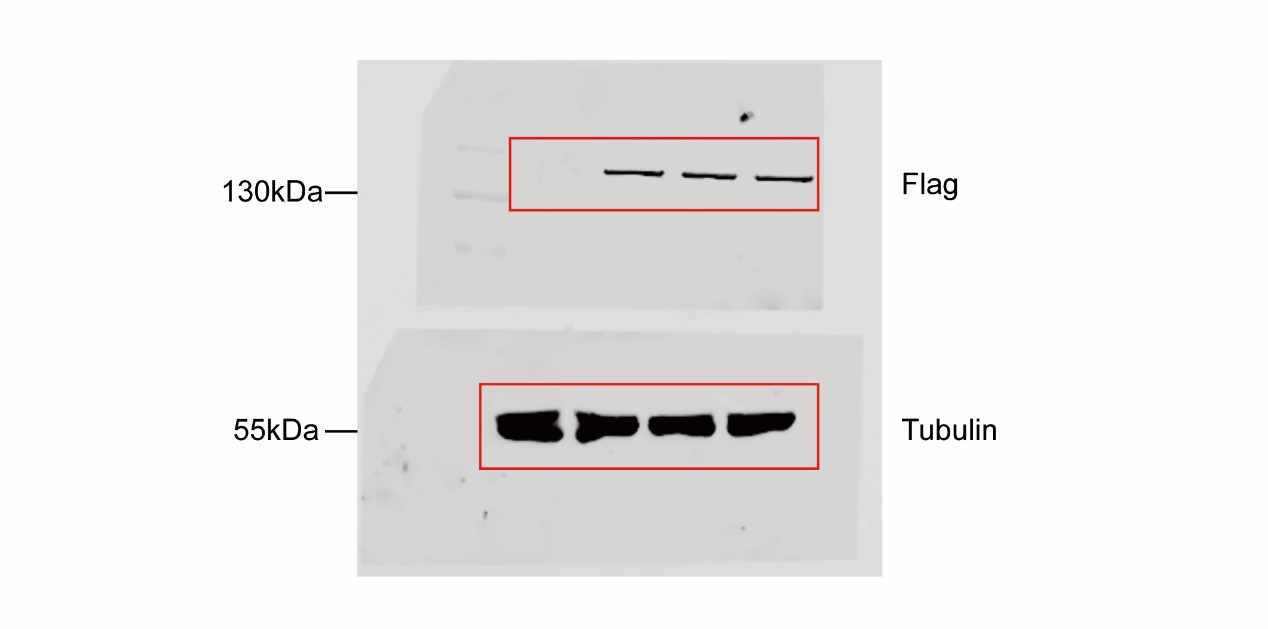


Figure 6F:


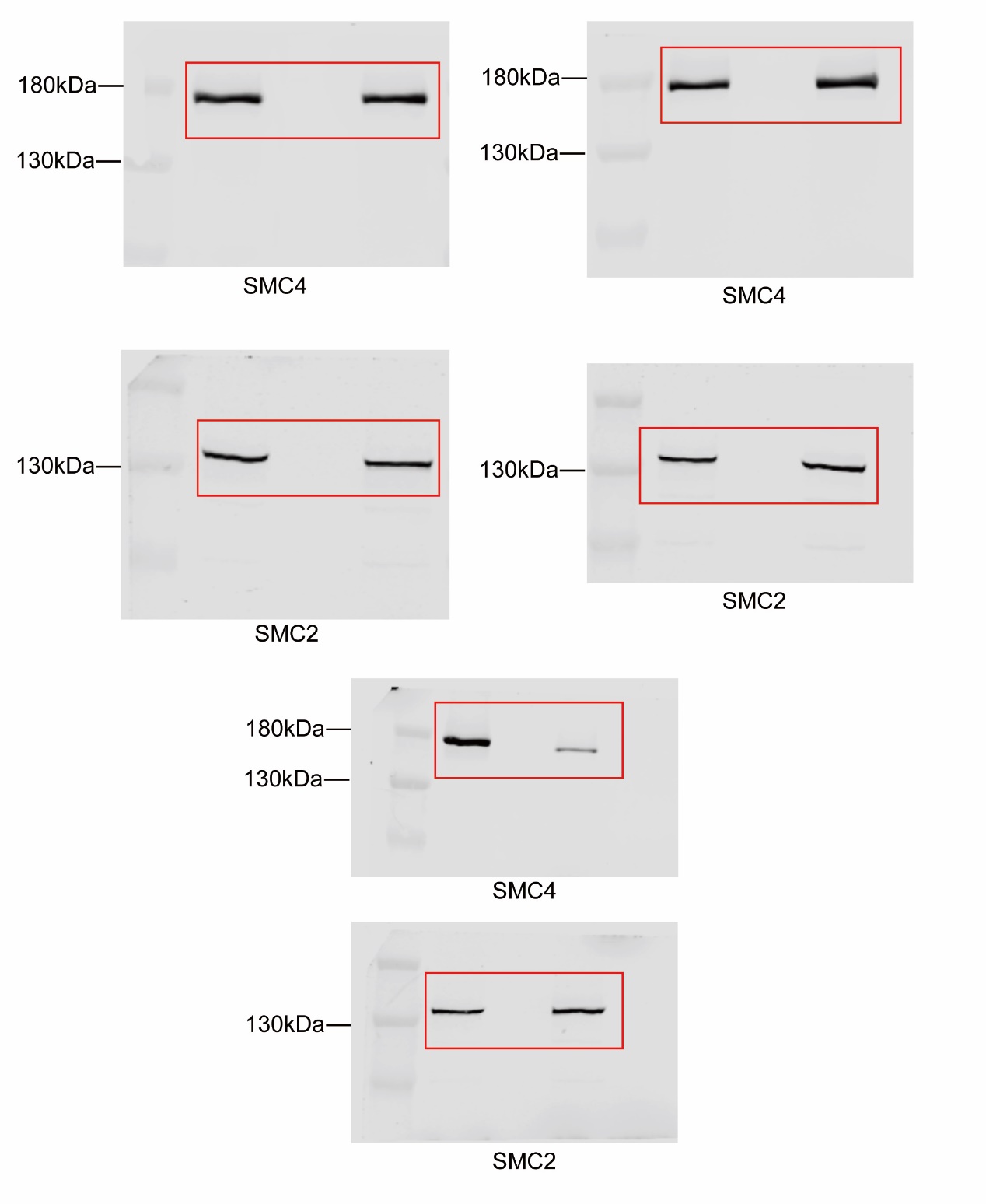


Figure S3A:


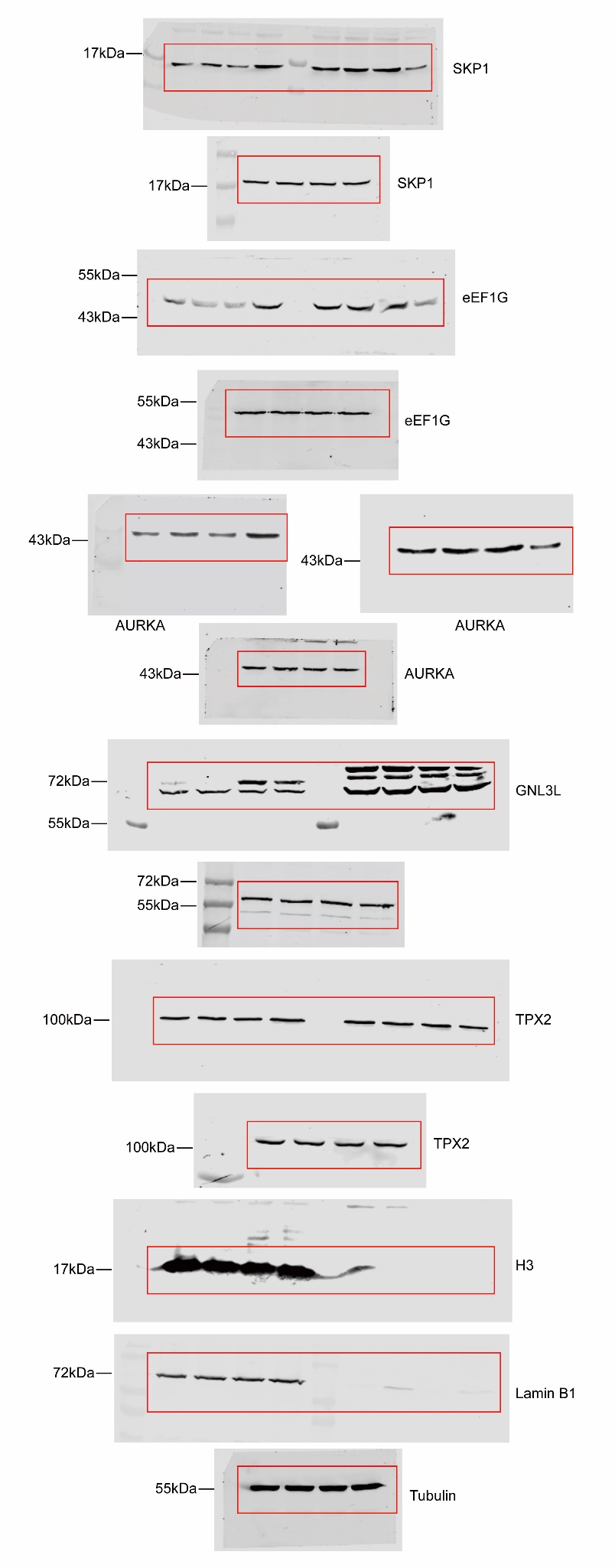


Figure S4B:


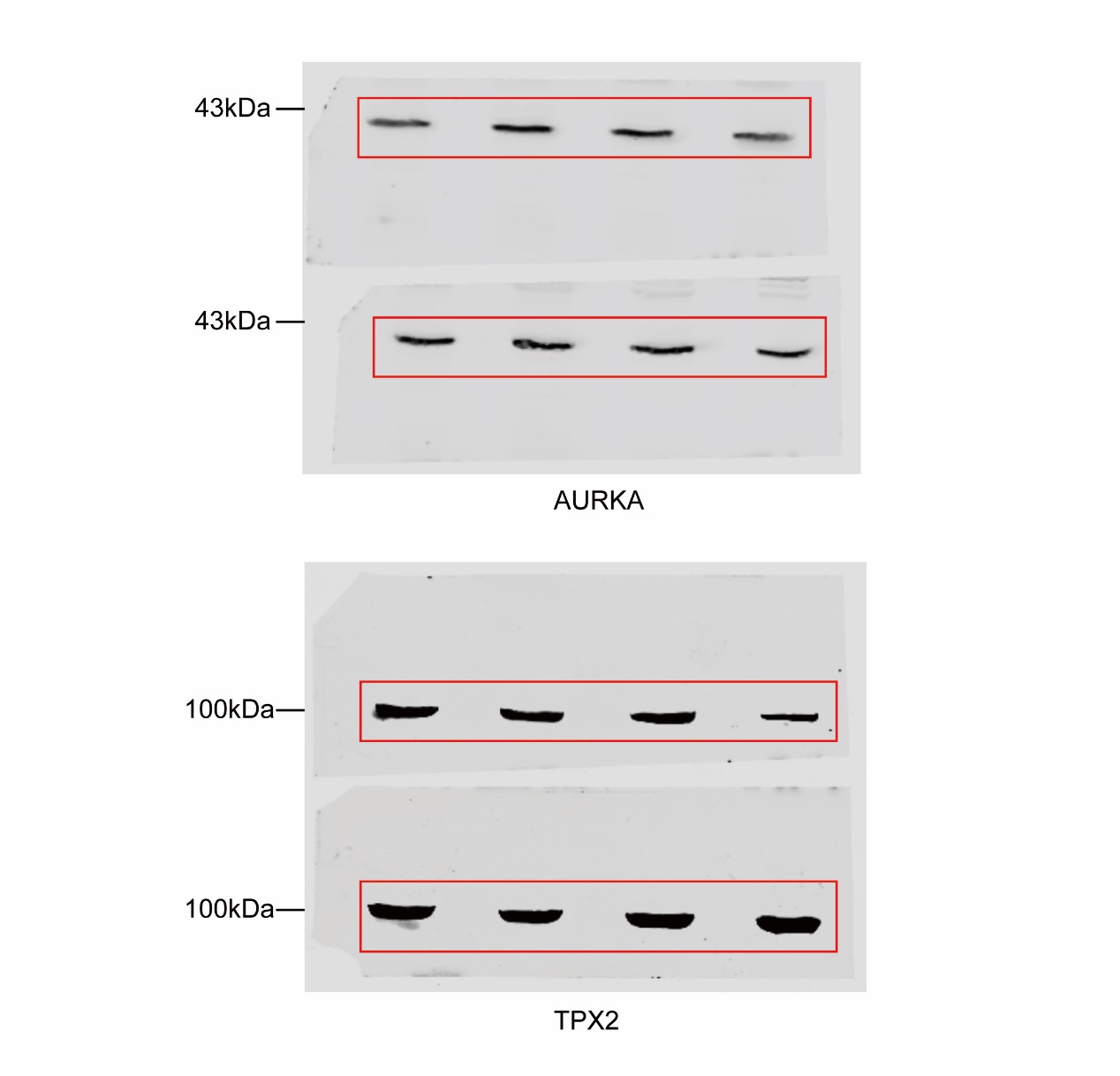


Figure S6B:


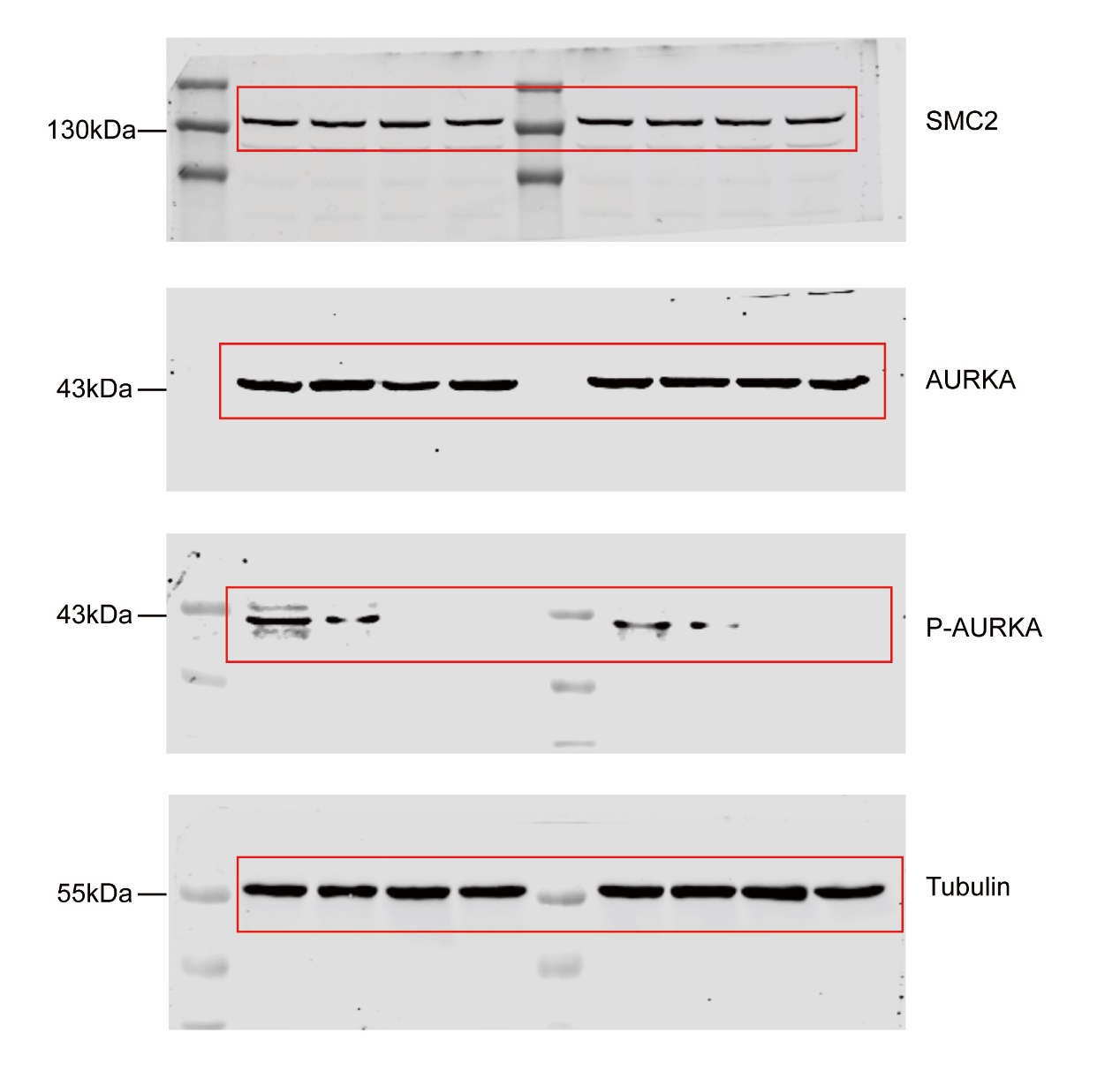


Figure S8A:


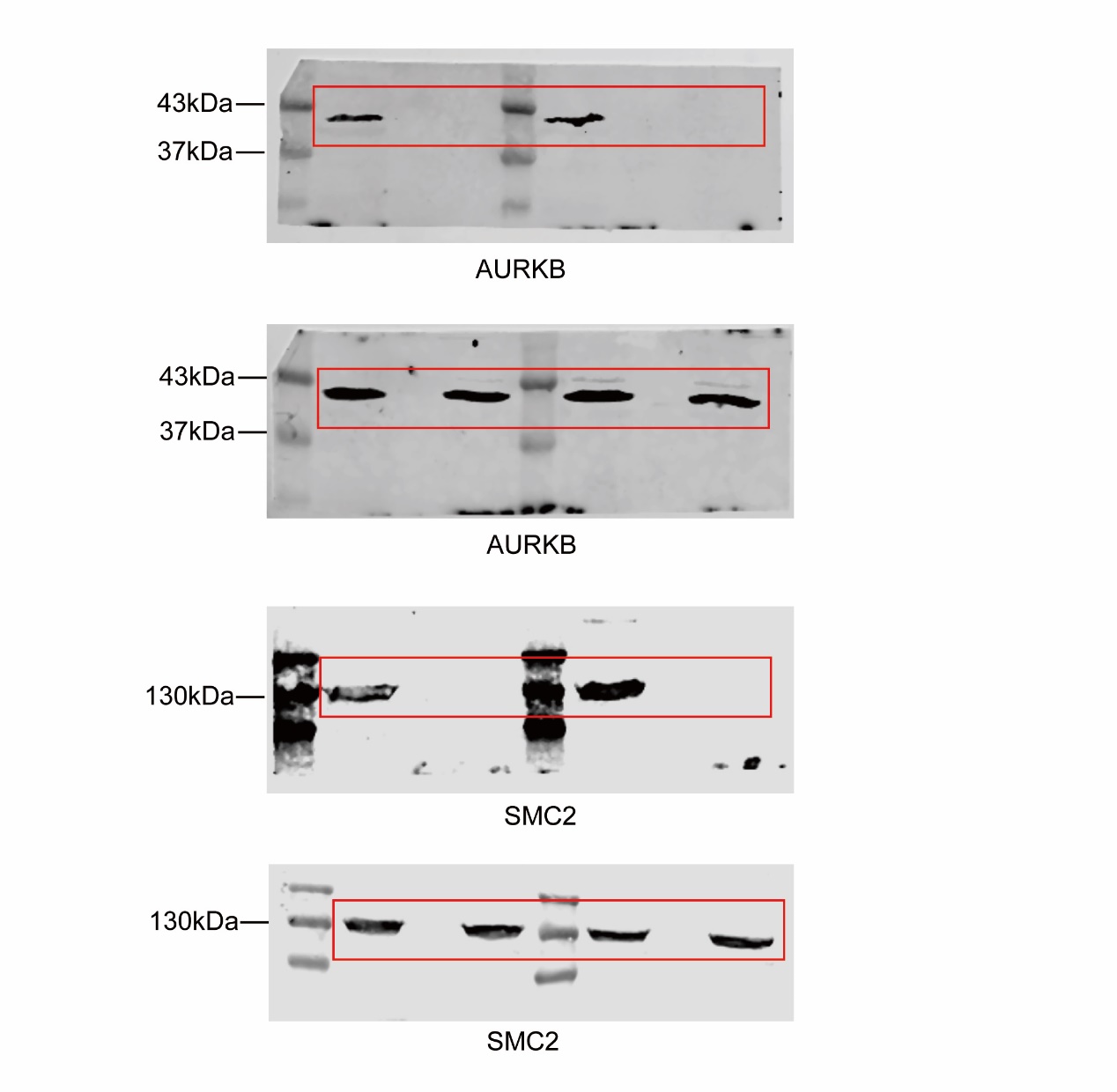


Figure S8B:


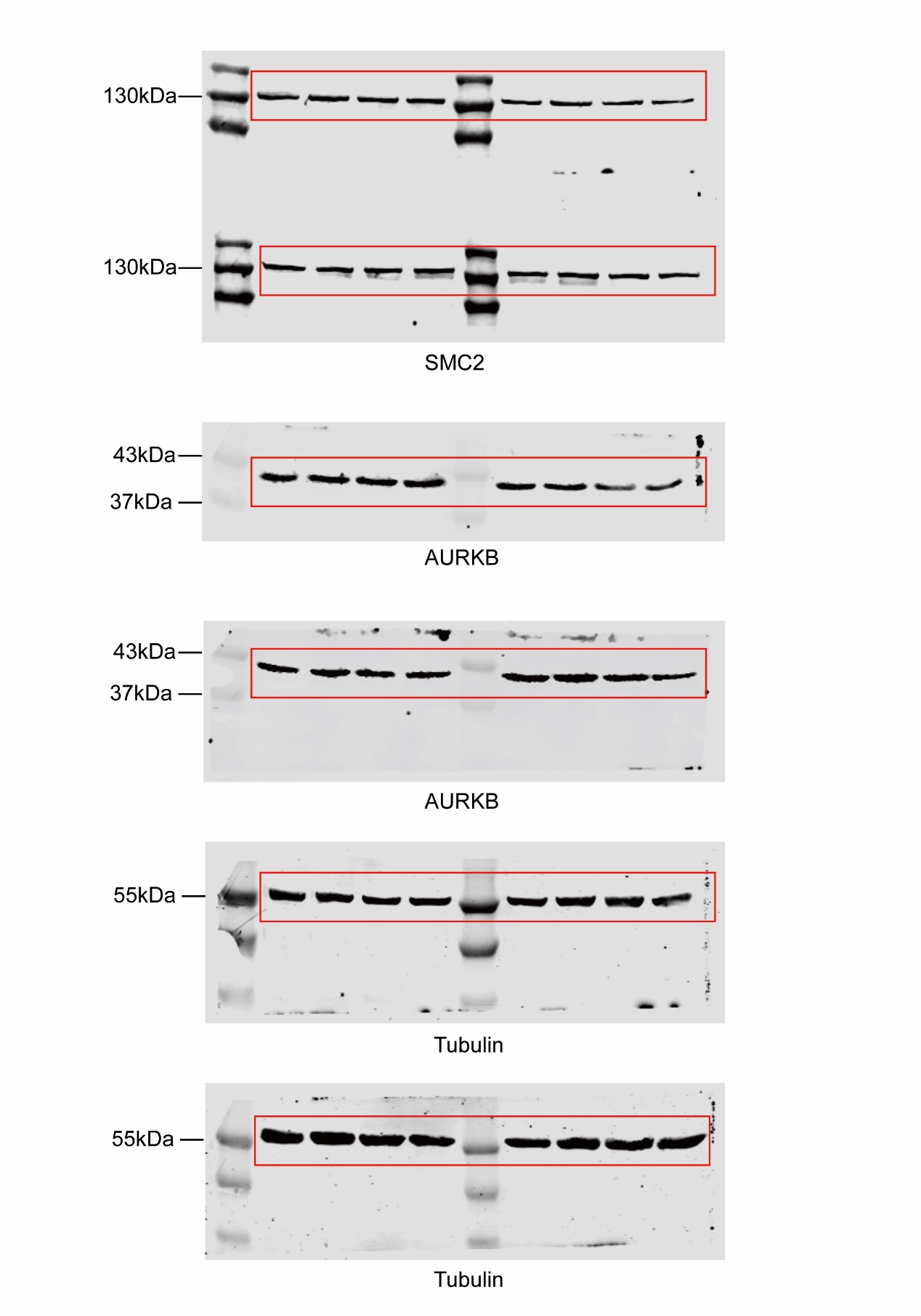


Figure S10B:


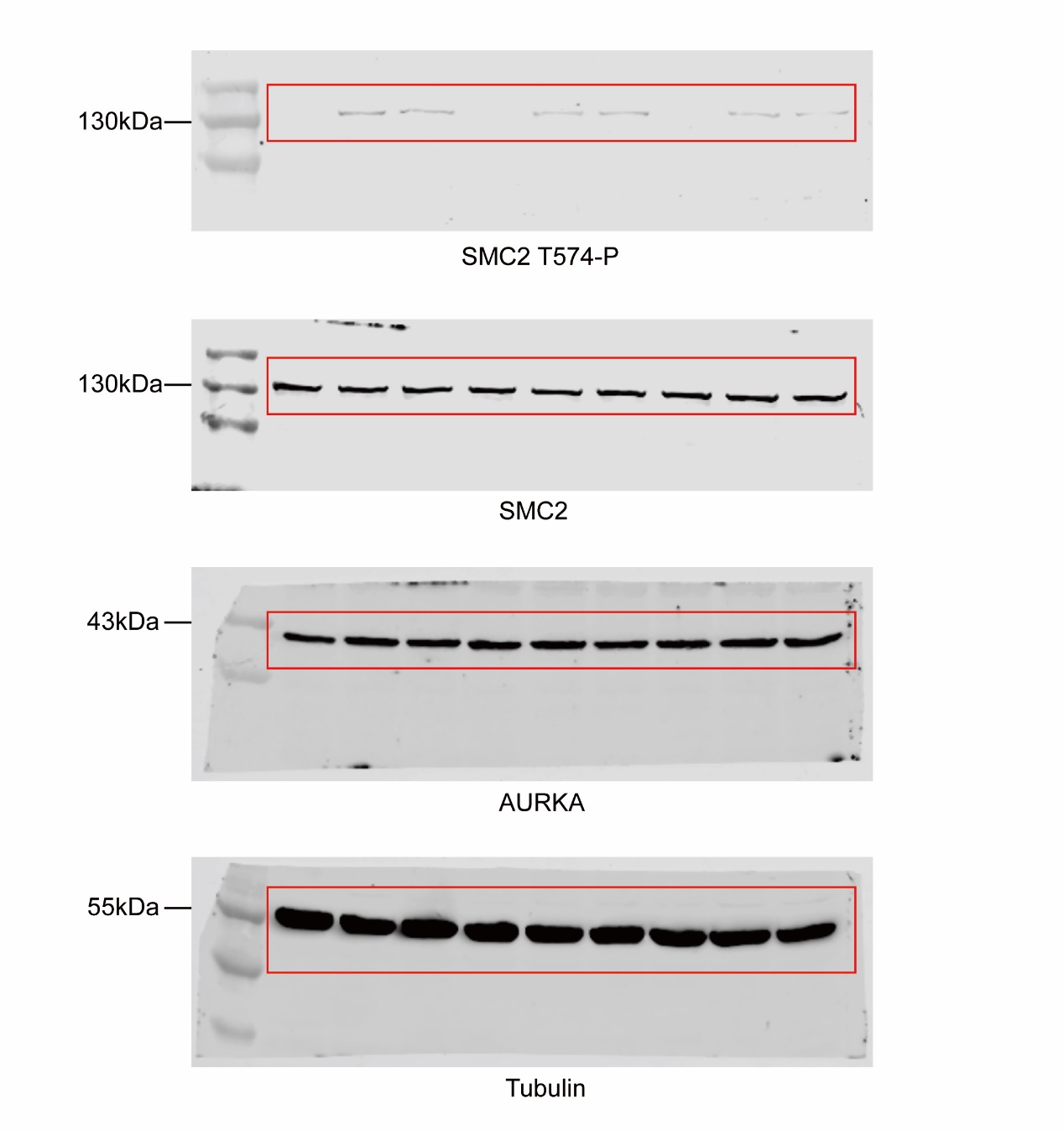

Supplement: Supplementary file 9 — Original Western blots [file 41419_2025_8169_MOESM9_ESM.docx]
